# Development and clinical application of an integrative genomic approach to personalized cancer therapy

**DOI:** 10.1186/s13073-016-0313-0

**Published:** 2016-06-01

**Authors:** Andrew V. Uzilov, Wei Ding, Marc Y. Fink, Yevgeniy Antipin, Andrew S. Brohl, Claire Davis, Chun Yee Lau, Chetanya Pandya, Hardik Shah, Yumi Kasai, James Powell, Mark Micchelli, Rafael Castellanos, Zhongyang Zhang, Michael Linderman, Yayoi Kinoshita, Micol Zweig, Katie Raustad, Kakit Cheung, Diane Castillo, Melissa Wooten, Imane Bourzgui, Leah C. Newman, Gintaras Deikus, Bino Mathew, Jun Zhu, Benjamin S. Glicksberg, Aye S. Moe, Jun Liao, Lisa Edelmann, Joel T. Dudley, Robert G. Maki, Andrew Kasarskis, Randall F. Holcombe, Milind Mahajan, Ke Hao, Boris Reva, Janina Longtine, Daniela Starcevic, Robert Sebra, Michael J. Donovan, Shuyu Li, Eric E. Schadt, Rong Chen

**Affiliations:** Department of Genetics and Genomic Sciences, Icahn Institute for Genomics and Multiscale Biology, Icahn School of Medicine at Mount Sinai, New York, NY 10029 USA; Department of Biomedical Sciences, Long Island University Post, Brookville, NY 11548 USA; Sarcoma Department, Moffitt Cancer Center, Tampa, FL 33612 USA; Department of Pathology, Icahn School of Medicine at Mount Sinai, New York, NY 10029 USA; Division of Hematology and Medical Oncology, Tisch Cancer Institute, Icahn School of Medicine at Mount Sinai, New York, NY 10029 USA

**Keywords:** Cancer, Genomics, Personalized medicine, Clinical application

## Abstract

**Background:**

Personalized therapy provides the best outcome of cancer care and its implementation in the clinic has been greatly facilitated by recent convergence of enormous progress in basic cancer research, rapid advancement of new tumor profiling technologies, and an expanding compendium of targeted cancer therapeutics.

**Methods:**

We developed a personalized cancer therapy (PCT) program in a clinical setting, using an integrative genomics approach to fully characterize the complexity of each tumor. We carried out whole exome sequencing (WES) and single-nucleotide polymorphism (SNP) microarray genotyping on DNA from tumor and patient-matched normal specimens, as well as RNA sequencing (RNA-Seq) on available frozen specimens, to identify somatic (tumor-specific) mutations, copy number alterations (CNAs), gene expression changes, gene fusions, and also germline variants. To provide high sensitivity in known cancer mutation hotspots, Ion AmpliSeq Cancer Hotspot Panel v2 (CHPv2) was also employed. We integrated the resulting data with cancer knowledge bases and developed a specific workflow for each cancer type to improve interpretation of genomic data.

**Results:**

We returned genomics findings to 46 patients and their physicians describing somatic alterations and predicting drug response, toxicity, and prognosis. Mean 17.3 cancer-relevant somatic mutations per patient were identified, 13.3-fold, 6.9-fold, and 4.7-fold more than could have been detected using CHPv2, Oncomine Cancer Panel (OCP), and FoundationOne, respectively. Our approach delineated the underlying genetic drivers at the pathway level and provided meaningful predictions of therapeutic efficacy and toxicity. Actionable alterations were found in 91 % of patients (mean 4.9 per patient, including somatic mutations, copy number alterations, gene expression alterations, and germline variants), a 7.5-fold, 2.0-fold, and 1.9-fold increase over what could have been uncovered by CHPv2, OCP, and FoundationOne, respectively. The findings altered the course of treatment in four cases.

**Conclusions:**

These results show that a comprehensive, integrative genomic approach as outlined above significantly enhanced genomics-based PCT strategies.

**Electronic supplementary material:**

The online version of this article (doi:10.1186/s13073-016-0313-0) contains supplementary material, which is available to authorized users.

## Background

Personalizing cancer therapy is a well-established concept, given that every patient harbors a unique constellation of variants that influence the risk, onset, and progression of their disease. For every specific type and stage of cancer, clinical manifestations differ between individuals, showing variations in tumor behavior and progression as well as variations in responses to a given treatment regimen, largely driven by the unique genomic (DNA, RNA, and epigenetic) makeup of the individual tumors. Developing a personalized therapy strategy to ensure an optimal outcome for individual cancer patients is possible given the dramatic progress in basic cancer research at the molecular and cellular levels, the rapid advancement of new technologies that enable fast and cost-effective comprehensive characterizations of tumors at the molecular level, and an expanding compendium of targeted cancer therapeutics.

Many FDA-approved targeted cancer drugs have pharmacogenomic labels that include biomarkers predictive of drug response, in addition to germline variants that are associated with drug metabolism or may impact treatment response [1]. In cases where a patient tests positive for a specific biomarker that indicates an FDA-approved therapy for the given tumor type, developing a personalized therapeutic strategy is straightforward. For example, crizotinib is indicated as a treatment for non-small cell lung cancer tumors harboring ALK gene translocations and vemurafenib is indicated as a treatment for metastatic melanoma tumors harboring the BRAF p.V600E mutation. However, for the vast majority of tumor types and available therapeutics, a biomarker-therapeutic link is not straightforward.

The rapid development of next-generation sequencing (NGS) technologies as a high-throughput, low-cost way of generating whole genome (WGS) and whole exome (WES) sequence data has enabled a new paradigm in precision medicine for oncology. Large-scale NGS studies over the last several years have uncovered novel oncogenic drivers and started to depict genetic landscapes across a number of cancer types [2–4]. This research advanced the understanding of the underlying genetics of cancer and enabled acceleration of personalized cancer therapy (PCT) [5]. Retrospective analyses of archived tumor samples using targeted gene panels or WES have been reported [6–9]. Actionable mutations were identified in 80–90 % of the tumor samples in these studies. A number of prospective studies have also demonstrated the clinical utility of NGS-based cancer genetic testing. One pilot study generated low-depth WGS, WES, and RNA sequencing (RNA-Seq) data on four patients with advanced cancers, and these genomic data for two patients were reviewed by a molecular tumor board to deliver clinical recommendations [10]. WES of formalin-fixed, paraffin-embedded (FFPE) tumor samples has been recently reported suggesting that comprehensive exome sequencing provides a complete spectrum of clinically relevant genetic alterations, as demonstrated in one case where previously undetected genetic alterations led to clinical trial enrollment and objective clinical response [11]. WES of tumor-normal pairs from a 97-patient cohort of metastatic and treatment-resistant cancers provided informative, actionable results in 91 (94 %) cases, and treatment was guided by WES results in five (5 %) of these cases [12]. A multi-institutional integrative WES and RNA-Seq of 150 metastatic, castration-resistant prostate cancer (mCRPC) has identified actionable molecular alterations in 90 % of cases with 8 % harboring germline findings [13]. Most recently, the Peds-MiOncoSeq consortium reported clinical WES and RNA-Seq of 91 pediatric refractory or relapsed cancer patient samples [14]. Actionable findings were obtained in 42 (46 %) cases, and resulted in individualized actions involving either a change of treatment or genetic counseling in 23 (25 %) cases.

Here we describe the development and clinical application of an integrative genomic approach to facilitate PCT. At the time of this writing, a total of 65 patients with malignancies were enrolled in our study. For these patients, we performed WES, targeted panel sequencing, and single-nucleotide polymorphism (SNP) microarray genotyping on tumor and patient-matched normal DNA samples, as well as RNA-Seq on tumor and adjacent normal tissue samples, when available. Genomic data analysis was integrated with cancer knowledge bases and a cancer-type-specific workflow was developed for data interpretation. Our results support the concept that WES provides a more complete spectrum of cancer genomic alterations in comparison to targeted cancer panels. WES of tumor-normal paired samples also allowed us to assess germline variants conferring increased cancer risk or having involvement in drug metabolism. Moreover, we show that RNA-Seq data provide additional clinically relevant information. As expected, the integrated genomic approach utilized in our study identified more cancer-relevant somatic mutations and more actionable alterations than several commercially available targeted cancer panels in use today. In comparison to previous prospective clinical sequencing studies using WES [11, 12], we applied comprehensive genomic profiling utilizing multiple platforms including WES, SNP microarrays, and RNA-Seq. Although both WES and RNA-Seq were used in the mCRPC study, it was not clear if written reports of genomic findings with therapeutic recommendations were returned to the patients and the physicians, and if the findings had impacted therapeutic decisions [13]. While the Peds-MiOncoSeq consortium published WES and RNA-Seq of 91 pediatric cancers and the clinical actions taken based on genomic findings [14], our report represents a large-scale prospective clinical study in adult solid-tumor cancer patients applying comprehensive genomic profiling to guide PCT and returning results to patients and physicians. In addition, this is also the first study in a prospective clinical setting where multiple platforms are utilized to identify somatic mutations (WES and targeted panel) and to detect copy number alterations (WES and SNP array) for cross-platform comparison and validation.

## Methods

### Patient enrollment

Patients were either self-referred or physician-referred. Enrollment criteria changed during the multi-year course of the study, which spanned two annual renewals of the institutional biorepository and genomic sequencing protocols approved by the Mount Sinai Institutional Review Board (IRB), but generally included: solid tumor cancer (emphasis on colorectal, breast, or medullary thyroid carcinoma) and a prognosis of 6 months of survival (amended to 12 months partway through the study). Patients were allowed regardless of geographic location within the United States or whether they received any care for their cancer at Mount Sinai. Self-reported medical history and pedigrees to assess for evidence of familial cancer were collected by the genetic counselor during the consent and enrollment process. For Mount Sinai patients, their electronic medical records (EMR, by Epic Systems) were retrospectively examined for this manuscript, but not available during generation and delivery of genomics findings. Patients were classified as “internal” (Table 1; Additional file 1: Table S1) if they had entries in the Mount Sinai EMR pertaining to their cancer care; however, this does not necessarily mean that all of their cancer care or procedures yielding sequenced specimen were conducted at Mount Sinai. As part of the IRB-approved genomics protocol, genomics findings were returned to the patient and treating physician in conjunction with a genetic counselor to provide interpretative assistance.

**Table 1:** Demographics of patient sub-cohort for whom genomics data were successfully generated

Characteristics	Number (%) of patients (*N* = 46)
Age at diagnosis^a^ (median and range, years)	48 (12–69)
Gender	
Women	26 (56.5 %)
Men	20 (43.5 %)
Race^b^	
White	18 (39.1 %)
Unknown	16 (34.8 %)
Other	5 (10.9 %)
Asian	3 (6.5 %)
Black or African American	3 (6.5 %)
American Indian or Alaska Native	1 (2.2 %)
Native Hawaiian or Other Pacific Islander	0 (0.0 %)
Cancer type	
Colorectal^c^	18 (39.1 %)
Other (single-primary)^d^	7 (15.2 %)
Breast^c^	6 (13.0 %)
Multiple primaries^e^	6 (13.0 %)
Medullary thyroid carcinoma	5 (10.9 %)
Unknown primary	4 (8.7 %)
Had metastatic disease at diagnosis^a^	
Yes	21 (45.7 %)
No	23 (50.0 %)
Unknown	2 (4.3 %)
Had metastatic disease at time of collection of sequenced tumor specimen	
Yes	28 (60.9 %)
No	16 (34.8 %)
Unknown	2 (4.3 %)
Sequenced tumor specimen type	
Primary	22 (47.8 %)
Metastatic	13 (28.2 %)
Unknown	4 (8.7 %)
Primary and metastatic	3 (6.5 %)
Lymph node	2 (4.3 %)
Primary and lymph node	1 (2.2 %)
Local recurrence	1 (2.2 %)
Patient type	
Internal (have cancer care in Mount Sinai EMR)	22 (47.8 %)
External	24 (52.2 %)

^a^For patients with multiple primaries (*N* = 6), the indicated characteristic is given for disease corresponding to the most recent primary

^b^Ethnicity information (Hispanic/Latino or non-Hispanic/Latino) was not collected

^c^The number represents patients with the indicated tumor type exclusively. Patients with multiple primaries including the indicated tumor type are not counted here, but counted in the “multiple primaries” category. One patient had two breast cancer tumors during her lifetime that were classified as independent primaries, therefore her count is given under “breast” as cancer type, although she counts as “multiple primaries” in tabulating other characteristics

^d^Other single-primary cancer types (*N* = 1 for each) are: carcinoid tumor of the midgut, glial neoplasia, malignant insulinoma, leiomyosarcoma, malignant peripheral nerve sheath tumor, pancreatic cancer, and squamous cell carcinoma of the tongue

^e^Patients with multiple primaries had these combinations: breast and non-small-cell lung cancer; breast and colon cancer; breast and follicular papillary thyroid cancer; leukemia and squamous cell carcinoma of the skin; ovarian, lung, and thyroid cancer; sarcoma and non-small-cell lung cancer

### Sample processing

Frozen or FFPE tumor specimens were obtained. For genomic DNA to serve as a patient-specific normal control, whole blood in EDTA or saliva was collected either at the time of surgery or enrollment; in some cases, genomic DNA from uninvolved adjacent tissue from tumor resection was used as the normal control. All specimens were inventoried and processed by an institutional cancer biorepository, which included pathologist assessment of tumor content by H&E staining. Only those specimens with >50 % tumor were included in the study. DNA was isolated from tissue specimens using the QIAamp DNA micro kit (Qiagen); RNA was isolated from frozen tissue only (separate spatial regions from those used for DNA) using the miRNeasy mini kit (Qiagen) for RNA. DNA extraction from blood and other sources was performed using the Maxwell 16 LEV Blood DNA kit (Promega). Both DNA and RNA quantification were determined by NanoDrop 2000 Spectrophotometer (Thermo Scientific).

### Selection of genomic assays

**Fig. 1:**
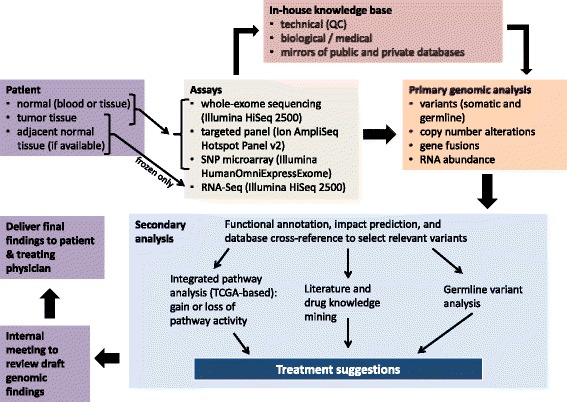
Overview of workflow

For patients P0006 through P0046 (the latter 41 out of the 46 patients enrolled in the study), a single consistent study protocol/workflow (Fig. 1) was followed, with the subset of assays to run selected using the following decision process. Genomic DNA (gDNA) samples were re-assessed for quantity by Qubit fluorometry (Life Technologies, Grand Island, NY, USA) and for quality by the 2100 Bioanalyzer or 2200 TapeStation system (Agilent, Santa Clara, CA, USA). As tumor-derived gDNA tends to be limited in quantity, the double-stranded gDNA concentration according to the Qubit assay was used to determine which gDNA assays to run using the following decision procedure (aiming to retain >500 ng of gDNA after assays for validation). If gDNA mass was <1.5 ug for either the normal or tumor specimen, only the targeted panel assay was run, as it requires the smallest amount of input DNA (10–100 ng), can tolerate poorer-quality specimens, and provides the highest sequencing depth on key somatic mutation hotspots. If gDNA mass was 1.5–2.5 ug for both normal and tumor specimens, both targeted panel and WES were run (note some patients had two tumors fitting this criteria, which were both multiplexed with the same normal control and run). For cases where 2.0–2.5 ug was available, WES library preparation was attempted up to two times (500 ng per attempt), as library prep failure sometimes occurred on first try (see Additional file 1: Table S2). If >2.5 ug was available, all assays (targeted panel, WES, and SNP microarray) were run. Patients P0001 through P0005 (the earlier 5 out of 46 patients) followed an earlier version of the study protocol which did not include arrays and targeted panel sequencing, but did include WGS for some patients (see Additional file 1: Table S2). For all patients, RNA-Seq on tumor RNA was only carried out if frozen tumor tissue was available; if frozen adjacent, uninvolved normal tissue was available, it was included in the RNA-Seq assay, but lack of adjacent normal did not exclude carrying out tumor RNA-Seq.

### Genomic assays (WES, WGS, RNA-Seq, targeted panel, SNP microarray genotyping, targeted amplicon sequencing for variant validation)

These methods are given in Supplementary Methods (Additional file 2). All Illumina microarray and next-generation sequencing procedures were carried out in the CLIA-certified Genomics Core Facility at the Icahn School of Medicine at Mount Sinai.

### Identification of genomic alterations

These methods are given in Supplementary Methods (Additional file 2).

### Tumor purity estimation

These methods are given in Supplementary Methods (Additional file 2).

### Kolmogorov-Smirnov (KS) test

The *D* test statistic and exact *p* values are computed by the function “ks.test()” from the base package “stats” of the R programming language (v3.2.1).

### Mutation nomenclature

When describing DNA single-nucleotide variants (SNVs), we use the same convention as [15, 16] where we show only the change of the pyrimidine base in a DNA base pair, e.g. the notation C > T refers to a C:G > T:A base pair transition and the notation C > G refers to a C:G > G:C base pair transversion.

### Cancer sub-classification

Breast tumors were grouped into five intrinsic subtypes based on their gene expression profiles: luminal A, luminal B, HER2 enriched, basal-like, and normal-like according to St. Gallen International Expert Consensus 2011 classification system [17]. The centroid-based Prediction Analysis of Microarray (PAM) method using PAM50 50 genes was used for the intrinsic subtype prediction [18, 19]. In addition, we also downloaded TCGA breast cancer (BRCA) RNA-Seq and metadata and normalized our breast tumor samples to BRCA data with quartile normalization method. We then performed an unsupervised hierarchical clustering analysis on the combined data using the 1000 most variably expressed genes. Each hierarchical cluster was annotated by BRCA subtype description and used to predict intrinsic subtypes in our breast cancer cases. We observed high concordance between PAM-based and unsupervised clustering based methods.

### Breast cancer germline mutation analysis

A list of 167 genes with any known association with breast cancer was compiled from public databases: VarDi [20, 21], HGMD [22], and the GWAS Catalog (http://www.ebi.ac.uk/gwas) (Additional file 1: Table S3). Any germline variants in these breast cancer associated genes for the patients in our study underwent careful manual inspection and literature review.

### Cancer signaling pathway analysis

Identification of cancer cell mutations, at the genomic level, provides a basis for reconstruction of patient specific regulatory networks underlying oncogenesis. In this study, we utilized a systems level approach for reconstruction of the receptors, signaling pathways, and effector systems within each cancer cell (Additional file 1: Table S4). This was constructed through a manual curation process involving several sources including KEGG [23, 24]. During the process, genes that are distantly related to the pathway were often not included. Genes that were altered in specific patients were crossed against this gene set (453 genes). In this manner, the complement of genomic alterations was projected onto a functional cell biology network in order to highlight underlying driver mechanisms. This was then used to identify loci that are suited for therapeutic targeting.

### Generation of summary genomic findings documents

A summary document was generated for each patient. The document was designed to maximize clinical utility in an easy-to-digest format and was developed by a team with expertise in clinical oncology, pathology, clinical genetics, cancer biology, and bioinformatics. The format and content of each document is slightly different depending on cancer types and data availability, but most of these documents included the following sections.

#### Somatic mutations

There were over 100 somatic mutations in most tumor samples in our study. A tier system was developed to associate somatic mutations with their potential clinical relevance for easier interpretation. Genes with somatic mutations were grouped into five different tiers and the mutations were reported accordingly. Somatic mutations in genes known to be involved in the patient’s specific cancer type based on internal manual curation were classified as tier 1. Somatic mutations in pan-cancer genes (i.e. genes known to be involved in multiple cancer types) but not in tier 1 were labeled as tier 2. Tier 3 are somatic mutations in genes known to play a role in other cancers based on internal manual curation, but excluding tiers 1 and 2. Tier 4 variants are somatic mutations that were previously observed in the COSMIC database [25] but where the genes were not known to be associated with any cancers. All other somatic mutations were cataloged as tier 5. Tier 1–4 somatic mutations for the 45 patients who received findings are listed in Additional file 1: Table S5. Gene function and pathway information for each tier 1–4 somatic mutation were given in the findings.

#### Prediction of drug response

Based on literature mining, biomarker database integration, manual curation, and expert opinions, a knowledge base was developed to associate genetic variants with tumor sensitivity or resistance to all FDA-approved therapies for each tumor type. In each summary document, we first separate all possible therapeutics into two categories: FDA-approved therapies for the tumor type (tier 1) and all other therapies including experimental drugs (tier 2). Based on the knowledge base, we used a decision-tree based approach to evaluate the impact of the detected genetic alteration on tier 1 drug response. Each alteration-derived clinical indication was assigned a level of evidence as: Definitive (FDA approved); Strong (NCCN guideline or major prospective clinical trial confirmed); Moderate (≥2 studies supported, including at least one clinical study); Weak (few clinical studies supported or from in vitro/animal studies or conflicting results). The decision tree was built following the order of these confidence levels. We first use genetic alterations, if any, associated with “Definitive” clinical indication to determine the potential benefit of drug response. If there are no such alterations present, we proceed to alterations associated with “Strong” level evidence, followed by “Moderate” and “Weak” levels. We follow this process to include genetic alterations linked to drug responses with evidences of all confidence levels in a hierarchical manner in the decision tree. An exemplar decision tree for anti-EGFR drugs (cetuximab, panitumumab) in colorectal cancer is illustrated in Additional file 3: Figure S1. We also curated a drug target database connecting FDA-approved or investigational therapeutics to molecular targets and then to cancer signaling pathways. Based on the drug target database and pathway analysis, we identified clinically relevant alterations and their corresponding tier 2 therapies. The combination of therapies was also recommended based on multiple alterations or feedback loops in signaling pathways. Any alterations that led to either tier 1 or tier 2 therapeutic recommendations are defined as actionable alterations.

#### Prediction of toxicity

There are many casual relationships between genetic variants and drug toxicity, some of which led to the inclusion of pharmacogenomic information for chemotherapeutics in FDA drug labels. Based on FDA labeling (http://www.fda.gov/Drugs/ScienceResearch/ResearchAreas/Pharmacogenetics/ucm083378.htm), literature mining, and expert review, we curated a pharmacogenomics knowledge base on human genetic variants associated with drug toxicity, with an objective to facilitate clinical decision-making with respect to the selection of optimal drug, dosing, and treatment duration. All tier 1 drugs were analyzed based on patient’s germline variants and genomic alterations using the knowledge base and the predicted outcomes were classified as “Severe Toxicity,” “Elevated Toxicity,” “Normal,” and “Less Toxicity”.

#### Prognosis

In addition to the prognostic value of the TNM (tumor size, nodes, metastasis) staging system, more and more molecular-based biomarkers have been reported for prognostic purposes. These prognostic biomarkers have an association with clinical outcomes such as overall survival or recurrence-free survival and are of high relevance in therapeutic decision procedures in order to individualize treatment. Similar to the predictive knowledge base development, based on literature mining and expert opinion we also curated a list of prognostic biomarkers for colorectal cancer, breast cancer, and medullary thyroid carcinoma (MTC) – three major cohorts in our study. Each sample within these cohorts would match its genomic profile to the prognostic biomarkers and the prognostic implication would be reported.

#### Clinical trial connection

A growing number of therapies that target tumors with specific genomic alterations have been developed and tested in clinical trials. By identifying clinically actionable genomic alterations in tumor for each patient and matching them with experimental drugs in clinical development, we can complement the standard of care with expanded treatment options. The inclusion/exclusion criteria and trial location and open/close date information were also downloaded from ClinicalTrials.gov and used to direct patients to most appropriate clinical trials.

### Functional validation of EGFR p.D587H mutation

These methods are given in Supplementary Methods (Additional file 2).

## Results

### Overview of the approach and workflow

We developed and implemented a workflow and supporting computational infrastructure for multi-lab, multi-assay molecular profiling of tumor specimens, generation of genomic findings and interpretations relevant to clinical decisions, and their delivery to cancer patients and their treating physicians (Fig. 1; Additional file 4: Figure S2). Briefly, normal DNA isolated from peripheral blood or uninvolved normal tissue, tumor DNA isolated from FFPE or fresh frozen tumor samples, and total RNA isolated from fresh frozen tumor and adjacent normal tissue when available, were interrogated with several genomic assays depending on the nucleic acid sample availability, quantity, and quality, choosing from: WES, targeted panel sequencing, SNP microarray genotyping, polyA-enriched or rRNA-depleted RNA-Seq. WES and targeted panel data were used for germline and somatic variant calling; a rigorous manual review process (Additional file 2: Supplementary Methods) was employed to minimize the variant calling false discovery rate from all DNA sources (frozen, FFPE, or blood), ameliorating the impact of sequencing artifacts. Somatic copy-number alteration (CNA) analysis was independently carried out using WES and array data, followed by concordance analysis. When available, RNA-Seq data were used for gene fusion detection, differential expression analysis, and to establish whether somatic SNV or indel mutations identified from WES and targeted panels were present in the transcripts. Resulting genomic data, as well as self-reported medical history and available pathology reports, were manually reviewed by a team of bioinformaticians, a cancer molecular biologist, a medical oncologist, and a genetic counselor, to produce an electronic PDF document summarizing clinically relevant findings (Additional file 5). These results included a list of relevant somatic mutations/alterations, drugs whose benefit may be altered given the patient’s somatic or germline variant makeup, a prognostic biomarker summary, clinical trial recommendations (with an emphasis on those where enrollment criteria include variants detected in the patient), and a cancer pathway perturbation summary. This document was delivered to the patient and his/her treating physician by a genetic counselor, who was also available to answer follow-up questions from the patient.

Out of the 65 patients enrolled in our study, genomic data were generated and fully analyzed on 46 patients (Table 1; Additional file 1: Table S1), with genomic findings returned to 45 of these 46 patients (one patient had several assays successfully run that produced genomic data but was notified that no clinically relevant results were found to return). The status of the remaining 19 patients is as follows: we did not initiate any genomic assays for ten patients due to insufficient tumor volume or lack of tumor specimen; four patients died before data analysis (processing was suspended for these); one patient had a tumor specimen that yielded poor quality DNA such that no attempted assay was successful; four patients are still in progress at the time of this writing.

Demographics and clinical characteristics of the sub-cohort of 46 analyzed patients are summarized in Table 1, with per-patient details described in Additional file 1: Table S1. This sub-cohort is highly heterogeneous with regards to cancer type, with a comparatively large number of patients with rare cancers and/or multiple primaries. Of the analyzed tumor samples, 44 % were from a metastasis, local recurrence, or positive lymph node; 61 % of the patients had developed metastatic disease by the time tumor samples were collected for genomic assays.

### Primary genomic analysis

For each patient, all attempted genomic assays are shown in Additional file 1: Table S2. Summary quality control (QC) statistics for WES, targeted panel sequencing, and RNA-Seq are described in the Supplementary Results (Additional file 2) and Tables S6, S7, and S8 (Additional file 1), respectively. Concordance of somatic mutations identified by WES versus those identified by targeted panel sequencing was assessed (Additional file 2: Supplementary Results; Additional file 1: Table S9). Eighty-five percent of patients had somatic SNV and indel calls in exact concordance between the two assays. All of the discordant somatic mutations were clinically relevant, had low allelic fractions (less than 0.22), and were exclusively called by the targeted panel pipeline but not by the WES pipeline, suggesting that the targeted panel is more sensitive than WES in the targeted regions, likely due to the increased sequencing depth (median 2276X normal and 1994X tumor sequencing depth for panel versus median 66X normal and 112X tumor sequencing depth for WES). We also performed comparative analyses of CNA derived from WES and SNP arrays and demonstrated overall concordance between the two platforms for CNA detection (Additional file 2: Supplementary Results; Additional file 6: Figure S3A, Figure S3B; Additional file 7: Figure S4; Additional file 8: Figure S5; Additional file 9: Figure S6).

**Fig. 2:**
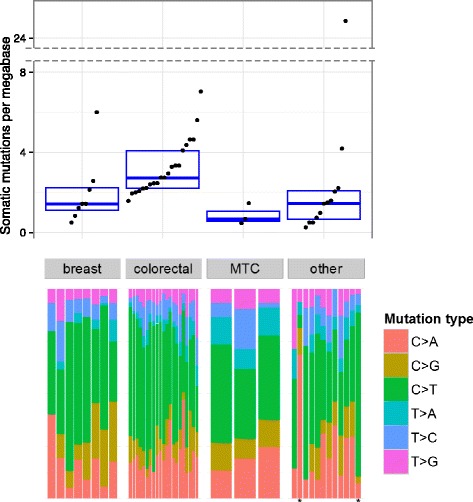
Somatic mutation frequencies in 40 patients having WES data, grouped by cancer type: breast, colorectal, medullary thyroid carcinoma (MTC), and other. Each *dot* represents a tumor-normal sample pair from a patient; patients with multiple tumors are shown as multiple points, one per tumor. The *bottom panel* shows the distribution of six possible base pair substitutions in each tumor (see “Methods” for mutation nomenclature), ordered to correspond with frequency data points. Only non-synonymous SNVs and SNVs altering the canonical splice sites are counted and only if this functional impact is in a canonical protein isoform of the gene. Frequencies were obtained by dividing these mutation counts by the genomic area in coding exons in WES-targeted regions. Patient P0003 was omitted because the purity of WES-sequenced tumor was <5 % based on the allelic fraction distribution of somatic mutations (Additional file 2: Supplementary Results)

We analyzed the spectrum of mutation frequencies and types in our cohort (Fig. 2). Even with a relatively small cohort size, heterogeneity between tumor types and within tumor types were observed. The heterogeneity is reflected by both widely varying mutation frequencies (Fig. 2, top panel) and, to a lesser extent, different mutation signatures (Fig. 2, bottom panel). Median frequencies of 1.4 mutations/Mb for breast cancer and 2.7 mutations/Mb for colorectal cancer are consistent with previously published NGS studies [15, 16], and no hypermutated colorectal cancers were observed. Medullary thyroid carcinoma is among the least mutated cancers in the cohort (median 0.7 mutation/Mb), consistent with previously found low mutation frequencies in thyroid cancers in general [15, 16, 26]. Similarly, we detected a high proportion of C > T mutations (see “Methods” for mutation nomenclature), consistent with what has been published on the same tumor types as we analyzed in our study [15, 16]. Two specimens stand out with respect to their mutation profile: a cancer of unknown primary origin (patient P0017, favoring germ cell tumor) having a low overall mutation frequency, but an unusually high fraction of C > A transversions, and a squamous cell carcinoma (SCC) of the skin (patient P0011) having an unusually high overall mutation frequency dominated by C > T transitions (Fig. 2). The latter is a patient whose SCC followed an alemtuzumab treatment to combat graft-versus-host disease (GVHD); the somatic mutation spectrum may indicate the mutagenic process that led to the development of SCC (see Additional file 2: Supplementary Results; Additional file 10: Figure S7; Additional file 1: Table S10). Somatic mutations were also categorized based on their cancer relevance (see “Methods”). Mean 17.3 cancer-relevant somatic mutations per patient were identified and returned in the genomic findings document (Additional file 1: Table S5).

Somatic CNAs affect a greater portion of cancer genomes than SNVs and play a critical role in activating oncogenes or inactivating tumor suppressors [27]. A major challenge in CNA analysis is to differentiate driver CNAs that contribute to oncogenesis and cancer progression from those passenger CNAs that are acquired during cancer development but do not have functional consequences. Common criteria for driver CNA prediction include amplicon size and association of gene expression with copy number alterations. In order to determine potential oncogenic driver CNA events with high confidence, we examined the relationship between CNAs and gene expression based on RNA-Seq data. For example, we reported amplification of CCND1 in two breast cancers and the CNA calls were supported by CCND1 gene expression analysis (Additional file 11: Figure S8).

Gene fusions represent a key oncogenic event in many cancer types [28]. We implemented a comprehensive computational pipeline incorporating four fusion callers (see “Methods”) to detect gene fusions from RNA-Seq data. Putative gene fusion events have been identified in 14 tumors and one fusion has been validated by PCR via breakpoint mapping (Additional file 2: Supplementary Results; Additional file 12: Figure S9A, Figure S9B). However, for the cohort in this study, none of the predicted fusions were considered actionable and thus not included in the findings returned to the patients and physicians.

### Identification of clinically actionable alterations through integrative genomic analysis

The primary goal of our integrative approach was to utilize multi-platform genomic profiling data to generate, at the cellular level, molecular portraits of the oncogenic signaling networks underlying these cancers. Recommendation of appropriate targeted therapeutics would follow through execution of a manual review process performed in a case-by-case manner. Any alterations that had clinical implications in either tier 1 or tier 2 therapeutics (see “Methods”) were defined as actionable alterations. For clinical trial connection, inclusion/exclusion criteria, trial location and open/close date information were downloaded from ClinicalTrials.gov and used to direct patients to the most appropriate clinical trials. We also contacted PIs for specific trials for clarification of inclusion and exclusion criteria when the information from ClinicalTrials.gov lacked sufficient details.

Five patients with MTC were analyzed in this study. It has been well recognized that the RET oncogene is mutated in most MTC cases either in the germline or by somatic mutation [29, 30]. All five of the cancers assayed in this study had activating mutations in RET (Table 2). RET kinase inhibitors vandetanib and cabozantinib were recommended for these patients [31]. In one case (patient P0010), additional alterations such as CDKN2 and RASA1/3 deletion were identified and this evidence suggested recommendation of clinical trials utilizing CDK inhibitors. The RET mutations identified in all of the MTC samples enhanced confidence in our approach to detect expected mutations within a given cancer type.

**Table 2:** Summary of genetic alterations in five cases of medullary thyroid carcinoma (MTC)

Patient ID	RET mutation	Other alteration
P0010	p.C634R (germline)	Somatic CNA (deletion of CDKN2, RASA1/3, RB1)
P0029	p.C634Y (germline)	
P0036	p.M918T (somatic)	
P0041	p.M918T (somatic)	
P0044	p.M918T (somatic)	

**Fig. 3:**
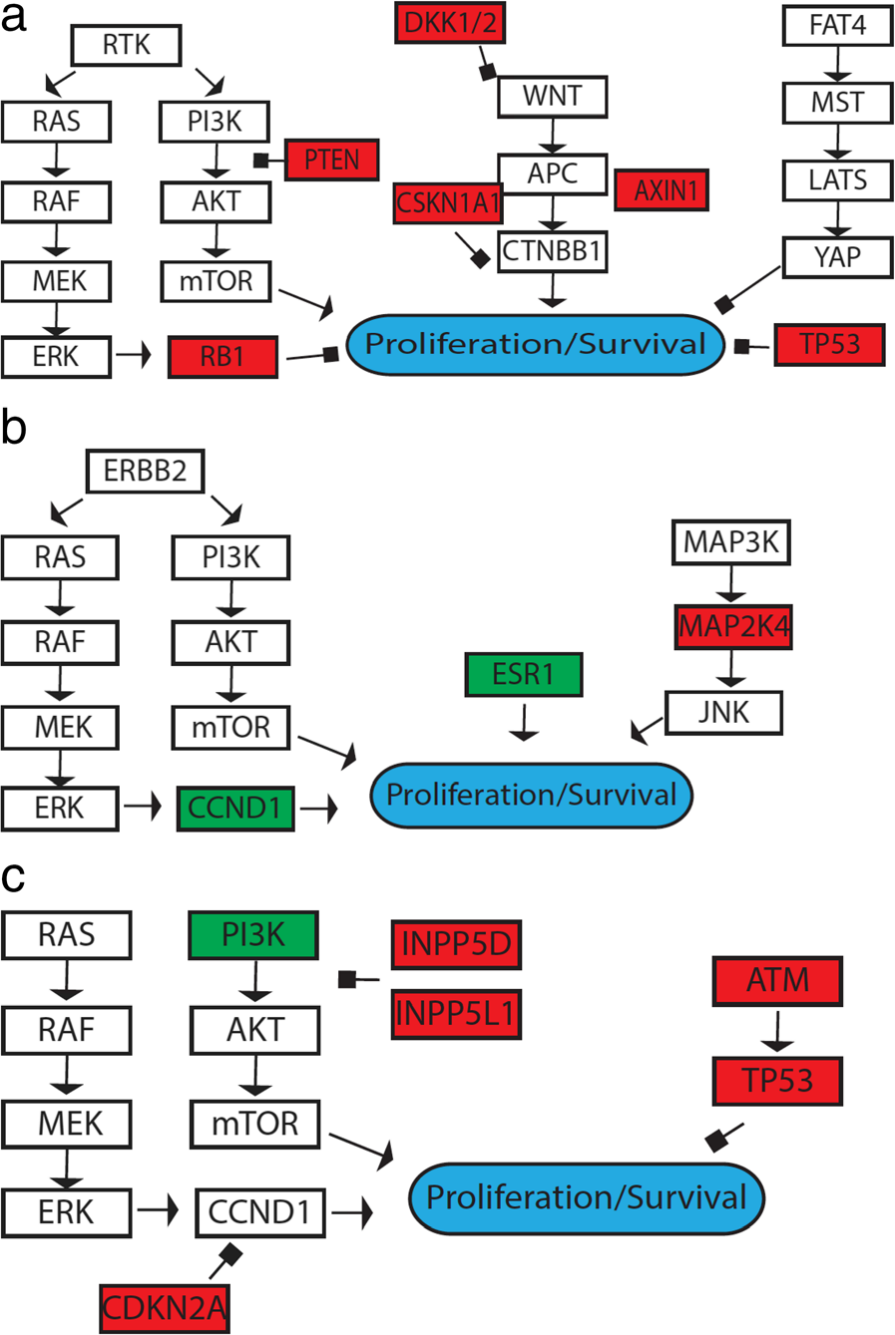
Multiple somatic alterations in components within the same pathways. **a** Multiple somatic alterations within the APC pathway observed in a colorectal cancer. A schematic of the signaling pathways converging on growth control of colorectal cancer patient P0027 is displayed where mutation and predicted loss of function of tumor suppressors is depicted in *red*. Several components excluding APC are mutated in the canonical WNT signaling pathway. **b** Identification of an oncogenic driver in a breast cancer. A schematic of the signaling pathways converging on growth control of breast cancer patient P0040 is displayed where mutation and predicted loss of function of tumor suppressors is depicted in *red* and activation of oncogenes is depicted in *green*. Amplification and overexpression of CCND1 is identified through the integrative approach utilized in this study. **c** An integrative approach identifies the PI3K pathway as potential drug target in a squamous cell carcinoma. A schematic of the signaling pathways converging on growth control of skin squamous cell carcinoma patient P0011 is displayed where mutation and predicted loss of function of tumor suppressors is depicted in *red* and activation of oncogenes is depicted in *green*. Multiple tumor suppressors in the PI3K-AKT pathway have mutations that predict loss of function (INPP5D and INPPL1). Additionally, PI3K is mutated, suggesting PI3K-AKT pathway as a possible drug target

Colon cancer was the most highly represented cancer type within the cohort (19/46 patients). The frequencies of KRAS, NRAS, BRAF, and PIK3CA mutations within these tumors (Table 3) were similar to previous studies [32]. Quadruple negative colon cancers were also observed in this cohort. Mutation of the WNT-pathway component APC was observed in 17 of the 19 colon cancer patients. One tumor (patient P0027) had a complement of mutations in non-APC components (DKK1/2, CSKN1A1, and AXIN1) of the WNT pathway (Fig. 3a). Although there are no FDA-approved drugs targeting downstream components of the APC pathway, this may enable therapeutic recommendations in the future. Another interesting finding was a higher frequency of TP53 mutations (16/19 patients, 84 %) than has been observed previously [32]. This could be explained by the high percentage of patients in our cohort who developed metastatic disease, given the role of TP53 in promoting metastasis in multiple cancer types [33].

**Table 3:** Summary of genetic alterations in 19 cases of colorectal cancer. Somatic mutations or CNA for the listed genes are shown. Blank indicates wild-type

Patient ID	APC	KRAS	NRAS	BRAF	PIK3CA	PTEN	EGFR	TP53
P0004	p.E763*	p.G12V			p.E545K, p.M1043I			
P0005	p.R554*	p.G12S						p.R273H
P0008				p.V600E				
P0009	p.E1309fs*4						Gain	p.P151S
P0016		p.G12V			p.E545K			p.R248Q
P0018	p.E1309*, p.V1377fs	p.G13D						p.G245S
P0019	p.R232*, p.R1114*							p.R333fs*12
P0020	p.T683P, p.R876*, p.E1577*	p.G13D						p.F270I
P0022	p.T1493fs*14							p.R248Q
P0024	p.E955*	p.N116H,p.Q61P						p.S183*
P0025	p.I606fs, p.R1450*		p.Q61R					p.R273C
P0027						Possible loss		Mutation in donor splice site
P0028	Splice site donor, p.Q1067*							p.R282W
P0031	p.E1097*, p.E1397*	p.G12D						p.G245S
P0033	p.E1306*	p.G13D						p.R273C
P0034	p.E1322*	p.G12C						p.Y220C
P0037	p.R232*			p.V600E		Splice site acceptor		
P0043	p.F1354fs, p.S1400*	p.G13D						p.C176F
P0046	p.R876*		p.G12D					p.S127F
Frequency	0.89	0.53	0.11	0.11	0.11	0.11	0.05	0.84

The most straightforward utility of the colon cancer data was for predicting insensitivity to anti-EGFR antibodies [34, 35]. Additionally, selective targeting of the ERK pathway through inhibition of either BRAF or MEK was better informed by the data. In the case of patient P0027, the PI3K pathway was hypothesized to be activated in the absence of ERK pathway activation. PI3K pathway activation was predicted because of the observed loss of PTEN. Therefore, our approach allowed for consideration of an additional targeted strategy utilizing AKT/mTOR inhibition (Fig. 3a). PI3K/AKT/mTOR inhibitors could also benefit patients P0004 and P0016 due the presence of PIK3CA activating mutation p.E545K in these two tumors. In patient P0046, a potential ALK activating mutation p.A1200V [36] was detected, suggesting consideration of ALK inhibitors such as crizotinib. In addition, FLT3 was also amplified in this case, supported by RNA-Seq-derived gene expression data. Therefore, the patient may also benefit from potent FLT3 inhibitors including ponatinib, cabozantinib, and quizartinib. In a quadruple negative colon cancer case (patient P0009), expression of the EGFR ligands epiregulin and amphiregulin were elevated by a remarkable 113-fold and 29-fold, respectively, in tumor samples in comparison to the adjacent normal tissue control, therefore predicting favorable outcome in response to cetuximab treatment [34, 35].

Seven breast cancer patients were analyzed in this study (Table 4). There were three estrogen receptor positive (ER+) breast cancers with mutations in oncogenes that are associated with ER+ luminal breast cancers. One case (patient P0006) harbored an activating mutation in PIK3CA (p.E542K), a well-established activator of the AKT signaling pathway. In a second case (patient P0040), CCND1 amplification was identified through the CNA analysis (Fig. 3b). In another case (patient P0002), PIK3CA activating mutation (p.E545K) as well as amplifications of CCND1 and FGFR1 were detected (Additional file 1: Table S5; Additional file 11: Figure S8). In each of these cases, alteration-specific clinical trials were suggested for targeting the underlying oncogenic drivers (PI3K/AKT pathway, CDK4/6, or FGFR1).

**Table 4:** Summary of genetic alterations in seven cases of breast cancer

Patient ID	RNA-Seq	ER mRNA	HER2 mRNA	PIK3CA	CCND1	TP53	MAP3K1	MAP2K4	PTEN	AKT1	AKT3	Other
P0002	Available	High	Low	p.E545K	Amplified		p.F1462V					
P0006	Available	High	Low	p.E542K								
P0007	Not performed	NA	NA			p.R213fs						CDK1 amplification, RASA1 loss
P0013	Available	Low	Low			p.R110P						
P0030	Not performed	NA	NA			p.Y220C						NRAS amplification
P0040	Available	High	Low		Amplified			p.W95*				
P0042	Not performed	NA	NA			p.L194R						NF1 mutation

Somatic mutations or CNA for the listed genes are shown. Blank indicates wild-type. ER and HER2 mRNA level derived from RNA-Seq data are summarized as high or low using the TCGA breast cancer RNA-Seq data as references

Three triple-negative breast cancers were represented in our cohort; this breast cancer subtype presents significant challenges with respect to aggressiveness and insensitivity to chemotherapy [37]. Currently, there are no approved targeted drugs for triple-negative breast cancer. As expected, TP53 was mutated in a high percentage of these breast cancers (Table 4) [37]. Several notable oncogenic mechanisms were observed. In one case (patient P0007), loss of RASA1, a RAS GTPase, and amplification of CDK1, a component of the CyclinB-CDK1 complex for mitotic entry, was observed, and led to the recommendation of clinical trials of CDK1 inhibitors. In another case (patient P0030), an activating mutation in NRAS was identified; activating mutations in NRAS are uncommon in triple negative breast cancer [38]. This allowed for suggestion of a MEK inhibitor. In the third case (patient P0042), a truncating mutation in NF1, a RAS GTPase, was found, therefore directing the recommendation of MEK inhibitors such as trametinib. Previous studies have indicated that NF1 mutations were observed in triple negative breast cancers [39].

Breast cancer sub-classification based on gene expression profiles has been well established and five subclasses have been defined: luminal A, luminal B, HER2 enriched, basal-like, and normal-like. Three of the seven breast tumors were ER+/PR+/HER2– based on an immunohistochemical (IHC) test in the pathology reports. Using RNA-Seq-derived gene expression data, we classified two of these three cases as luminal A and one as luminal B, indicating gene expression based classification could be used to confirm the pathology report. In addition to prognostic differences between luminal A and luminal B [40, 41], patients with luminal A type are generally more responsive to hormonal therapy and less responsive to chemotherapy than patients with luminal B type [42]. Therefore, gene expression-based classification may improve prognostic assessment and provide additional information to guide treatment selection compared to routine pathologic classification. Interestingly, we classified one case (patient P0013) as basal-like but the pathology report labeled the case as ER+/PR-/HER2– based on IHC, though only 10 % of the tumor nuclei stained positive for ER and ER staining was weak (1+). The tumor was classified as ER+ in the pathology report based on current ASCO/CAP guidelines [43]. However, the majority of the tumor cells are triple-negative, most likely explaining the classification results derived from gene expression data.

Germline variants in genes associated with breast cancer risk were identified in breast cancer patients in this study. A panel of established and putative breast cancer risk genes was compiled and this was cross-referenced against the list of germline variants (see “Methods”). Approximately ten germline variants per patient were identified within these genes. Notably, a DCLRE1C p.S635_L636fs mutation was identified in a patient (P0040) diagnosed with breast cancer at the age of 23 years. DCLRE1C encodes a nuclear endonuclease that is required for functional repair of DNA double-stranded breaks [44]. This mutation would not have been identified with existing targeted panels. In patient P0013, a germline mutation in BRCA1 (p.W1712fs) was identified that confer increased breast cancer susceptibility. Cisplatin was recommended for this patient, as breast cancers harboring germline BRCA1 mutations are highly sensitive to cisplatin chemotherapy [45, 46].

Several cancer types represented by a single patient were also analyzed; 14 patients received findings. Analysis of a patient (P0045) with three independent primary tumors (non-small cell lung cancer, ovarian cancer, papillary thyroid cancer) arising over the span of 14 years revealed distinct driver oncogenes in different tumors. The NSCLC tumor harbored an activating mutation of PIK3CA (p.E545K) and the papillary thyroid tumor harbored an activating mutation in BRAF (p.V600E); neither mutation was detected in the ovarian cancer tumor despite high-depth, sensitive targeted panel sequencing. A patient (P0011) with squamous cell carcinoma of the skin was also analyzed. Several alterations of genes (PIK3R1 p.E443K, INPP5D p.S19C, INPPL1 p.R346W, CDKN2A p.V82E) encoding proteins involved in the AKT pathway were found (Fig. 3c). Although no mutations in well-characterized recurrently mutated oncogenes or tumor suppressors of the AKT pathway were found, collectively these alterations allowed for the prediction of AKT pathway inhibitor sensitivity (Fig. 3c). Additionally, this patient had an extraordinarily high mutational load (Fig. 2), predicting clinical response to immunotherapy [47]. A 12-year-old girl (patient P0038) was diagnosed with glial neoplasia with high grade of malignancy and aspects of melanocytic differentiation. The BRAF p.V600E mutation was detected in the tumor specimen and we recommended a BRAF inhibitor based on limited but encouraging clinical evidence [48]. Patient P0032 had a malignant peripheral nerve sheath tumor (MPNST). Analysis of germline variants revealed a loss of function mutation in NF1 (p.R2637fs). NF1 inactivation is a hallmark in MPNST accounting for approximately half of the cases. Several pre-clinical studies have indicated that MEK inhibitors or mTOR inhibitors exhibited efficacy in MPNSTs harboring NF1 loss of function mutations, demonstrated in in vitro, in vivo, and in human MPNST ex vivo experiments [49–51]. Presence of NF1 germline mutation suggests the patient may benefit from anti-MEK or anti-mTOR agents that are commercially available for other cancer indications.

Three patients had tumors of unknown origin. Notably, two of these cases had NF2 loss of function mutations. In both of these cases, renal cell carcinoma, a cancer with a relatively high frequency of NF2 mutation [52], was high-ranked on the list of considered diagnoses based on standard pathologic assessment. This suggests that cancer genomic sequencing may be useful for assistance with diagnostics. In one of these three possible renal cell carcinomas (case P0039), copy loss of TSC1 and a likely loss-of-function deletion in NF2 were detected, strongly suggesting AKT/mTOR pathway was activated. This led to the recommendation of mTOR inhibitors for the patient, as loss of TSC1/2 through mutations or deletions have been associated with everolimus response in multiple cancer types including bladder cancer [53], hepatocellular carcinoma [54], subependymal giant cell astrocytoma (SEGA), and renal angiomyolipoma [55].

**Fig. 4:**
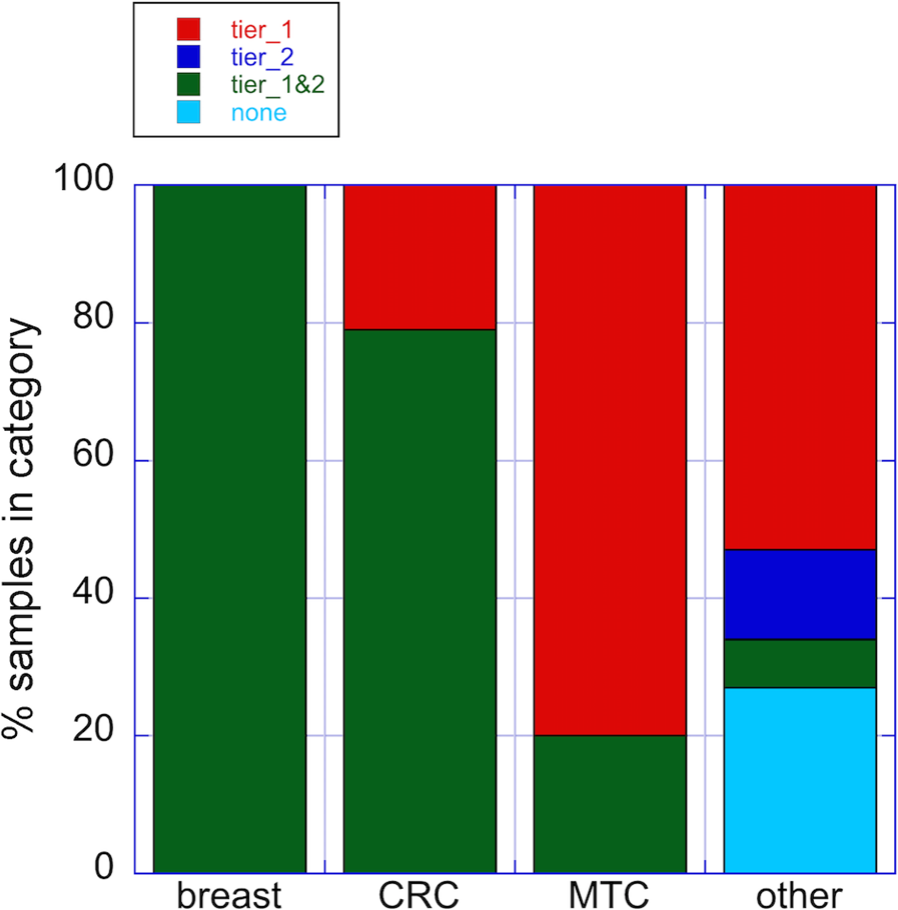
Actionability across multiple cancer types in this study. A summary of the distribution of recommendations across cancer types, where tier 1 and tier 2 drugs (see “Methods” for definitions) is displayed. "CRC" is colorectal cancer

In summary, we made therapeutic recommendations for 42 of the 46 patients (91 %) based on their profiles of genetic and genomic alterations (Fig. 4). Full somatic mutation call results for 25 patients who consented to public data release are provided as VCF files (Additional file 13). Our results are consistent with previously published retrospective studies of adult cancer patients with solid tumors where actionable mutations were identified in approximately 80–90 % of the patient populations [6, 9, 11]. Not surprisingly, the four patients in our cohort without actionable alterations had a mean of only 2.5 cancer-related somatic mutations (sequencing depth was not the explanatory variable). Two of these four patients did not have RNA-Seq data due to the lack of frozen tissue samples.

### A case study

**Fig. 5:**
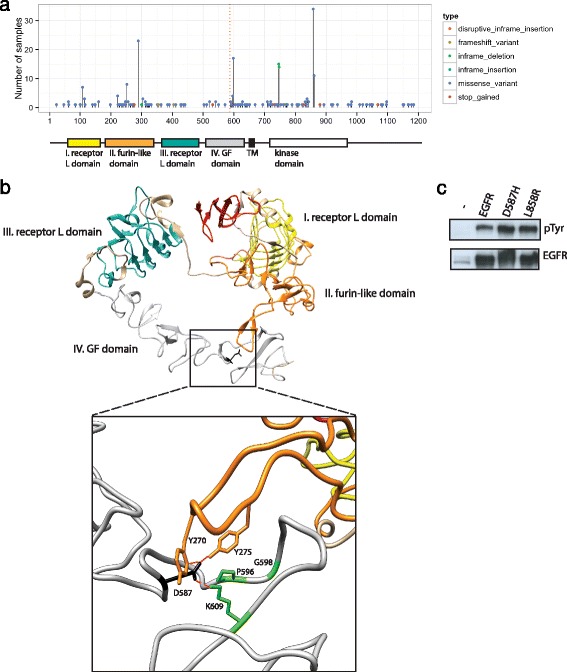
Presentation of a case study with a novel actionable mutation p.D587H (hg19 chr7:55233009G > C) in EGFR. **a** EGFR mutation frequencies in several cancer types were obtained from TCGA data (http://cancergenome.nih.gov) and plotted across the EGFR protein sequence. D587 (*dashed red line*) is located near a hotspot at G598 within domain IV. Kinase domain and domain II hotspots are also depicted. Domain structure is from Pfam [63]. **b** Structure of the extracellular region of EGFR depicting individual domains; I (*yellow*), II (*orange*), III (*teal*), and IV (*silver*). A view of the interaction between domains II and IV is illustrated (*box*). Side chain of D587 (*black*) and K609 (*green*) form an interaction (*red dashed line*). Hydrogen bonding (*red dashed lines*) between domain IV residues and domain II tyrosine residues (*orange*) stabilize the inactive conformation. Hotspot regions (*green side chains*) may be allowing for a conformation of the loop that permits interaction between D587 and K609. **c** HEK293 cells were transfected with EGFR, p.D587H, or p.L858R, and activity of EGFR was assayed by western blot using an anti-phosphotyrosine antibody to measure autophosphorylation

Of the 46 cases analyzed in our study, one case was of particular interest given the potential for significant clinical impact. Patient P0015 was diagnosed with cancer of unknown primary at the age of 55 years. We carried out genomic analysis of a metastatic liver tumor, which was classified as poorly differentiated adenocarcinoma with signet ring features. The patient had undergone radiation and chemotherapy regimens and had been recently treated with vinorelbine. Our genomic data did not show any previously known somatic mutations with available targeted therapeutic agents. However, we found a somatic mutation in EGFR (p.D587H, hg19 chr7:55233009G > C) that had never been observed in any public cancer genome sequencing databases (at the time of this report). Analysis of TCGA data shows that this mutation is close to hotspots located at P596 and G598 (Fig. 5a).

We subsequently validated this mutation by Sanger sequencing (Additional file 14: Figure S10). As a receptor tyrosine kinase, EGFR is activated through binding to ligands via its extracellular domain I and III. EGFR auto-inhibition takes place when non-covalent interactions between extracellular domain II and IV pull apart domains I and III, thus interfering with ligand binding [56]. D587 is located in extracellular domain IV and plays a critical role in auto-inhibition. It has been shown that D587 forms an interaction with K609 and that this stabilizes interactions between tyrosines 270 and 275 of domain II in the inactive structure with EGF bound at low affinity [56] (Fig. 5b). The P596 and G598 hotspot region is located within the loop stabilizing the interaction between D587 and K609, thus identifying a possible explanation for their contribution to the inactive state. Based on these findings, we predicted that p.D587H would disrupt the auto-inhibitory structure, thereby promoting ligand binding and pathway activation.

To test the functional consequences of the p.D587H mutation, wild-type EGFR, EGFR p.D587H, and EGFR p.L858R expression vectors were transfected into HEK293 cells. As shown in Fig. 5c, similar to the known activating p.L858R mutant, EGFR auto-phosphorylation is augmented by p.D587H in comparison to the wild-type protein, strongly suggesting that p.D587H activates EGFR and the activated signaling could be inhibited by EGFR inhibitors. Given this strong supporting functional genomics data, we suggested benefit from targeted anti-EGFR therapies.

### Comparative analysis of integrative genomics and cancer panels

Currently, panel-based targeted sequencing of cancer-related genes and mutational hotspots is commonly used in NGS-based clinical testing. To evaluate if the integrative genomic profiling approach employed in this study provides additional clinically relevant information, we performed a comparative analysis of cancer-related somatic mutations as well as clinically actionable alterations identified in our study versus those that would have been identified by several commercially available cancer panels (Table 5).

**Table 5:** Comparative analysis of integrative genomic approach and cancer panels. The numbers corresponding to the three cancer panels are hypothetical (based on the panel design) and not based on experimental results

Genomic approach	Mean number of cancer-relevant somatic mutations (range)	Number of patients with tier 1 drug recommendations	Number of patients with tier 2 drug recommendations	Number of patients with actionable alterations	Mean number of actionable alterations (range)
Ion AmpliSeq Cancer Hotspot Panel v2	1.3 (0–4)	24 (52 %)	16 (35 %)	24 (52 %)	0.65 (0–3)
Oncomine Comprehensive Panel	2.5 (0–11)	39 (85 %)	24 (52 %)	41 (89 %)	2.4 (0–6)
FoundationOne	3.7 (0–22)	39 (85 %)	24 (52 %)	41 (89 %)	2.6 (0–7)
This study	17.3 (1–79)	40 (87 %)	26 (57 %)	42 (91 %)	4.9 (0–14)

We first examined the number of somatic mutations in each patient. Since many of the genes with somatic mutations identified by WES have unknown functional relevance in cancer development, we only focused on cancer-relevant mutations (tier 1–4 mutations, see “Methods”). Mean 17.3 cancer-relevant somatic mutations per patient were identified in the 46 patients reported in this study, and only 1.3, 2.5, and 3.7 of these mutations would have been found by the Ion AmpliSeq Cancer Hotspot Panel v2, the Oncomine Comprehensive Panel, and FoundationOne, respectively. This represents a 13.3-fold, 6.9-fold, and 4.7-fold increase by our approach over the three panels, respectively. Next, we investigated how many of the actionable alterations we identified could have been discovered by other approaches. As shown in Table 5, in 40 of the 46 patients (87 %), alterations associated with tier 1 level drug response (defined in “Methods”) were identified by our approach, and in 24 (52 %), 39 (85 %), and 39 (85 %) patients by the other three respective targeted panels. If we took alterations associated with tier 2 drug response (defined in “Methods”) into consideration, the total number of patients with actionable alterations increases to 42 (91 %) by our study, and 24 (52 %), 41 (89 %), and 41 (89 %) by the three other respective panels.

We note that in many cases, although both panel-based approaches and our integrative genomic approach would have found a well-recognized actionable mutation, e.g. activating mutations in KRAS in colorectal cancers that are associated with lack of benefit to cetuximab or panitumumab, the comprehensive genomic profiling we applied led to the identification of additional actionable alterations in these cases that may inform alternative therapeutic options. Therefore, we further asked how many genes with genetic and genomic alterations are considered actionable (see “Methods”) in each patient. Our analysis revealed that mean 4.9 actionable alterations due to somatic mutations, CNA, germline variants, or gene expression alterations were discovered by our integrative genomic profiling, in comparison to mean 0.65, 2.4, 2.6 actionable alterations that could have been identified by the three other respective panels, representing a 7.5-fold, 2.0-fold, and 1.9-fold increase, respectively. Of the mean 4.9 alterations, 1.5 were somatic mutations, 0.6 were CNAs, 2.2 were germline variants, and 0.7 were gene expression alterations, indicating that a significant part of the actionable alterations was derived from germline data.

### Follow-up patient survey

An IRB-approved follow-up patient survey was conducted after the completion of our main study. A total of 40 patients who were not known to be deceased were contacted, of which nine were found to be deceased via the follow-up study. Ten of the 31 remaining patients (32 %) consented and completed the survey. Survey questions covered a variety of topics including overall satisfactions with the study, understanding of the research findings, and if any of the findings influenced clinical management. Seven of nine patients (78 %; one chose not to respond) stated the genomic study findings met their expectations. However, nine patients expressed some level of difficulty in understanding the research findings. Nine patients (not overlapping) discussed the results with their treating physicians. Six out of nine patients (67 %; one chose not to respond) stated that the findings were somewhat useful or very useful. Between responders of this survey (*N* = 3) and non-survey follow-up with a physician (*N* = 1), the course of treatment was altered for four patients in our study based on our genomic findings.

Although details on treatment alterations were not available from the survey, two of the four cases are colorectal cancers harboring well-known activating mutations in KRAS (p.G13D) and BRAF (p.V600E) and we suspect that the patients decided not to pursue cetuximab/panitumumab treatment and may have enrolled in clinical trials on MEK inhibitors. The third case (P0025) is also a metastatic colorectal cancer with an activating mutation in NRAS (p.Q61R), predicting insensitivity to cetuximab/panitumumab. In addition, this patient harbors germline mutations in KDR and CXCR2 associated with increased benefit to bevacizumab and germline variants in ERCC1, ERCC2, ERCC5, and XRCC1 associated with decreased benefit to oxaliplatin. Through personal communication, the patient revealed to us that indeed treatment with bevacizumab and 5-fluorouracil resulted in a brisk response that allowed for cryoablation of remaining oligometastatic lung disease, while the initial platinum-based regimen (oxaliplatin-containing) had limited efficacy. This patient remains in complete remission for 16 months at the time of this writing. The germline mutations in this patient associated with increased benefit to bevacizumab or decreased benefit to oxaliplatin would not have been identified with cancer panel sequencing on tumor samples only. The fourth case (P0015) is cancer of unknown origin with a rare activating EGFR mutation p.D587H, as described above. Communication with the treating oncologist through a genetic counselor indicated that EGFR inhibitors were considered due to the identification of the EGFR mutation. This novel somatic EGFR mutation would not have been called somatic with high confidence if tumor-only sequencing were performed using cancer panels.

## Discussion

Here we report clinical application of an integrative genomic approach for PCT. Overall, the integrative genomic approach applied in our study identified significantly more cancer-related somatic mutations as well as more actionable genetic and genomic alterations than would have been identified using any one of several commonly used commercial cancer panels (Table 5). In several patients where panel sequencing of mutational hotspots would not have identified any clinically actionable mutations, rare somatic mutations uncovered through our comprehensive genomic sequencing have the potential to impact treatment options and ultimately change clinical outcome for individual patients. Indeed, treatment decisions were influenced by our genomic studies in four cases. Other cases have been reported in which whole genome sequencing uncovered rare mutations that were missed by targeted sequencing and that had the potential to guide treatment but was discovered too late. For example, a rare activating BRAF mutation (p.L597R) was identified in an aggressive metastatic melanoma using whole genome sequencing that had previously been designated as “wild-type” for BRAF p.V600E/K mutations and several common KIT mutations using targeted sequencing [57]. The BRAF p.L597R mutation was demonstrated to be responsive to MEK inhibitors in metastatic melanomas [57], suggesting that with more comprehensive testing the patient in question may have benefitted from this treatment option [58]. While we identified actionable alterations in 91 % of the cases tested, we did not identify actionable results in four cases. More comprehensive approaches for profiling tumors that employ additional platforms such as proteomic and metabolic profiling may potentially provide clinically actionable information in these cases.

In comparison to commonly used targeted cancer panel sequencing, more comprehensive genomic profiling provides a number of advantages. First, cancer panels are generally designed to include well-characterized cancer-associated genes, whereas WES and RNA-Seq enable elucidation of the underlying cancer genetic drivers at the pathway level, given alterations in several components of the same pathway allow us to predict pathway dysregulation with higher confidence. For example, several genes such as DKK1/2, CSNK1A1, INPP5D, and INPPL1 in the cases we discussed in detail (Fig. 3) are not present on the cancer panels we examined, yet their functional roles in respective signaling pathways are well-documented [59, 60]. Many somatic mutations identified from WES are of unknown significance; however, as more WES data accumulate, retrospective analysis of these genomic data and association with clinical outcome and treatment response could inform novel actionable mutations. Second, whereas most clinically available cancer panel sequencing tests are designed to screen only tumor DNA, the more integrative profiling makes it possible to differentiate germline from somatic variants, especially in cases of novel or rare variants. Without knowledge of germline variants, accurately identifying somatic mutations becomes problematic as panel sizes increase [8]. For example, rare or private germline variants located in protein domains with hotspot oncogenic mutations, such as kinase domains, may be interpreted as somatic mutations by workflows using only tumor samples. However, knowledge that a variant is germline would be important because genetic counseling or increased surveillance may be indicated for a patient who is a carrier of a pathogenic allele, thus the cancer risk of the patient or their family may be substantially increased. For these reasons, despite the logistical challenges relating to sample collection and tissue banking, we favor sequencing of matched tumor-normal pairs. Sequencing of germline DNA provides the added benefit of identifying variants that may inform on drug metabolism or DNA repair pathways that are associated with response to chemotherapy, providing for the possibility of informing on the efficacy and toxicity of a given drug for a given individual. Such variants may be missed or inaccurately called from the tumor DNA. As illustrated in our follow-up patient survey, germline mutations associated with drug responses in patient P0025 would not have been identified with cancer panel sequencing of tumor samples only, and the rare EGFR somatic mutation in patient P0015 would not have been called somatic with high confidence without sequencing of both tumor and the matched normal control samples. In addition, germline variants that predispose to cancer may provide prognostic value and inform on treatment options, as we demonstrated in one of our cases (P0013) with the identification of a BRCA1 germline mutation that led to our recommendation for cisplatin chemotherapy.

We have further shown that RNA-Seq can significantly augment the utility of genetic testing for PCT. Across a number of cancer types, clinically relevant subclasses can be defined based on gene expression patterns. In breast cancers, both luminal A and luminal B subclasses are ER+. However, luminal A subtype is more responsive to hormonal therapy while luminal B subtype is more responsive to chemotherapy [42]. Therefore, sub-classification of breast cancers using RNA-Seq-derived signatures may have clear therapeutic implications. For example, in one of our cases (P0013), we identified a discrepancy in classification of the breast cancer in this patient between the pathology report (which classified the patient’s tumor as ER+ under the current ASCO/CAP guidelines [43]) and the RNA-Seq analysis results we carried out on this patient (resulting in a classification of basal like). A re-review of the pathology indicated that only 10 % of the tumor nuclei stained positive for ER, whereas the majority of the tumor cells are triple negative ER–/PR–/HER2–, suggesting in some cases the molecular profiling data may lead to a more accurate molecular characterization of the tumor. It is not uncommon that cancer driver pathways are activated by abnormal expression of key pathway components in the absence of genetic alterations. For example, high levels of expression of epiregulin and amphiregulin not only imply EGFR pathway activation, they have also demonstrated clinical utility as predictive biomarkers for response to anti-EGFR treatment [34, 35]. In patient P0009, a quadruple negative colon cancer case, both epiregulin and amphiregulin exhibited extraordinary over-expression based on RNA-Seq data analysis, making a case for cetuximab treatment. Identification of these types of gene expression biomarkers in the absence of genetic alterations would not be possible with DNA sequencing data alone. In addition, RNA-Seq data can be used to confirm somatic mutations identified in DNA or to infer driver CNAs when gene expression correlates with copy number changes. In genomics findings documents we generated, somatic mutations detected by both WES and RNA-Seq were denoted as “validated” to emphasize their significance. Finally, oncogenic fusion events cannot be reliably detected from WES, so that RNA-Seq offers a better tool for gene fusion analysis.

Although this study used a 50 % tumor purity cutoff (as determined by a pathologist review of H&E sections adjacent to the tissue being sequenced), it is notable that many clinical specimens will fall below that threshold. Despite the 50 % cutoff, the estimate of tumor purity from WES and array data shows that 14 of the 46 tumor specimens may be falling below this threshold (Additional file 1: Table S11), highlighting known challenges in making tumor purity estimates. Importantly, analysis of WES and RNA-Seq identified actionable somatic alterations in samples containing as little as 25 % tumor cells (e.g. P0001, P0040) based on post-NGS purity estimation. As the price of sequencing continues to decline, it will be feasible to achieve higher sequencing depth in WES assays and resolve lower-purity tumors.

While our study demonstrates a clear benefit of WES and RNA-Seq over common targeted sequencing panels, the cost of WES and RNA-Seq remains an issue given their substantially higher price and the fact that today there is not a clear reimbursement mechanism for generating such data. Further, the data analyses and interpretations for the combined WES and RNA-Seq data take significantly more time to complete than data generated from targeted panel sequencing. Sample availability and quality also pose a barrier to performing genome-wide profiling. In addition, sequencing of gene panels, given they cover a small fraction of DNA compared to WES, is often performed at significantly higher sequencing depth, which allows for both increased sensitivity and specificity, given the heterogeneous mix of cells that comprise most tumor samples. However, the extent to which targeting of sub-clonal alterations can achieve clinical benefit is still under investigation.

Given the advantages and disadvantages to comprehensive sequencing, one could envision a staggered approach in which samples first undergo targeted sequencing and then progress to a deeper characterization if actionable alterations are not identified. Out of all DNA assays employed in our study, targeted panel sequencing had the highest data generation success rate (98/99 samples, 99.0 %; all samples were attempted regardless of available DNA mass), required the least input DNA (usually 30 ng, but lower input was accommodated), provided the fastest turnaround time, and produced the highest sequencing depth (mean 2587X), allowing detection of variants with allelic fractions as low as 5 % based on cell line dilution experiments (data not shown). Thus, a clinical pipeline should begin with a targeted panel (either a pan-cancer mutation hotspot panel as we employed, or a cancer-specific panel selected based on the patient’s diagnosis). A progression of increasingly comprehensive targeted panels is also possible. If no actionable alterations are identified, data generated from initial targeted panel sequencing may be informative to selecting parameters of follow-up assays, e.g. selecting the WES depth based on an initial tumor purity estimate from the panel (which would be based on non-actionable somatic variants). In our study, sufficient DNA mass was available for 41 out of 45 patients (91.1 %) to carry out WES. Although WES was successful for generating usable data for all 41 patients, eight of 41 patients (19.5 %) required multiple attempts, sometimes needing re-extractions of additional DNA from the tumor specimen, leading to delays (Additional file 2: Supplementary Results). Commercial kits for low-input, poor-DNA-quality WES library preparation are becoming increasingly available to ameliorate these issues and it is reasonable that more patients would be amenable to WES in the clinic in the future. Lastly, our concordance analysis of CNA from WES versus arrays data processed using the same CNA algorithm [61] (Additional file 2: Supplementary Results) highlights that the variability in CNA findings between platforms requires additional work to address. Thus, balancing the costs and benefits for different personalized genomics strategies is a rapidly evolving process.

Although cancer panel or exome sequencing based genetic testing is being broadly implemented, only a small portion of patients with actionable alterations followed the treatment recommendations through off-label use of drugs or enrollment into genotype-matched trials [11, 12, 14, 62]. In our study, according to the follow-up patient survey, only four out of the ten responders stated genomic findings altered treatment. The majority (21/31) of the patients we contacted did not even respond to the survey request. Previous studies described several major challenges in linking genomic findings to genotype-matched treatment [12, 14, 62], and these obstacles are present in our study as well. First, most of the patients were referred to Mount Sinai hospitals, and some of them did not return after genomic testing. Second, many patients in our study had gone through several lines of treatment at the time of genomic testing, and they were unlikely to be eligible for trials due to health deterioration and poor performance status. Third, genotype-matched trials may not be available, particularly for less common tumor types or less commonly mutated genes. Finally, long turnaround time of comprehensive genomic profiling in our study poses a significant barrier, similar to what has been reported by the Peds-MiOncoSeq consortium [14]. While many of the challenges are inherent to the overall design and the observational nature of current genomic-based PCT, we are taking measures for improvement such as reducing turnaround time in order to better realize the potential of genomics-driven individualized cancer treatment.

## Conclusions

We have developed and applied in a clinical setting an integrative genomic approach to facilitate PCT. Genomic profiling was performed to identify somatic mutations, copy number alterations, gene fusions, gene expression alterations, and germline variants to guide individualized cancer treatment. We demonstrate that the integrated genomic approach utilized in our study identified more cancer-relevant somatic mutations and more actionable alterations than several commercially available targeted cancer panels, and enabled us to elucidate the underlying cancer genetic drivers at the pathway level. Actionable alterations were found in 91 % of 46 cases, and the findings altered the course of treatment in four cases. More cancer-relevant somatic mutations and more actionable molecular alterations from multi-platform, genome level profiling are not surprising and provide a compelling argument that a comprehensive, integrative genomic approach significantly enhanced genomics-based PCT strategies.

## Additional files

Additional file 1: Supplementary Tables: Tables S1-S11. See Additional file 2 for detailed table legends. (XLSX 144 kb)

Additional file 2: Supplementary Material: Supplementary Results, Supplementary Methods, Supplementary Table legends. (DOCX 67 kb)

Additional file 3: Figure S1. A decision tree approach to predict drug response based on genetic alterations. (PPTX 96 kb)

Additional file 4: Figure S2. Detailed workflow of an integrative genomic approach in personalized cancer therapy. (PPTX 303 kb)

Additional file 5: Example PDF document of genomic findings returned to patients and their treating physicians in this study. Each section of this document was taken from an actual returned document, but to protect patient privacy, different sections were taken from different patient documents and information identifying the patient and specimens was removed. (PDF 367 kb)

Additional file 6: Figure S3A and B. Comparison of somatic CNA segment properties (number and length) between joint segmentation calls by saasCNV (https://zhangz05.u.hpc.mssm.edu/saasCNV) from WES data versus array data. Only samples where both assays were run on same DNA extraction are shown. All segments are shown, including those classified as “normal” (no CNA) and “undecided” (unclear CNA change). Patient P0040 is shown twice, once for each of the two tumors assayed, though in both cases the same normal control data is used. “FFPE” and “frozen” in all plots refers to tissue source of tumor DNA. **a** Correlation of total segment number per tumor between WES and array assays. **b** Violin plot comparing the distribution of segment lengths between WES and array assays for each tumor. Boxplot within each violin plot shows median, 25th, and 75th quantiles. (ZIP 229 kb)

Additional file 7: Figure S4. Correlation of somatic CNA fold-change (median “log2ratio” segment statistic from saasCNV of tumor with respect to normal) between WES and array data. The genome was split into non-overlapping partitions such that each partition begins and ends on a CNA segment break from either assay, but no CNA segment breaks occur inside any partition (“partition” feature of “bedops” software tool v2.4.14, https://github.com/bedops/bedops). Thus, each partition overlaps exactly one segment from WES data and exactly one segment from array data. Each point is a partition, with plotted values taken from the pair of segments that overlap it. Lower right corner shows the weighed (by partition length) Pearson correlation between WES- and array-derived log2ratio values for partitions, computed using the “corr” function from R (v3.2.1) package “boot” (v1.3-17). “FFPE” and “frozen” in all plots refers to tissue source of tumor DNA. (PPTX 1114 kb)

Additional file 8: Figure S5. Correlation of somatic CNA heterozygosity change (median “log2mBAF” segment statistic from saasCNV of tumor with respect to normal) between WES and array data. The same partitions are used as in Additional file 7: Figure S4, and weighed correlation in the lower right corner is also computed in the same way. (PPTX 981 kb)

Additional file 9: Figure S6. Comparison of weighed correlations of log2ratio (**a**, data from Additional file 7: Figure S4) and log2mBAF (**b**, data from Additional file 8: Figure S5) for assays on FFPE- versus frozen-derived tumor DNA material. *D* statistic and p-value from 2-sided KS test are shown for FFPE- versus frozen-derived correlation distributions (see "Methods" for details on KS test). (PPTX 76 kb)

Additional file 10: Figure S7. Distribution of somatic mutation allelic fractions in patient P0011, by mutation type. (PPTX 191 kb)

Additional file 11: Figure S8. A scatter plot of CCND1 gene expression versus log2 copy number ratio (tumor/normal). Each dot represents a patient tumor sample. Tumor types are color-coded. The two breast cancer patients where we reported CCND1 amplification are P0002 and P0040. (PPTX 112 kb)

Additional file 12: Figure S9A and B. CLPB-NADSYN1 gene fusion in patient P0002. **a** Long-range PCR confirms CLPB-NADSYN1 gene fusion. **b** Genomic breakpoint of CLPB-NADSYN1 gene fusion. (ZIP 150 kb)

Additional file 13: A gzipped tarball (.tgz file) of VCF files containing somatic mutations (i.e. present exclusively in tumor) for the 25 patients (one VCF file per patient) on whom paired normal/tumor WES was carried out and whose consents permitted public release of the full variant call data. For patients where multiple assays were carried out (WGS, WES, targeted panel, PacBio validation for troubleshooting), the VCF file contains the final variant call set where any discordance amongst the assays was resolved. Only mutations altering amino acid sequence (missense, nonsense, canonical splice site, indel) in a canonical isoform of a gene are given. Mutations are included that are not explicitly reported in returned findings due to lack of known relevance to cancer. Mutations rejected during our manual review protocol are not included. Thus, VCFs may contain mutations that were not manually reviewed. Three patients (P0009, P0025, and P0040) have two tumor specimens available, therefore those VCFs are multi-sample and report which mutations are recurrent among tumors and which are not; the rest are single-sample. (ZIP 37 kb)

Additional file 14: Figure S10. Sanger sequencing validation results of novel somatic EGFR mutation p.D587H (chr7:55233009G>C) in patient P0015. Sanger sequencing was carried out on normal and tumor DNA from this patient using forward and reverse primers (Beckman Coulter Genomics, Danvers, Massachusetts). Traces shown are as displayed by 4Peaks visualization software for Mac OS X (http://nucleobytes.com/4peaks). Two replicate sequencing reactions were carried out for each primer and sample combination, yielding the same result (second replicate not shown).  Base numbering shown is relative to priming site. The allelic fraction of p.D587H in tumor was 19.4 % (387/1998 reads have variant) in targeted panel sequencing, explaining the relatively small size of the Sanger peak for the alternate allele. (PPTX 240 kb)

## Abbreviations

CHPv2: Ion AmpliSeq Cancer Hotspot Panel v2
CNA: copy number alteration
EMR: electronic medical record
FFPE: formalin-fixed, paraffin-embedded
gDNA: genomic DNA
IHC: immunohistochemical or immunohistochemistry
MTC: medullary thyroid carcinoma
NGS: next-generation sequencing
OCP: Oncomine Cancer Panel
SNP: single-nucleotide polymorphism
SNV: single-nucleotide variant
WES: whole exome sequencing
WGS: whole genome sequencing
