# Supplementary material for: Development and clinical application of an integrative genomic approach to personalized cancer therapy
Source: Genome Med. 2016 Jun 1;8:62. doi: 10.1186/s13073-016-0313-0 (PMC4888213; doi:10.1186/s13073-016-0313-0)
Supplement: Supplementary file 2 — Supplementary Material: Supplementary Results, Supplementary Methods, Supplementary Table legends. (DOCX 67 kb) [file 13073_2016_313_MOESM2_ESM.docx]

**Supplementary Materials**

**Development and clinical application of an integrative genomic approach to personalized cancer therapy**

Andrew V. Uzilov^1^*, Wei Ding^1^*, Marc Y. Fink^1,2^, Yevgeniy Antipin^1^, Andrew S. Brohl^1,3^, Claire Davis^1^, Chun Yee Lau^1^, Chetanya Pandya^1^, Hardik Shah^1^, Yumi Kasai^1^, James Powell^1^, Mark Micchelli^1^, Rafael Castellanos^1^, Zhongyang Zhang^1^, Michael Linderman^1^, Yayoi Kinoshita^4^, Micol Zweig^1^, Katie Raustad^1^, Kakit Cheung^1^, Diane Castillo^1^, Melissa Wooten^1^, Imane Bourzgui^1^, Leah C. Newman^1^, Gintaras Deikus^1^, Bino Mathew^1^, Jun Zhu^1^, Benjamin S. Glicksberg^1^, Aye S. Moe^1^, Jun Liao^1^, Lisa Edelmann^1^, Joel T. Dudley^1^, Robert G. Maki^5^, Andrew Kasarskis^1^, Randall F. Holcombe^5^, Milind Mahajan^1^, Ke Hao^1^, Boris Reva^1^, Janina Longtine^4^, Daniela Starcevic^1^, Robert Sebra^1^, Michael J. Donovan^4^, Shuyu Li^1^, Eric E. Schadt^1#^, Rong Chen^1#^

^1^Department of Genetics and Genomic Sciences, Icahn Institute for Genomics and Multiscale Biology, Icahn School of Medicine at Mount Sinai, New York, NY 10029, USA.

^2^Department of Biomedical Sciences, Long Island University Post, Brookville, NY 11548, USA.

^3^Sarcoma Department, Moffitt Cancer Center, Tampa, FL 33612

^4^Department of Pathology, Icahn School of Medicine at Mount Sinai, New York, NY 10029, USA.

^5^Division of Hematology and Medical Oncology, Tisch Cancer Institute, Icahn School of Medicine at Mount Sinai, New York, NY 10029, USA.

*Contributed equally

# Corresponding authors: [rong.chen@mssm.edu](mailto:rong.chen@mssm.edu), [eric.schadt@mssm.edu](mailto:eric.schadt@mssm.edu).

TABLE OF CONTENTS

Supplementary Results 3

Sample availability, genomic data generation, primary genomic analysis and quality control (QC) 3

Concordance of somatic mutations identified by WES versus targeted panel sequencing 9

Concordance of CNA identified by WES versus SNP microarrays 10

Mutation spectrum in patient P0011 11

CLPB-NADSYN1 gene fusion in patient P0002 13

Supplementary Methods 14

Whole genome and whole exome sequencing 14

Targeted panel sequencing (Ion AmpliSeq Cancer Hotspot Panel v2, Life Technologies) 15

RNA-Seq 17

Genotyping using Illumina bead arrays 18

PCR primer design, amplification, and SMRT sequencing of targeted amplicons for variant validation on PacBio RSII 19

Identification of genomic alterations 21

Tumor purity estimation 25

Functional validation of EGFR p.D587H mutation 26

Supplementary Table Legends 28

References 31

# Supplementary Results

## Sample availability, genomic data generation, primary genomic analysis and quality control (QC)

We were able to obtain: paired normal/tumor DNA for 45 patients (for 44, DNA was extracted at Mount Sinai; for patient P0038, DNA was extracted externally), tumor-only DNA for 1 patient (DNA extracted at Mount Sinai), and raw data (FASTQ-format files) from normal/tumor WES done at an external laboratory for 1 patient (but no specimen was obtained). For the tumor-only patient, DNA quantity permitted attempting only the targeted panel, but no libraries were successfully generated after repeated attempts due to DNA quality issues; notably, this is the only instance where targeted panel sequencing failed in our study. For the 45 patients with normal/tumor DNA, raw sequence data generation on at least one NGS assay at Mount Sinai was successful for both normal and tumor. Therefore we successfully generated genomics data for 45/46 (97.8%) of patients with any DNA specimen, and after adding the sole patient with external data, we were able to analyze NGS data from 46 patients total (given in Table 1 and Additional file 1: Table S1).

Of the 45 patients with normal/tumor DNA obtained at Mount Sinai, tumor sources were: 24 patients from FFPE specimens, 17 from frozen tissue specimens, 1 from fine needle aspirate specimen, 2 from multiple types of sources, and 1 from unknown source (DNA extracted externally). We were also able to extract RNA from tumor and matched adjacent normal for 8 patients and from tumor only for 9 additional patients; RNA sources were either frozen tissue or tissue preserved in RNAlater stabilization reagent (Life Technologies). For 1 additional patient, raw data (FASTQ) from normal/tumor WES and tumor-only RNA-Seq was provided from an external laboratory, but no specimen was obtained. All specimens and attempted assays, and whether or not they succeeded, are given in Additional file 1: Table S2.

For the 46 patients with NGS data from DNA (45 having normal/tumor pairs where DNA was obtained and NGS data was generated at Mount Sinai, plus 1 with external data), data by type is as follows: 3 patients (P0001, P0002, P0004) had data from WGS, 42 from WES, 42 from targeted panel sequencing, 31 from array. Each WES, WGS, array, and all except one (patient P0005) targeted panel dataset contained paired normal and tumor. For each patient who had WGS or array, WES was also done. Every patient for whom WES was done also had targeted panel sequencing except for 3 patients (P0001, P0002, P0004) early in the study before targeted panel sequencing became part of the study workflow. 5 patients had more than one tumor sequenced on targeted panel, of whom 4 (P0005, P0009, P0025, P0040) had WES for the same specimens (i.e. WES was done on multiple tumor samples, same DNA as used for targeted panel). Out of the 45 patients with normal and tumor DNA at Mount Sinai, neither WES nor array were attempted on 4 (8.9%; patients: P0015, P0016, P0036, P0038) and array was not attempted (but WES done) on an additional 3 (6.7%; patients: P0032, P0039, P0044) due to insufficient DNA quantity from available tumor tissue; however, targeted panel sequencing was successful on all of those. An additional 7 (15.6%) patients (P0001, P0002, P0003, P0004, P0026, P0012, P0017) early in the study did not have array data because this was not yet part of the study workflow.

Whenever enough DNA was available (41/45 patients, 91.1%), WES was attempted and eventually successful (i.e. passing all QC) in all cases, yielding median de-duplicated, on-target coverage (sequencing depth) of 66.1X for normal and 112.0X for tumor. All assay details and QC statistics for WES are given in Additional file 1: Table S6. The primary cause of sample-to-sample variance in depth appears to be the percentage of paired-end reads that are flagged by Picard as potential PCR duplicates and are discarded by the GATK pipeline prior to variant calling, which decreases depth. Duplication was worse (KS 2-sided test, D=0.48, p=0.00917) for FFPE-derived tumor DNA (N=21, median 46.2% duplicate reads) than for frozen-derived (N=22, median 28.6% duplicate reads) when comparing only samples done on the most commonly used enrichment protocol (Nimblegen). In a more strict comparison, we can subset this data to only samples sequenced using the 2:1 tumor/normal multiplex protocol, in order to adjust for variance in duplication due to depth (because duplication increases with depth); however, we still find that duplication is worse for FFPE-derived DNA (KS 2-sided test, D=0.63, p=0.00506; FFPE N=15, median 39.1%; frozen N=12, median 25.0%). There is no difference in duplication percentage between the two different WGS library preparation kits used prior to WES enrichment (KS 2-sided test on NEBNext versus KapaHyper WGS kits: D=0.27, p=0.8797, N=15 versus N=6 resp., median 48.7% versus 41.5% resp. when comparing FFPE-derived tumor DNA; D=0.20, p=0.959, N=13 versus N=9 resp., median 29.8% versus 25.3% resp. when comparing frozen-derived DNA). This indicates that although WES from FFPE-derived DNA is feasible, it is highly preferable to use frozen-derived DNA for less duplication and higher depth, which leads to higher sensitivity at the same cost per sample.

To achieve our 100% success rate on WES, multiple tries were required in some cases (Additional file 1: Table S2 and S6). For 5/41 (12.2%) patients (P0028, P0029, P0030, P0043, P0046), libraries were not successfully prepped on the initial attempt, requiring one or more re-tries to pass pre-sequencing library QC. For 3/41 (7.3%) additional patients (P0020, P0031, P0037), prepped libraries were re-sequenced on additional runs to yield more sequencing depth, as original depth was deemed insufficient.

Although raw array data was generated from paired normal/tumor DNA for 31 patients, data was not usable for 5 (16.1%) patients (P0029, P0030, P0031, P0042, P0043) even after a re-run of the assay due to low call rate in either normal or tumor or both, suggesting a sample quality issue. Therefore, arrays had the highest failure rate of all assays. Array data for two patients (P0011 and P0021) was excluded from analysis for confounding biological reasons explained later in this section. One patient (P0009) with two tumors yielded usable array data for one tumor but not the other. Thus, in this paper, we analyze array datasets from 24 patients, of whom a single patient (P0040) has array and WES data on two tumors, although the array data for that individual was retroactively produced after genomics findings were already returned.

For three patients (P0001, P0003, and P0005), bioinformatics analysis revealed that some of the specimens had very little tumor content; comparative analysis of all specimen data from both WES and targeted panel sequencing was essential to troubleshooting the situations in P0003 and P0005. For patient P0003, targeted panel sequencing on both tumor specimens revealed that the EGFR exon 19 deletion mutation known from clinical testing had an allelic fraction of 25.9% in the FNA-derived specimen (Additional file 1: Table S2) and 5.3% in the frozen specimen, suggesting the latter specimen had substantially lower tumor purity; however, the latter specimen was the one selected for WES based on initial pathological assessment. Consistent with this, no plausible somatic mutations were called from WES sequencing, even after re-sequencing to increase depth. However, evidence for all 3 driver somatic mutations identified from targeted panel sequencing (EGFR exon 19 del, SMAD4 p.S144*, and TP53 p.R124fs) was observed in both WES and RNA-Seq data for the frozen specimen after retrospective manual review, albeit at an allelic fraction too low to have yielded *de novo* variant calls. For patient P0005, WES was carried out on DNA from both the primary tumor (colon specimen, Additional file 1: Table S2) and a metastatic tumor (liver specimen). Although somatic variant calls were in concordance between the two specimens, allelic fraction analysis showed that the tumor DNA fraction of the metastatic specimen was likely <10% based on the upper bound of the allelic fraction distribution. Targeted panel sequencing, as well as subsequent validation of somatic variant calls on targeted custom amplicon sequencing on an independent platform (PacBio RSII), both confirmed the low allelic fraction distribution of the metastatic specimen.

Lastly, 4 patients (P0010, P0011, P0021, P0041) for whom assays were technically successful nevertheless required special handling and troubleshooting due to confounding results that have a biological explanation. The first patient (P0010) had somatic mutation p.K462fs in the gene RB1 on the targeted panel assay, but not the WES assay; notably, two different DNA extractions (i.e. spatially distinct regions of the tumor) were used for the two assays. We confirmed by targeted custom amplicon sequencing on an independent platform (PacBio RSII), as well as re-sequencing of both extractions on the targeted panel, that the two extractions were indeed genetically different for the RB1 mutation, but the same for the RET p.C634R germline driver variant (preserved in tumor). Moreover, PacBio targeted amplicon sequencing showed that 6 other somatic mutations discovered on WES were identical between the RB1-containing and RB1-absent extractions. This indicates that the tumor was heterogeneous for the RB1 mutation and that sample mix-up is not the explanation. The second patient (P0011) had a bone marrow donation from a family member and thus the blood DNA control contained an uneven mixture of two genomes (excess heterozygosity) that confounded analysis. For this reason, no CNA analysis was done, though somatic variant findings were consistent with previously done clinical NGS testing (FoundationOne). The third patient (P0021) yielded array data where variants matched those from WES data, but had lack of CNA. In this case, the DNA extraction sequenced on WES was exhausted and the array experiment had to be done on a different extraction, possibly indicating that the specimen was heterogeneous and thus the second extraction contained little or no tumor DNA. For the last patient (P0041), adjacent normal tissue was used for WES and targeted panel assays because a blood draw was unavailable at the time. However, sequencing reads supporting the RET p.M918T driver mutation were found in the normal, albeit at a lower allelic fraction than tumor (68.4% versus 5.7% on targeted panel, 72% versus 6% on WES); the adjacent normal RNA-Seq data also showed evidence of this mutation. Blood-derived DNA was later obtained and sequenced on the targeted panel, showing no evidence of germline RET p.M918T. This indicates that the adjacent normal was contaminated with tumor cells, possibly due to imperfect resection. As a result, genuine somatic mutations were filtered by the MuTect tool because they contained evidence in the normal control, albeit at lower level. This motivates the use of blood DNA as normal control and strict workflows that avoid cross-sample contamination.

Targeted panel sequencing succeeded on 98/99 (99.0%) of DNA specimens from 42 patients, yielding the following mean QC values: 2,578X sequencing depth, 96.8% uniformity, and 96.7% on-target reads (Additional file 1: Table S7). A mean of 15.78 variant calls per specimen was obtained, which are primarily common germline polymorphisms. We failed to generate libraries for only one specimen due to poor specimen quality.

We successfully obtained tumor RNA-Seq data from 18 patients (RNA for 17 of which was extracted and sequenced at Mount Sinai; 1 received as FASTQs from external provider), of whom 8 also had RNA-Seq on adjacent normal tissue specimen usable as a control. The specimens, RNA Integrity Number (RIN), sequencing protocols used, and read counts are summarized in Additional file 1: Table S8. For the 28 Sinai-sequenced specimens, we yielded a mean of 67.9 million clusters (paired-end reads) and median 51.9 million clusters per specimen; the difference between mean and median is due to the fact that some samples were sequenced to a higher depth early in the study to generate technical replicates for accuracy assessment, and for final analysis, replicate data was pooled. Notably, because P0005 (metastasis only) and P0003 tumors contained very little tumor cells based on whole-exome analysis, their RNA-Seq data was not useful for analysis; manual review in IGV for evidence of tumor expression of somatic mutations identified from WES was consistent with the hypothesis that RNA-Seq data also contains very little tumor RNA, as the allelic fractions of expressed mutations were low and at a level consistent with the tumor purity estimate from WES.

We applied our fusion detection pipeline to RNA-Seq data from 16 tumor and 8 adjacent normal specimens from 14 patients (excluding P0001, P0003, and P0005 due to low tumor purity issues identified from WES, as described above, and P0033 because no RNA specimen was available to validate at Mount Sinai). At least one fusion prediction called by two or more algorithms was identified in 12 tumors (mean 3.92, median 3, range 1-15, standard deviation 3.64 per tumor). For 5 of these tumors where an adjacent normal was available, we identified mean 2.6 (median 2, range 1-5) somatic fusions per tumor.

## Concordance of somatic mutations identified by WES versus targeted panel sequencing

We examined concordance between somatic variant calls from WES versus from targeted panel for 33 patients where normal/tumor data was successfully generated on both assays and both assays were run on the same tumor DNA extraction (to remove confounding effects from genetic intra-tumor heterogeneity). This analysis was done only on variants in the 22,027 nt of the genome that are covered by the targeted panel design. Only non-synonymous and splice-site-altering somatic SNVs and indels passing the population frequency filter were considered. We found that for 20 patients, variant calls were identical between the two assays (mean 1.8 SNVs and 0.24 indels per patient), and for 8 patients no variant calls were made in the targeted regions; thus, 28/33 (84.8%) patients had data in exact concordance between the two assays. For the 5 patients where calls were discordant (Additional file 1: Table S9, mean 1.4 discordant calls per patient), it was always due to the targeted panel pipeline calling variants not called by the WES pipeline. Upon manual review, we observed low-level evidence (allelic fraction <= 0.06) for the discordant calls in WES read alignments for 3 patients (P0011, P0024, P0043), although the corresponding allelic fraction in targeted panel data was always higher, indicating that the mutation-containing locus was either under-sampled in WES or over-sampled in targeted panel sequencing. For P0011, all 3 discordant mutations were called in a clinical assay (FoundationOne) carried out prior to patient enrollment, thus they are false negatives on the WES assay. For another patient (P0005), the allelic fraction was very low (~0.04) in both assays due to low tumor purity, thus although WES read alignments had some data supporting the variant, the call was not made in that pipeline; additionally, because the discordant variant is complex, it would not have been callable using the somatic variant callers employed. For the last patient (P0012), the discordant call was not made in the WES pipeline due to overly strict quality filtering by the MuTect tool, which removed the call due to low-level evidence of variant present in the normal sample. The discordant patient set has 3 tumor specimens from FFPE and 2 from frozen tissue, thus there does not appear to be enrichment in discordance in FFPE-derived DNA over frozen. As targeted panel depth is substantially higher than WES depth (compare Additional file 1: Table S6 and Table S7), it is likely that the discordance is driven by under-sampling of the mutated allele in WES, suggesting that the targeted panel is more sensitive than WES in the targeted regions.

Assessment of concordance between WES and external panels that were performed on a small subset of patients (3 panels on 2 patients) demonstrated that WES found all mutations reported in the external panels.

### Concordance of CNA identified by WES versus SNP microarrays

We examined concordance between copy-number alterations determined by the same saasCNV algorithm using data from WES versus from arrays. This was done for 22 tumors (from 21 patients, because patient P0040 had two tumors with different purities interrogated) where normal/tumor data was successfully generated on both assays and both assays were run on the same tumor DNA extraction (to remove confounding effects from genetic intra-tumor heterogeneity, just like in the targeted panel versus WES concordance analysis). We examined all segments (regardless of CNA classification) identified by the saasCNV joint segmentation algorithm, which takes as input both log2ratio and log2mBAF values from one of the assays. As the arrays are designed to interrogate non-exonic regions and thus yield data containing more heterozygous SNVs than WES for the saasCNV algorithm, it is expected that the arrays are providing better resolution of segment boundaries. We observe that the number of segments per tumor tends to be greater for array-based data (Additional file 6: Fig. S3A; median 1.7X more segments than the corresponding WES data) for both FFPE-derived and frozen-derived DNA, and the variability in segment lengths tends to be higher in WES data (Additional file 7: Fig. S3B). The total fraction of the genome covered by segments was comparable between the two assays (median 7.54 Mb difference in size of genome covered). Then, for each normal/tumor pair, we partitioned the genome into non-overlapping regions (as described in Fig. S4 caption) and compared log2ratio and log2mBAF (Additional file 8: Fig. S4 and Additional file 9: Fig. S5) for the two overlapping sub-segments in each partition. We find that the correlation in log2ratio between the two assays tends to be weaker for FFPE-derived samples than for frozen-derived, but the correlation between log2mBAF is equivalent and high (Additional file 10: Fig. S6). This suggests that our power to identify loss of heterozygosity regions is equivalent between WES and array assays regardless of tissue preservation method, but copy-number change estimates are less reliable for FFPE-derived DNA.

### Mutation spectrum in patient P0011

We noticed that one patient (P0011) had an unusually high number and frequency of somatic mutations (11 indels and 810 SNVs that are non-synonymous or alter splice site in any isoform, i.e. 24.8 mutations per megabase) compared to other patients in our cohort (Fig. 2) and to published somatic mutation frequency statistics from whole-exome studies [1, 2]. A surprisingly high fraction (634/810, or 78.3%) of SNVs are C>T transitions (see Methods for mutation nomenclature). When examining the bases immediately upstream and downstream of the mutated base, we noticed that base C in C>T mutations tended to be flanked by pyrimidines, with TpCpC>TpTpC and CpCpC>CpTpC mutations being the most common (19.9% and 11.5% respectively, Additional file 1: Table S10).

Our mutation frequencies are most consistent with Signatures 7, 11, and 19 from [1], which have a prevalence of 5.0%, 0.6%, and 0.2% respectively in cancer samples examined in that work. As reviewed in [3], Signature 7 is believed to be caused by pyrimidine and dipyrimidine lesions from UV radiation repaired by transcription-coupled DNA repair, and therefore should include higher frequencies of dinucleotide pyrimidine mutations and a transcriptional strand bias. We do not observe transcriptional strand bias (data not shown), but we do notice that 70 SNVs (8.6%) from our MuTect variant-calling pipeline are immediately adjacent and can instead be interpreted as 35 dinucleotide mutations, of which 29 (82.9%) are CpC>TpT substitutions, lending some support for Signature 7. Signature 11 is believed to be caused by O^6^-methyl-guanine lesions, which can be induced by temozolomide (which we do not see in the patient’s treatment history); Signature 19 does not have a mechanistic explanation. It is therefore not clear which of these signatures is the primary mutagenic driver here, and we cannot rule out the presence of multiple confounding mutagenic processes.

We observe that most mutations fall in the 0.10-0.15 allelic fraction range (Additional file 11: Fig. S7; median 0.13, max 0.34), suggesting that the tumor DNA content of our specimen may be <= 34%. These frequencies are also consistent with the possibility that the majority of observed mutations (assuming they are heterozygous mutations on normal diploid DNA) were present in a single cell that underwent clonal expansion during tumorigenesis. Thus, we propose that a single massive mutagenic event occurred at a single time point early in the tumor’s history, as opposed to the model of progressive accumulation of sub-clonal mutations.

The clinical history of this patient is remarkable in that over 50 squamous cell carcinoma (SCC) skin tumors have been removed by Mohs procedures in the 3 years since diagnosis, of which we sequenced only one. Moreover, 8 years prior to the SCC diagnosis, this patient was diagnosed with acute myeloid leukemia (AML) for which he received an allogeneic bone marrow transplant from an HLA-matched donor. No AML relapse is known to have occurred, but severe graft-versus-host disease (GVHD) developed for which many drug treatments were attempted. The most recent treatment prior to development of symptoms that were diagnosed as SCC was alemtuzumab. Cytomegalovirus (CMV) re-activation is a complication of alemtuzumab therapy in GVHD treatment [4], and increasing evidence suggests that CMV may be oncogenic in other cancers, primarily gliomas [5], though it is not obvious whether this is related to our observed somatic mutation frequencies. Thus, while somatic mutation frequencies identified from WES show a striking, unusual pattern, it is not clear whether they are informative to treatment given the patient’s clinical history.

### CLPB-NADSYN1 gene fusion in patient P0002

Our RNA seq fusion analysis pipeline predicted an out-of-frame, CLPB-NADSYN1 fusion in a frozen tumor sample from patient P0002, with the 5’ part of CLPB mRNA fused to the 3’ part of NADSYN1 mRNA. WGS was also performed on the same tumor sample. Analysis of the WGS data supported RNA seq based fusion discovery and suggested fusion occurs between intron 12 of CLPB and intron 5 of NADSYN1. We further validated this fusion event by long range PCR. Fusion mRNA derived PCR product is only detected in the tumor sample, but not in the blood or adjacent normal controls (Additional file 13: Fig. S9A). Mapping of the breakpoint confirmed the fusion takes place in the intronic regions of the 2 fusion partners (Additional file 14: Fig. S9B). This out-of-frame fusion causes frame shift in the coding region of NADSYN1, and is expected to generate a pool of peptides as neo-antigens on tumor cell surface. Based on these results, it is suggested patient P0002 could benefit from immunotherapy, as recent progress in developing cancer immunotherapy has identified increased neo-antigen load as a potential biomarker for immunotherapy response [6].

# Supplementary Methods

### Whole genome and whole exome sequencing

If the gDNA was isolated from FFPE material, 0.5 μg of the starting genomic FFPE DNA was repaired using the PreCR Repair Mix (New England Biolabs, Ipswich, MA) following the manufacturer’s instructions. Success of the repair was determined by an increase in size as evaluated by using the 2100 Bioanalyzer (Agilent, Santa Clara, CA). The entire repaired material was then used for the construction of the gDNA library. If the gDNA was isolated from frozen tissue and deemed intact as assessed by the BioAnalyzer results, the repair step was skipped and went directly to shearing. In some cases, when a library could not prepared from non-FFPE gDNA, we found that use of the PreCR Repair Mix allowed for a library to be made. Initial shearing of 0.5–1 µg genomic DNA to a mean of 200-300 bp fragments was performed using the Covaris E210 focused acoustic energy system (Covaris, Woburn, MA). Whole genome libraries were prepared using either the NEBNext DNA Library Prep kit or KAPA Hyper Prep kit (Kapa Biosystems, Wilmington, MA) according to the standard manufacturer's protocol (New England Biolabs, Ipswich, MA). Illumina compatible paired-end adapters were used and the adapter-ligated DNA fragments was amplified by ligation-mediated PCR (KAPA Biosystems, Wilmington, MA) using a reverse PCR primer containing a six nucleotide barcode that allowed for multiples samples to be pooled and sequenced in the same run. The library was enriched for exomic sequences with the SeqCap EZ Human Exome Library v3.0 capture system (Roche NimbleGen, Madison, WI). The libraries were then sequenced with a 100bp paired-end protocol on the Illumina HiSeq 2500 according to standard manufacturer's protocol (Illumina, San Diego, CA). Towards the end of the study, the Illumina compatible libraries were prepared using the SureSelect XT library preparation system and the whole exome regions were captured using the SureSelect Human All Exon V5 system following the standard manufacturer's protocol (Agilent, Santa Clara, CA). For the SureSelect based preparations, the adaptors and amplification primers that came with the SureSelect kits were used. Approximately 8-14 pM of the whole exome libraries were clustered and run on either the high output or rapid run HiSeq 2500 flow cell and sequenced for 100bp paired-end reads according to the standard manufacturer's protocol (Illumina, San Diego, CA). Usage of PreCR mix, library preparation protocol and number of sequencing reads generated for each sample are given in Additional file 1: Table S6.

### Targeted panel sequencing (Ion AmpliSeq Cancer Hotspot Panel v2, Life Technologies)

Tumor and normal DNA samples were received in the extracted genomic DNA (gDNA) format. Qubit spectrophotometry was then performed using 1 uL of each sample to quantify the concentration and mass and the Agilent Bioanalyzer DNA 12000 chip was utilized to assess the gDNA integrity in the 100 bp to 17,000 bp range prior to library preparation to eliminate any low quality input material.

The DNA samples that pass qualification proceeded into library construction using the manufacturer’s Ion Torrent (Thermo Fisher Scientific) AmpliSeq library preparation protocol for the Hotspot v2 protocol. Briefly, to create amplicons for library construction, 30 ng of input DNA in up to 12 uL was mixed with 4uL of 5X Ion AmpliSeq HiFi Mix, 4uL of 5X AmpliSeq pooled primers specific to the Hotspot v2 loci, and the remaining volume of Nuclease Free water to adjust the reaction volume to 20 uL. Once thoroughly mixed, the amplification was conducted at 99^o^C for 2 minutes followed by cycling 17 times for stand DNA (20 for FFPE samples) at 99^o^C for 15 seconds, then 60^o^C for 4 minutes, followed by holding at 10^o^C for up to 1 hour. After centrifuging, 2uL of FuPa digestion reagent was added and samples were placed in the thermal cycler and held at 50^o^C for 10 minutes, then 55^o^C for 10 minutes, 60^o^C for 20 minutes and finally at 10^o^C for up to 1 hour. While the FuPa partial digestion was taking place, barcodes were prepared using 2uL Ion P1 adapter, 2uL of the Ion Xpress barcodes, and 4uL of nuclease free water. After the completion of FuPa digestion, the samples were mixed with 4uL of switch solution, 2uL of barcode and 2uL of DNA ligase into the ligation reaction on the thermal cycler by holding at 20^o^C for 30 minutes, then 72^o^C at 10 minutes, and 10^o^C for up to 1 hour. Once the DNA ligase reaction was complete, each library was taken to room temperature and purified on Agencourt AMPure XP beads at a volume ratio of 1.5X. After sample elution from the magnetic beads, 50 uL of Platinum PCR SuperMix High Fidelity and 2uL of the Equalizer primer was added to equilibrate the concentrations of each library in the pooled mixture. These reagents were placed on the thermal cycler and held at 98^o^C for 2 minutes, then cycled 7 times at 98^o^C for 15 seconds, 64^o^C for 1 minute and after cycling the samples are held at 10^o^C for up to 1 hour.

Once the reaction was completed, samples were centrifuged and 10 uL of the Equalizer Capture solution was added to each sample and incubated at room temperature for 5 minutes. During the incubation, 3uL of Equalizer beads was added per sample into a clean strip tube alongside 6 uL of Equalizer Wash Buffer and then the beads were placed on the magnet to separate for 3 minutes and then the supernatant was discarded without disturbing the bead pellet. After the beads were removed, another 6uL of the Equalizer Wash Buffer was added and re-suspended and the 6uL of the washed Equalizer beads were added to the samples containing the capture reaction and incubated at room temperature for 5 minutes. After magnetic separation, the supernatant was removed without disturbing the pellet, and 150 uL of Equalizer Wash Buffer was added to each reaction and held for 1 minute and then washed for a second time and as much wash buffer as possible was removed. After drying, the samples were removed from the magnet and 100 uL of Equalizer Elution Buffer was added to each pellet and libraries were eluted by incubation in the thermal cycler at 32^o^C for 5 minutes and the libraries were collected off the magnetic bead station and the supernatant contains the equalized libraries, which equates to approximately 100 pM each. At this point, multiplexing was done as shown in Additional file 1: Table S7 for the various PGM chip types employed.

After libraries were completed and equalized, the Ion Chef was used to concentrate and load the selected PGM chip by first placing all consumables and cartridges onto the deck of the Chef per the Ion PGM Chef Kit User Guide. While allowing the reagents to reach room temperature for at least 40 minutes, the Torrent Chef and run conditions were configured for AmpliSeq Cancer Hotspot v2 using the 4475346_CHP2_hotspots_20120927 file for hotspot regions and the 4475346_CHP2_designed_20120806.bed file for targeted regions and the built-in PGM “somatic – low stringency” preset in TorrentSuite version 4.0. Once consumables have reached room temperature, the appropriate 3.14, 3.16, or 3.18 chips were placed onto each of the Chef’s centrifuge buckets and the PGM configuration was run by manufacturer’s suggested protocol until completed.

Chips are then loaded onto the PGM, the instrument was initialized and cleaned using manufacturer’s guidelines, prior to each run to achieve the best signal to noise. After initialization stabilizes, the chips were inserted into the PGM following chip calibration and initiation of sequencing once all criteria are met. After sequencing was completed, the variant calling was automatically completed using the aforementioned settings against the target files mentioned. Any custom analysis is detailed in the analysis section.

QC statistics, multiplexing details, variant caller version, and chip type are given in Additional file 1: Table S7.

### RNA-Seq

RNA integrity was checked by either the Agilent 2100 Bioanalyzer using the RNA 6000 Nano assay, RNA 600 Pico assay or with the Agilent 2200 TapeStation using the R6K ScreenTape (Agilent, CA, USA). When the total RNA samples had RIN value 7.0 or greater, and no evidence of degradation from review of peaks was seen for all samples belonging to a patient, the RNA underwent poly-A-selection. If at least one sample from a patient had a RIN value 7.0 or less, then all RNA samples from the same patient underwent ribosomal RNA (rRNA) depletion using the Ribo-Zero Gold rRNA Removal Kit according to the manufacturer’s instructions (Illumina, San Diego, CA). Before selection, the ERCC ExFold RNA Spike-In Mixes (Life Technologies, Grand Island, NY) were added to many of the RNA samples according to manufacturer’s protocol. This was used to assess the performance of the RNA library preparations and sequencing [7]. The RNA sequencing library was prepared with the standard TruSeq RNA Sample Prep Kit v2 protocol (Illumina, CA, USA). Briefly, the remaining RNA after poly-A-selection or rRNA depletion was fragmented. The cDNA was synthesized using random hexamers, end-repaired and ligated with appropriate adaptors for sequencing. The library then underwent size selection and purification using AMPure XP beads (Beckman Coulter, CA, USA). The appropriate Illumina recommended 6 bp barcode bases are introduced at one end of the adaptors during PCR amplification step. The size and concentration of the RNA-Seq libraries was measured by Bioanalyzer and Qubit fluorometry (Life Technologies, NY, USA) before loading onto the sequencer. The RNA libraries were sequenced on the Illumina HiSeq 2500 System with 100 nucleotide paired-end reads, according to the standard manufacturer's protocol (Illumina, CA, USA). RIN, ERCC spike-in mix added, library preparation type and reads generated for each sample are given in Additional file 1: Table S8.

### Genotyping using Illumina bead arrays

Genotyping on Illumina HumanOmniExpressExome v1.2 BeadChip arrays (8-sample format) on Infinium HD was carried out following Illumina recommend protocol. Briefly, around 750 ng each of DNA samples were denatured and neutralized to prepare them for amplification. The denatured DNA was isothermally amplified in an overnight incubation at 37^0^C. The whole-genome amplification uniformly increases the amount of DNA by several thousand-fold without introducing large amounts of amplification bias. The amplified product was enzymatically fragmented to 300-600 base pairs and purified by isopropanol precipitation. The precipitated DNA was resuspended in hybridization buffer providing ideal conditions for beadchip hybridization. Hybridization of sheared DNA to the bead chip was carried out in a capillary flow through chamber. Samples were pipetted into the BeadChip and incubated overnight at 48^0^C in the Illumina Hybridization Oven. The amplified and fragmented DNA samples anneal to locus-specific 50-mers during hybridization. One bead type corresponds to one locus. Unhybridized and non-specifically hybridized DNA was washed away using phosphate buffer, and the BeadChip was prepared for staining and extension. Single-base extension of the oligos hybridized on the BeadChip was carried out to incorporate a single base that denotes the SNP genotype using biotin-labeled dideoxy G and dideoxy C nucleotides, and dinitrophenyl (DNP) labeled dideoxy A and dideoxy T nucleotides. Green fluorescent Streptavidin and red fluorescent Anti-DNP Antibody were used to bind specifically to the labeled probes. These fluorescently labeled beads arrays were scanned using Illumina HiScan using laser to excite the fluorophore of the single-base extension product on the beads. The scanner records high-resolution images of the light emitted from the fluorophores. The Hiscan uses red and green lasers to measure each bead on the array. It generates fluorescence intensity data that is used to score genotype calls for each SNP. The scanned data were analyzed on Genome Analyzer software provided by Illumina, Inc.

### PCR primer design, amplification, and SMRT sequencing of targeted amplicons for variant validation on PacBio RSII

For each patient sample and desired genetic locus, individual primer pairs were designed for targeted sequencing purposes. Each primer pair generated amplicons that were 500-2000nt in length. Primers were designed using Primer3 software (Whitehead Institute for Biomedical Research, Cambridge, MA) and the NCBI Primer Blast website tool (NLM). Each PCR reaction had a total of 50uL volume and used 10ng of genomic DNA, 0.5uL of TaKaRa LA Taq (5 U/uL, TaKaRa-Clontech, Othu, Shiga, Japan), 5.0uL of 10X LA PCR Buffer (Mg2+ free), 5.0uL MgCl2, 8.0uL dNTP Mixture (TaKaRa), 10uL of 5M Betaine (Affymetrix, Santa Clara, CA) and 4.0uL of each primer mixture at 10pmol/uL. Thermocycler parameters varied per individual amplicon, but each used a touchdown PCR thermoprofile. The general program was as follows: primary denaturation at 94^o^C for 1 minute, followed by 30 cycles of 98^o^C for 10 seconds, 55^o^C (annealing gradient) for 45 seconds, and 68^o^C for 5 minutes; then a denaturation step of 30 cycles of 98^o^C for 10 seconds, 46^o^C for 45 seconds, and 68^o^C for 5 minutes (extension step); followed by 72^o^C for 10 seconds and a 4^o^C hold. Each PCR reaction was performed on an Eppendorf Mastercycler Pro v3.024. Purification on PCR reactions were done using AMPure PB beads (Pacific Biosciences, Menlo Park CA), and quantification was performed using the Qubit dsDNA HS Assay Kit (ThermoFisher, New York, New York) for concentration and the Agilent DNA 12000 Kit (Agilent Technologies, Danbury, CT) to measure amplicon size.

Equivalent molar quantities of PCR products based on concentration and DNA length were pooled and subjected to single-molecule real-time sequencing using the RSII instrument (Pacific Biosciences, Menlo Park, CA, USA).  Sequencing was performed according to the Pacific Biosciences protocol using the size range most appropriate for the pooled amplicons. In brief, the pooled PCR amplicons were purified using AMPure PB beads at 0.6X fold volume.  SMRTbell libraries were constructed using end-repair, ligation, and exonuclease purification strategies detailed in the Pacific Biosciences’ commercial Template Preparation and Binding Kit protocols. Then SMRTbell templates were bound to polymerase molecules for 4 hours at 25^o^C using 3nM of the amplicon SMRTbell library and 3X excess P4 or P6 DNA polymerase at a concentration of 9nM. The polymerase-template complexes were immobilized at 25-150 pM for 30 minutes on SMRTcells containing an array of zero-mode waveguides (ZMWs). 150,000 ZMWs were analyzed for sequencing to generate reads with an mean read length of ~25,000 bp using a 1x240-minute collection protocol. Circular consensus sequencing (CCS) was then employed to use multiple passes on each SMRTbell to generate CCS reads with higher accuracy for data analysis using the RS_ReadsofInsert.1 pipeline in SMRT Portal software (versions 2.1 through 2.3, as several upgrades were released overtime). Resulting reads in FASTQ format were aligned to the hg19 human reference genome using bwa-mem 0.7.5a-r405 and manually inspected in IGV to determine whether they supported the variant call that needed to be validated.

### Identification of genomic alterations

*Variant calling.*  For each patient individually, FASTQ files from all available WGS or WES runs were combined into a cohort and run through an in-house pipeline [8] to yield BAM and VCF files with germline and somatic variant calls (SNVs and small indels). Briefly, this in-house pipeline implements Genome Analysis Toolkit (GATK) [9] version 2.7 best practices for alignment, base quality recalibration, variant calling (using HaplotypeCaller), and variant quality score recalibration (VQSR) [10, 11]. The same bioinformatics protocol was applied to all DNA samples regardless of whether they were derived from frozen tissue, FFPE tissue, or blood. VQSR was set to 99.5% sensitivity. Read pairs whose 5’ coordinates were identical were marked (except for the best read pair) as duplicates by the Picard software (<http://broadinstitute.github.io/picard>) and are not used for variant calling, per the above best practices, in order to ensure that evidence for each variant is coming from distinct DNA molecules and thus avoiding over-counting possibly over-amplified or over-sampled DNA. Target regions were defined by taking the design file from the WES hybridization-capture kit manufacturer (see WES section in Supplementary Methods and Additional file 1: Table S6) and adding 100nt padding, then merging to remove overlap; variant calling, as well as depth and other QC statistics computation, was only done within these target regions. For somatic variant calling, MuTect [12] (version 1.1.6-10b1ba92, HC+PON mode with default settings, using COSMIC [13] version 65, dbSNP [14] version 137, and using variant calls from matched normal as the “panel of normals” setting) and Varscan2 [15] (version 2.3.5, with flags *--tumor-purity 0.5* and *--min-var-freq 0.07*) were used. A manually curated panel of pharmacogenomics variants was force-called in the normal sample using GATK’s UnifiedGenotyper to ensure discrimination between homozygous reference variants and variants that cannot be called.

All variant calls were annotated with SnpEff v3.4i [16] (using the Ensembl [17] version 74 / GRCh37 resource bundle) and loaded into a custom MySQL (Percona MySQL Server Community Edition 5.6.14-rel62.0.483.rhel6) database schema using in-house scripts, where they were filtered as follows. Only variants annotated as altering the amino acid sequence (missense, nonsense, affecting a canonical splice site, indel) were retained for interpretation; however, the full set of variants was used for routine post-sequencing QC described below. Variants were separated into 4 tiers according to the variant/gene annotation by SnpEff. Tier 1 genes are those that have previously been associated with the patient’s specific cancer type based on internal, manually-curated gene lists.  Tier 2 genes are pan-cancer genes [18, 19]. Tier 3 genes are all cancer-associated genes based on internal, manually-curated gene lists. Tier 4 genes are all genes that are not tier 1-3. Importantly, for producing the findings document, tier 4 is additional split into two tiers (tiers 4 and 5, as described in Methods section “Generation of summary documents”). Germline variant calls were those marked “PASS” by VQSR in the “FILTER” column and having a called, non-reference GT in the normal sample. Somatic calls whose population allele frequency in either ESP5400 (<http://evs.gs.washington.edu/EVS/>) [20] or 1000Genomes [21] exceeded 2% were discarded on the presumption that they are any combination of: contamination, a variant present but missed in normal sample, a low-level artifact, could not be a cancer driver because it was too common in general population. SNV somatic calls from either MuTect or Varscan in tier 1-3 genes, and all indel calls regardless of tier, were manually reviewed in IGV and the UCSC Genome Browser [22] to inspect supporting alignment quality in the BAM files and alignability of the genomic region in the hg19 human genome assembly, paying attention to whether a variant call was located in a short tandem repeat or a low-complexity sequence region [23], a region with self-homology/duplication in the reference genome, or a region of low alignability according to the GEM track from ENCODE/CRG [24]. Uncertain calls were manually rejected at this step. If RNA-Seq data was available, it was also used in the manual review process to see if the mutation appeared in the transcript. Observation of expression of the somatic mutant allele was noted in the returned genomics findings, but absence of such expression did not necessarily disqualify the call unless high overall abundance was observed, as the mutant allele may not have been observed in RNA due to under-sampling.

As part of routine post-sequencing QC of all WES and WGS data, we examined the factors given in Additional file 1: Table S6, and additionally: total number of variant calls (germline and somatic), the distribution of mutation types for all somatic calls (C>A, C>G, C>T, T>A, T>C, T>G; see “Mutation nomenclature” sub-section in Methods), transition/transversion ratio and ratio of heterozygous to homozygous alternate allele germline calls, distribution of allelic fractions as a function of sequencing depth (by tier 1-4 genes as well as synonymous versus non-synonymous/splice-site-altering variants), and the CNA profile. The first two items, in combination with cross-referencing the somatic calls with their population germline allele frequencies, allowed us to screen for contamination in the tumor, as the addition of DNA from an unrelated individual to the tumor DNA would cause a dramatic increase in low-level variants called as somatic that actually correspond to common population SNPs from the contaminating individual. The last two items allowed us to identify cases where the tumor DNA specimen did not contain plausible somatic alterations because the tumor content of specimen was low or the sample was from a tumor with a low mutation rate (in the latter case, under-sampling of genomic regions on WES, or presence of alterations in non-coding regions outside of the WES target regions, would further confound distinguishing the two possibilities).

Variants from targeted panel sequencing were called as described in the targeted panel experimental methods section, exported as VCFs, and loaded into a custom MySQL database in the same way as above Illumina workflow. The somatic variants were identified by subtracting the normal call set from the tumor call set. All resulting somatic variants were manually reviewed in IGV. If both targeted panel and WES sequencing were available, concordance analysis was done to ensure the two assays agreed in their somatic variant calls on regions covered by both assay designs, and this concordance was noted in the returned genomic findings.

*Copy number alterations.* Copy number alterations were identified using the saasCNV pipeline (<https://zhangz05.u.hpc.mssm.edu/saasCNV/index.htm>) [25] implemented in R. Briefly, from WES (and separately, from array) data, the pipeline begins with input heterozygous SNV calls from the normal control sample, and gathers their sequencing depth (for arrays, probe intensity LRR) and allelic fraction (for arrays, BAF) in the normal and tumor samples. Joint circular binary segmentation is performed on the two signals: the log-ratio of depth (intensities) and the log-ratio of mirrored allelic fractions (mBAFs) in tumor versus normal. Identified segments are then classified according to loss, copy-neutral LOH, gain, normal (no change), or undecided categories.

For SNP microarray data, genotypes were called using GenomeStudio and input to saasCNV. For WES data, because the GATK variant calling workflow combines normal and tumor as a cohort, heterozygous variants in the normal samples are implicitly force-called by HaplotypeCaller in the tumor sample, yielding their depth-by-allele in both normal and tumor in the output VCF. Variant calls that pass VQSR, have a 0/1 genotype in normal, have a mapping quality (MQ) > 30, and have non-zero depth in tumor, were input into saasCNV.

As every patient with array data also had WES, output of the WES-based CNA pipeline was examined for concordance with output of the array-based CNA data by comparing the high-level segmentation profiles (which will identify matching aneuploidy events) and gene-level CNA calls for cancer-relevant genes. In case of gene-level discordance, the raw data was manually reviewed.

*Identification of gene fusions.* To discover gene fusion events, we developed a computational pipeline to predict fusions from short-read RNA-Seq data. We employ a consensus scheme to detect putative fusions in our dataset. Briefly, our pipeline utilizes the following prediction algorithms – Chimerascan (version 0.4.5), FusionCatcher (version 0.99.3b), FusionMap (release 03/31/2015) and TopHat-Fusion (version 2.0.13) to compile a list of potential fusions in the data. Also, OncoFuse (version 1.0.9) is used to predict the probability of a fusion to be a driver and Chimera (version 1.10.0) to annotate the fusion product. The output from all algorithms is collated to list any fusions predicted by two or more algorithms. Additionally, all predicted fusion events are cross-referenced against the Mitelman database of known fusions to discover recurrent fusions. Furthermore, each fusion call is reviewed by a cancer biologist to ascertain accuracy and determine actionability.

*RNA-Seq gene expression analysis.* Gene expression was quantified from RNA-Seq data using the RSEM method [26]. TCGA breast cancer and colon cancer RNA-Seq expression data were downloaded from TCGA data portal (<https://tcga-data.nci.nih.gov/tcga/>). For patients with sequence data from both tumor and adjacent normal tissues, the log2 transformed tumor-normal gene expression ratio was used to represent differential gene expression. For breast cancer and colon cancers with only frozen tumor samples available, RNA-Seq gene expression data was first batch-effect adjusted against the downloaded TCGA corresponding data sets using empirical Bayes methods [27], and then normalized against the corresponding TCGA expression dataset using interquartile normalization. Subsequently, a z-score was computed for each gene using the TCGA normal tissue expression data as references.

### Tumor purity estimation

Only samples with WES data were used for post-NGS tumor purity estimation because of the large number of variant calls available in WES data. Allelic fractions of all somatic variant calls from WES were plotted and manually reviewed, paying special attention to known driver mutations (in combination with their CNA status, which is a confounding factor), as known driver mutations would be expected to occur early in the tumorigenesis process. This data was manually compared with distributions of allelic fractions of germline heterozygous variants in the tumor sample (based on WES and array CNA analysis), because in somatic loss-of-heterozygosity and copy-number-loss regions, these distributions would shift from a median of 0.5 that was seen in the normal sample. Thus, the degree of the shift corresponds to the percentage of DNA in the mixture containing that somatic CNA. The upper bound estimate of tumor content was generally in agreement between the somatic allelic fraction analysis and the analysis from CNA data. In two cases, due to lack of detected CNA and low number of somatic variant calls, tumor purity estimates could not be assigned.

### Functional validation of EGFR p.D587H mutation

*Construction of Mutants.* The site-directed mutagenesis reaction on EGFR-GFP was performed (Bioinnovatise, Rockville, MD) using high-fidelity DNA polymerase (Pfu, Agilent) and a temperature cycler. The basic procedure utilized ~50ng of wildtype plasmid as template with 125ng of two synthetic oligonucleotide primers containing the desired mutation. Subsequently, 2µl of mutagenesis reaction was transformed into supercompetent cells. After transformation, mutant plasmid DNA isolated from single colony was verified by DNA sequencing.

*Cell Culture and Transfection.* HEK293 cells were cultured in DMEM containing antibiotics and 10% fetal bovine serum in 5% CO_2_. Cells were plated in 6 well plates at a density of 300,000 cells/well. On the following day, cells were transfected with 4µg of either EGFR-GFP, EGFR(D587H)-GFP, or EGFR(L858R)-GFP using Lipofectamine2000 (Life Technologies).

*Western Blotting.* Cells were washed with PBS followed by addition of 50µl of ice-cold NE-PER (Pierce) with protease and phosphatase inhibitors (Roche) and scraping. Lysis was performed for 30 minutes on ice and then lysates were spun down at 12000g at 4C and supernatant was collected. Protein determination was performed using the Bradford assay (Biorad). 20µg of total protein was added per well and electrophoresed on 8% SDS-PAGE gels. After transfer to PVDF membranes (GE Healthcare), was followed by blocking in 3%BSA in TBST for 1 hour. Incubation with primary antibody (1:1000 in blocking buffer) was done at 4C overnight. Antibodies to EGFR and phosphotyrosine were purchased from Cell Signaling Technology. Incubation with secondary antibody anti-rabbit HRP or anti-mouse HRP (Cell Signaling Technology) was for 45 minutes and detection was performed using ECL reagent (GE healthcare).

# Supplementary Table Legends

**Supplementary Table S1.**  **Detailed per-patient info on sub-cohort of 46 patients on whom genomics data was available for analysis.** Counts of these patients by various characteristics are in Table 1.

**Supplementary Table S2.**  **All assays that were attempted (including failures) and the specimens on whose extractions they were carried out.** Anatomic specimen sites, preservation or collection methods, and ischemia time are given when known. The “cancer type” column shows the diagnosis of the tumor being assayed, to disambiguate cases where a patient has tumor specimens from different cancers; thus, no cancer type is given for normal specimens. The “assay success” column shows if the assay yielded data suitable for completion of primary genomics analysis, but does not mean that usable findings resulted from this assay (e.g. targeted panel sequencing could yield high-quality data, but no somatic variants identified; this would still be marked “successful” in this column because the assay did technically work, even though biologically no somatic mutations were present in the interrogated regions). The “results used” column shows whether claims were made in the returned genomics findings document that were derived from data from that assay (including if data from one assay validated results from another); if it says “no”, then assay data was either deemed too uninformative to be returned or there were technical issues with the assay (see the notes columns). The “multiple tries” column shows if any step in the sequencing pipeline had to be repeated, causing delays. NK: not known.

**Supplementary Table S3.** **Genes with germline mutations associated with breast cancer risk.**

**Supplementary Table S4.** **Curated cancer signaling pathways.** Listed are cancer signaling pathways, gene symbol and Entrez Gene ID.

**Supplementary Table S5.** **Cancer-relevant somatic mutations returned to each of the 45 patients who received genomics findings.** For patients with multiple tumors assayed, the mutation list and count given is the union of all mutations across all tumors.

**Supplementary Table S6.** **Primary analysis QC statistics on WES and WGS data produced.** Only statistics on the subset of 46 patients in Table 1 and Supplementary Table S1 who had WES or WGS data is given (N=42).  For QC, statistics as output by Picard are given for the target regions. The column “had repair” indicates whether FFPE repair was done on the DNA prior to WGS library preparation, as FFPE repair was sometimes applied to non-FFPE samples. The columns “num FCs used” and “num lanes used” refer to the number of flowcells and lanes, respectively, in which the libraries prepped from the given specimen appear; because some patients were multiplexed with others, libraries from another patient/sample could be present in the same lane, thus these columns do not necessarily mean that all reads from a lane/flowcell went to the indicated sample. The column “multiplexing strategy” indicates the *effective* multiplexing applied, i.e. how many flowcells’ worth of reads were essentially obtained. For example, libraries from patients P0018, P0032, and P0035 (N=3) were multiplexed together and run on 3 RR flowcells, thus each patient gets *effectively* 1 RR flowcell and the strategy is given as “1 RR FC” for that reason (note the sample pair for each of these 3 patients contains ~300M clusters, as is expected for a *single* RR flowcell). However, “num FCs used” is given as 3, indicating that data from 3 flowcells (3 separate sequencing runs) had to be pooled for each sample for each patient. FC: flowcell, RR: Rapid Run mode, HO: High Output mode.

**Supplementary Table S7. Summary of QC statistics on targeted panel sequencing data produced.** Only statistics on the subset of 46 patients in Table 1 and Supplementary Table S1 who had targeted panel sequencing data is given (N=42). QC statistics are as reported by TorrentSuite software (v4.0) from Life Technologies. For the column showing number of samples per run, only samples from libraries that were successful in that run (which we define as yielding >200X mean depth) are counted, in order to more clearly show how multiplexing influences depth in routine settings.

**Supplementary Table S8. Summary of sequencing protocols used and QC statistics on RNA-Seq data produced.** Only statistics on the subset of 46 patients in Table 1 and Supplementary Table S1 who had RNA-Seq data is given (N=18).  RR: Rapid Run mode; HO: High Output mode; RIN: RNA Integrity Number; ERCC: External RNA Controls Consortium.

**Supplementary Table S9. Concordance of somatic mutations called by WES versus by targeted panel sequencing.**

**Supplementary Table S10. Somatic mutation signature in patient P0011.**

**Supplementary Table S11. Tumor purities estimated solely based on NGS data.**

# References

1. Alexandrov LB, Nik-Zainal S, Wedge DC, Aparicio SA, Behjati S, Biankin AV et al. Signatures of mutational processes in human cancer. Nature. 2013;500(7463):415-21. doi:10.1038/nature12477.

2. Lawrence MS, Stojanov P, Polak P, Kryukov GV, Cibulskis K, Sivachenko A et al. Mutational heterogeneity in cancer and the search for new cancer-associated genes. Nature. 2013;499(7457):214-8. doi:10.1038/nature12213.

3. Helleday T, Eshtad S, Nik-Zainal S. Mechanisms underlying mutational signatures in human cancers. Nature reviews Genetics. 2014;15(9):585-98. doi:10.1038/nrg3729.

4. Schnitzler M, Hasskarl J, Egger M, Bertz H, Finke J. Successful treatment of severe acute intestinal graft-versus-host resistant to systemic and topical steroids with alemtuzumab. Biology of blood and marrow transplantation : journal of the American Society for Blood and Marrow Transplantation. 2009;15(8):910-8. doi:10.1016/j.bbmt.2009.04.002.

5. Price RL, Chiocca EA. Modeling cytomegalovirus infection in mouse tumor models. Frontiers in oncology. 2015;5:61. doi:10.3389/fonc.2015.00061.

6. Schumacher TN, Schreiber RD. Neoantigens in cancer immunotherapy. Science (New York, NY). 2015;348(6230):69-74. doi:10.1126/science.aaa4971.

7. Jiang L, Schlesinger F, Davis CA, Zhang Y, Li R, Salit M et al. Synthetic spike-in standards for RNA-seq experiments. Genome research. 2011;21(9):1543-51. doi:10.1101/gr.121095.111.

8. Linderman MD, Brandt T, Edelmann L, Jabado O, Kasai Y, Kornreich R et al. Analytical validation of whole exome and whole genome sequencing for clinical applications. BMC medical genomics. 2014;7:20. doi:10.1186/1755-8794-7-20.

9. McKenna A, Hanna M, Banks E, Sivachenko A, Cibulskis K, Kernytsky A et al. The Genome Analysis Toolkit: a MapReduce framework for analyzing next-generation DNA sequencing data. Genome research. 2010;20(9):1297-303. doi:10.1101/gr.107524.110.

10. DePristo MA, Banks E, Poplin R, Garimella KV, Maguire JR, Hartl C et al. A framework for variation discovery and genotyping using next-generation DNA sequencing data. Nature genetics. 2011;43(5):491-8. doi:10.1038/ng.806.

11. Van der Auwera GA, Carneiro MO, Hartl C, Poplin R, Del Angel G, Levy-Moonshine A et al. From FastQ data to high confidence variant calls: the Genome Analysis Toolkit best practices pipeline. Current protocols in bioinformatics / editoral board, Andreas D Baxevanis [et al]. 2013;11(1110):11.0.1-.0.33. doi:10.1002/0471250953.bi1110s43.

12. Cibulskis K, Lawrence MS, Carter SL, Sivachenko A, Jaffe D, Sougnez C et al. Sensitive detection of somatic point mutations in impure and heterogeneous cancer samples. Nature biotechnology. 2013;31(3):213-9. doi:10.1038/nbt.2514.

13. Forbes SA, Beare D, Gunasekaran P, Leung K, Bindal N, Boutselakis H et al. COSMIC: exploring the world's knowledge of somatic mutations in human cancer. Nucleic acids research. 2015;43(Database issue):D805-11. doi:10.1093/nar/gku1075.

14. Sherry ST, Ward MH, Kholodov M, Baker J, Phan L, Smigielski EM et al. dbSNP: the NCBI database of genetic variation. Nucleic acids research. 2001;29(1):308-11.

15. Koboldt DC, Zhang Q, Larson DE, Shen D, McLellan MD, Lin L et al. VarScan 2: somatic mutation and copy number alteration discovery in cancer by exome sequencing. Genome research. 2012;22(3):568-76. doi:10.1101/gr.129684.111.

16. Cingolani P, Platts A, Wang le L, Coon M, Nguyen T, Wang L et al. A program for annotating and predicting the effects of single nucleotide polymorphisms, SnpEff: SNPs in the genome of Drosophila melanogaster strain w1118; iso-2; iso-3. Fly. 2012;6(2):80-92. doi:10.4161/fly.19695.

17. Cunningham F, Amode MR, Barrell D, Beal K, Billis K, Brent S et al. Ensembl 2015. Nucleic acids research. 2015;43(Database issue):D662-9. doi:10.1093/nar/gku1010.

18. Ciriello G, Miller ML, Aksoy BA, Senbabaoglu Y, Schultz N, Sander C. Emerging landscape of oncogenic signatures across human cancers. Nature genetics. 2013;45(10):1127-33. doi:10.1038/ng.2762.

19. Kandoth C, McLellan MD, Vandin F, Ye K, Niu B, Lu C et al. Mutational landscape and significance across 12 major cancer types. Nature. 2013;502(7471):333-9. doi:10.1038/nature12634.

20. Fu W, O'Connor TD, Jun G, Kang HM, Abecasis G, Leal SM et al. Analysis of 6,515 exomes reveals the recent origin of most human protein-coding variants. Nature. 2013;493(7431):216-20. doi:10.1038/nature11690.

21. Abecasis GR, Auton A, Brooks LD, DePristo MA, Durbin RM, Handsaker RE et al. An integrated map of genetic variation from 1,092 human genomes. Nature. 2012;491(7422):56-65. doi:10.1038/nature11632.

22. Rosenbloom KR, Armstrong J, Barber GP, Casper J, Clawson H, Diekhans M et al. The UCSC Genome Browser database: 2015 update. Nucleic acids research. 2015;43(Database issue):D670-81. doi:10.1093/nar/gku1177.

23. Benson G. Tandem repeats finder: a program to analyze DNA sequences. Nucleic acids research. 1999;27(2):573-80.

24. Derrien T, Estelle J, Marco Sola S, Knowles DG, Raineri E, Guigo R et al. Fast computation and applications of genome mappability. PloS one. 2012;7(1):e30377. doi:10.1371/journal.pone.0030377.

25. Zhang Z, Hao K. SAAS-CNV: A Joint Segmentation Approach on Aggregated and Allele Specific Signals for the Identification of Somatic Copy Number Alterations with Next-Generation Sequencing Data. PLoS computational biology. 2015;11(11):e1004618. doi:10.1371/journal.pcbi.1004618.

26. Li B, Dewey CN. RSEM: accurate transcript quantification from RNA-Seq data with or without a reference genome. BMC bioinformatics. 2011;12:323. doi:10.1186/1471-2105-12-323.

27. Johnson WE, Li C, Rabinovic A. Adjusting batch effects in microarray expression data using empirical Bayes methods. Biostatistics (Oxford, England). 2007;8(1):118-27. doi:10.1093/biostatistics/kxj037.

28. Neph S, Kuehn MS, Reynolds AP, Haugen E, Thurman RE, Johnson AK et al. BEDOPS: high-performance genomic feature operations. Bioinformatics. 2012;28(14):1919-20. doi:10.1093/bioinformatics/bts277.
