# Supplementary material for: Development and clinical application of an integrative genomic approach to personalized cancer therapy
Source: Genome Med. 2016 Jun 1;8:62. doi: 10.1186/s13073-016-0313-0 (PMC4888213; doi:10.1186/s13073-016-0313-0)
Supplement: Supplementary file 3 — A decision tree approach to predict drug response based on genetic alterations. (PPTX 96 kb) [file 13073_2016_313_MOESM3_ESM.pptx]

## Slide 1
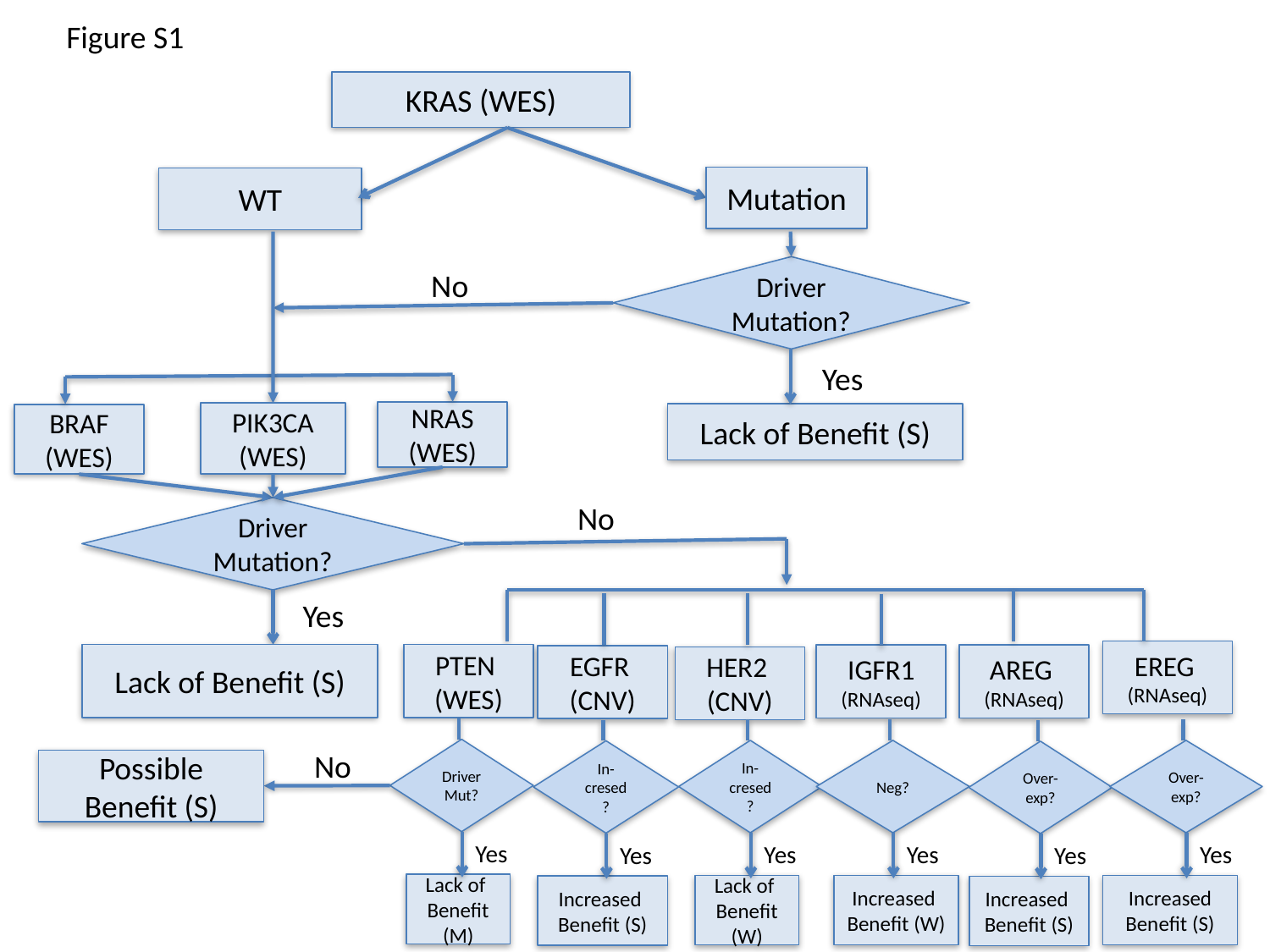

Figure S1
KRAS (WES)
Mutation
WT
Driver Mutation?
No
Yes
Lack of Benefit (S)
NRAS
(WES)
PIK3CA
(WES)
BRAF (WES)
No
Driver Mutation?
Yes
EREG
(RNAseq)
Lack of Benefit (S)
PTEN
(WES)
IGFR1
(RNAseq)
AREG
(RNAseq)
EGFR
(CNV)
HER2
(CNV)
Driver Mut?
Yes
Lack of
Benefit (M)
In-cresed?
Yes
Lack of
Benefit (W)
Neg?
Yes
Increased
Benefit (W)
Over-exp?
Yes
Increased
Benefit (S)
In-cresed?
Yes
Increased
Benefit (S)
Over-exp?
Yes
Increased
Benefit (S)
No
Possible Benefit (S)
