# Supplementary figures and images for: Development and clinical application of an integrative genomic approach to personalized cancer therapy
Source: Genome Med. 2016 Jun 1;8:62. doi: 10.1186/s13073-016-0313-0 (PMC4888213; doi:10.1186/s13073-016-0313-0)

## Slide 1
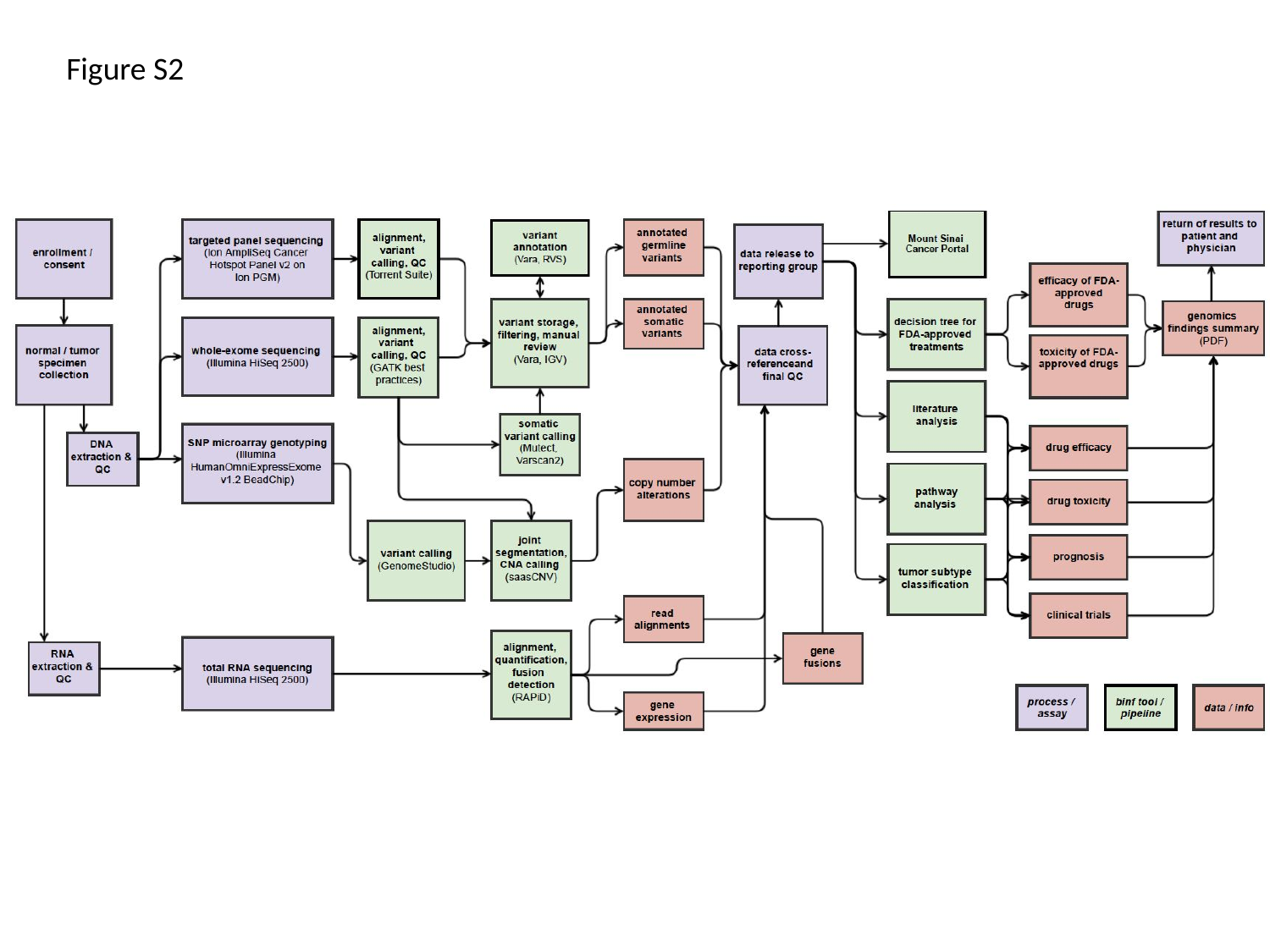

Figure S2

Supplement: Supplementary file 4 — Detailed workflow of an integrative genomic approach in personalized cancer therapy. (PPTX 303 kb) [file 13073_2016_313_MOESM4_ESM.pptx]

## Slide 1
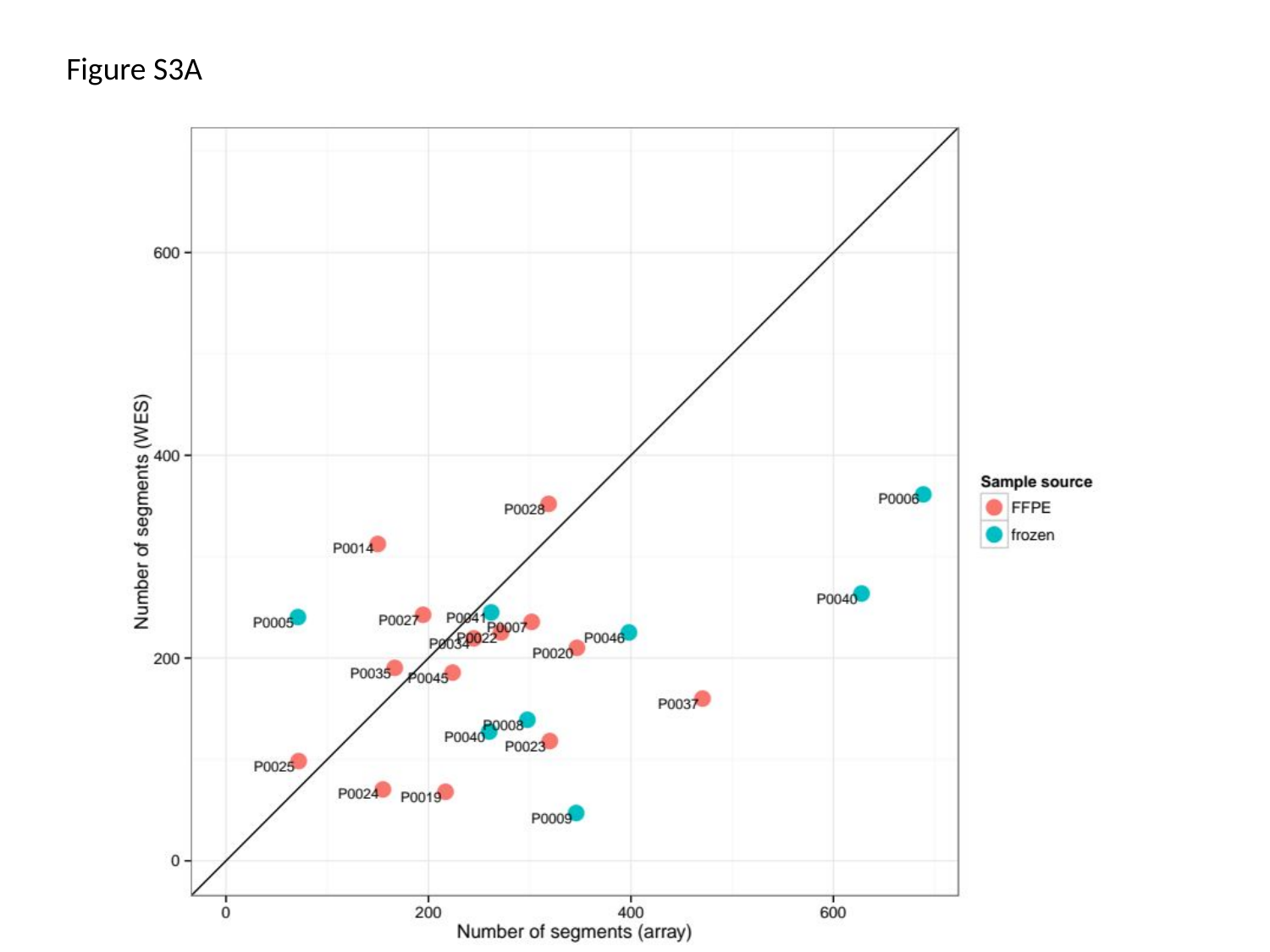

Figure S3A

Supplement: Supplementary file 6 — Comparison of somatic CNA segment properties (number and length) between joint segmentation calls by saasCNV (https://zhangz05.u.hpc.mssm.edu/saasCNV) from WES data versus array data. Only samples where both assays were run on same DNA extraction are shown. All segments are shown, including those classified as “normal” (no CNA) and “undecided” (unclear CNA change). Patient P0040 is shown twice, once for each of the two tumors assayed, though in both cases the same normal control data is used. “FFPE” and “frozen” in all plots refers to tissue source of tumor DNA. a Correlation of total segment number per tumor between WES and array assays. b Violin plot comparing the distribution of segment lengths between WES and array assays for each tumor. Boxplot within each violin plot shows median, 25th, and 75th quantiles. (ZIP 229 kb) [file 13073_2016_313_MOESM6_ESM.zip › Fig S3A.pptx]

## Slide 1
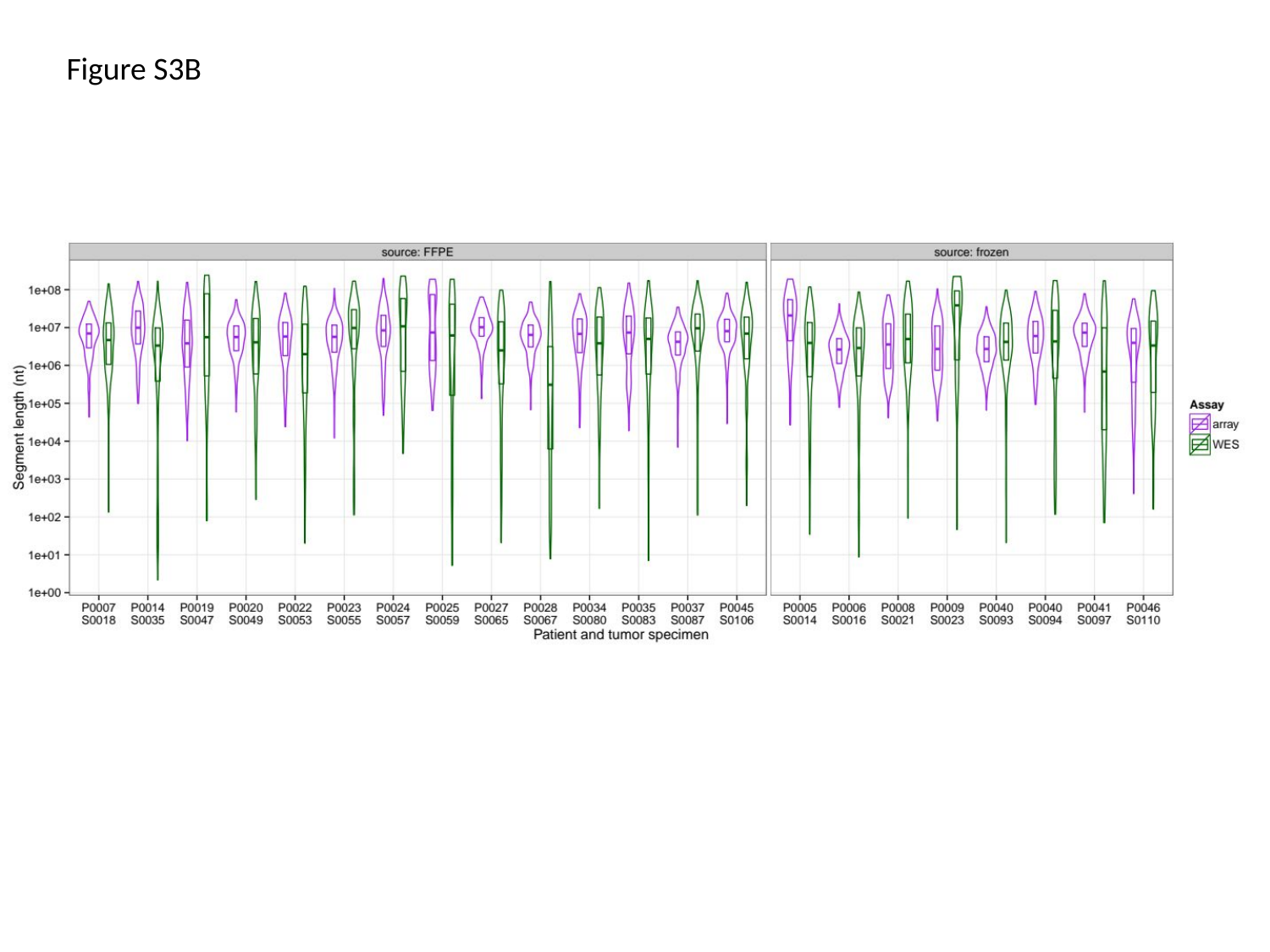

Figure S3B

Supplement: Supplementary file 6 — Comparison of somatic CNA segment properties (number and length) between joint segmentation calls by saasCNV (https://zhangz05.u.hpc.mssm.edu/saasCNV) from WES data versus array data. Only samples where both assays were run on same DNA extraction are shown. All segments are shown, including those classified as “normal” (no CNA) and “undecided” (unclear CNA change). Patient P0040 is shown twice, once for each of the two tumors assayed, though in both cases the same normal control data is used. “FFPE” and “frozen” in all plots refers to tissue source of tumor DNA. a Correlation of total segment number per tumor between WES and array assays. b Violin plot comparing the distribution of segment lengths between WES and array assays for each tumor. Boxplot within each violin plot shows median, 25th, and 75th quantiles. (ZIP 229 kb) [file 13073_2016_313_MOESM6_ESM.zip › Fig S3B.pptx]

## Slide 1
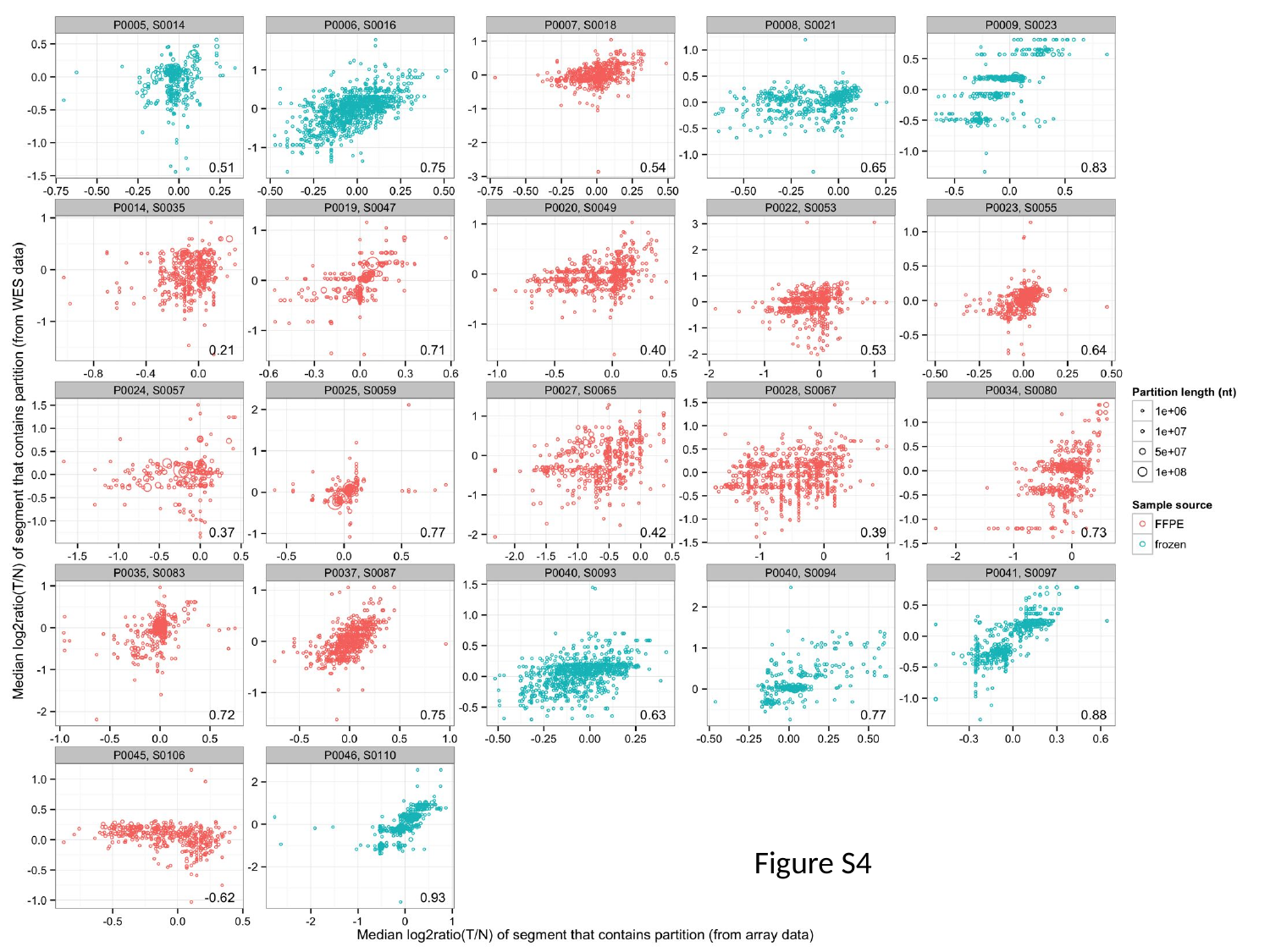

Figure S4

Supplement: Supplementary file 7 — Correlation of somatic CNA fold-change (median “log2ratio” segment statistic from saasCNV of tumor with respect to normal) between WES and array data. The genome was split into non-overlapping partitions such that each partition begins and ends on a CNA segment break from either assay, but no CNA segment breaks occur inside any partition (“partition” feature of “bedops” software tool v2.4.14, https://github.com/bedops/bedops). Thus, each partition overlaps exactly one segment from WES data and exactly one segment from array data. Each point is a partition, with plotted values taken from the pair of segments that overlap it. Lower right corner shows the weighed (by partition length) Pearson correlation between WES- and array-derived log2ratio values for partitions, computed using the “corr” function from R (v3.2.1) package “boot” (v1.3-17). “FFPE” and “frozen” in all plots refers to tissue source of tumor DNA. (PPTX 1114 kb) [file 13073_2016_313_MOESM7_ESM.pptx]

## Slide 1
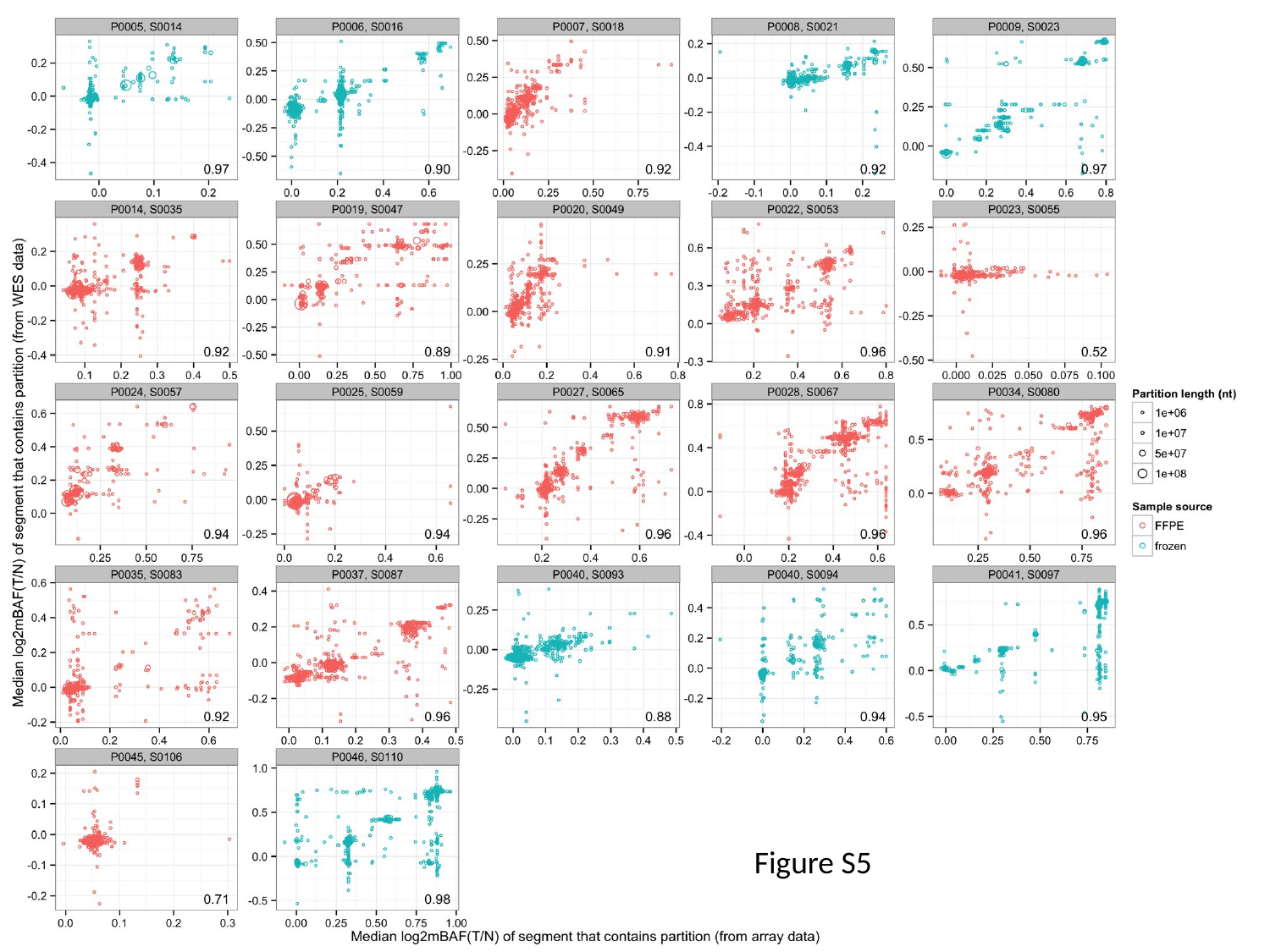

Figure S5

Supplement: Supplementary file 8 — Correlation of somatic CNA heterozygosity change (median “log2mBAF” segment statistic from saasCNV of tumor with respect to normal) between WES and array data. The same partitions are used as in Additional file 7: Figure S4, and weighed correlation in the lower right corner is also computed in the same way. (PPTX 981 kb) [file 13073_2016_313_MOESM8_ESM.pptx]

## Slide 1
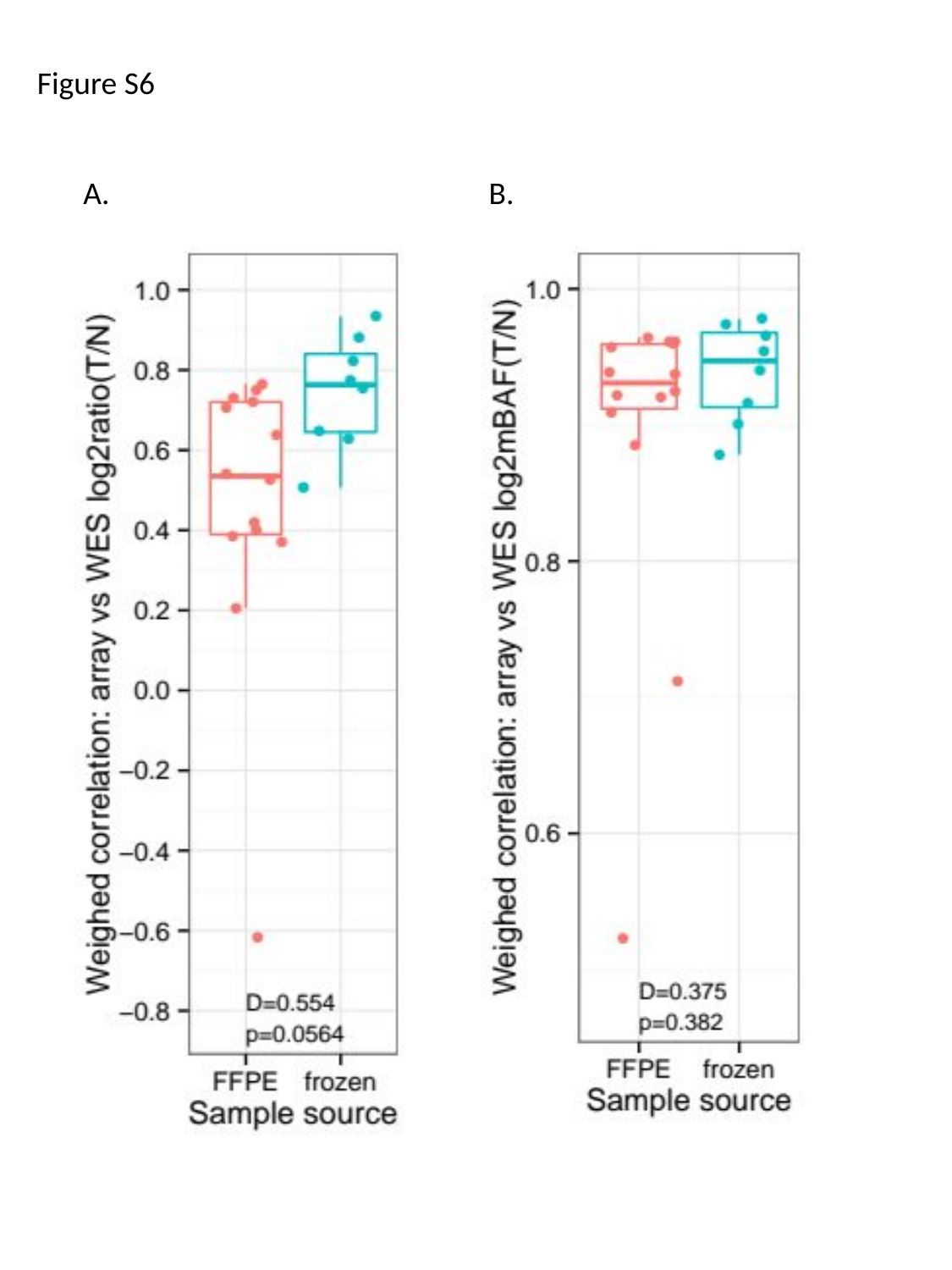

Figure S6
A.
B.

Supplement: Supplementary file 9 — Comparison of weighed correlations of log2ratio (a, data from Additional file 7: Figure S4) and log2mBAF (b, data from Additional file 8: Figure S5) for assays on FFPE- versus frozen-derived tumor DNA material. D statistic and p-value from 2-sided KS test are shown for FFPE- versus frozen-derived correlation distributions (see "Methods" for details on KS test). (PPTX 76 kb) [file 13073_2016_313_MOESM9_ESM.pptx]

## Slide 1
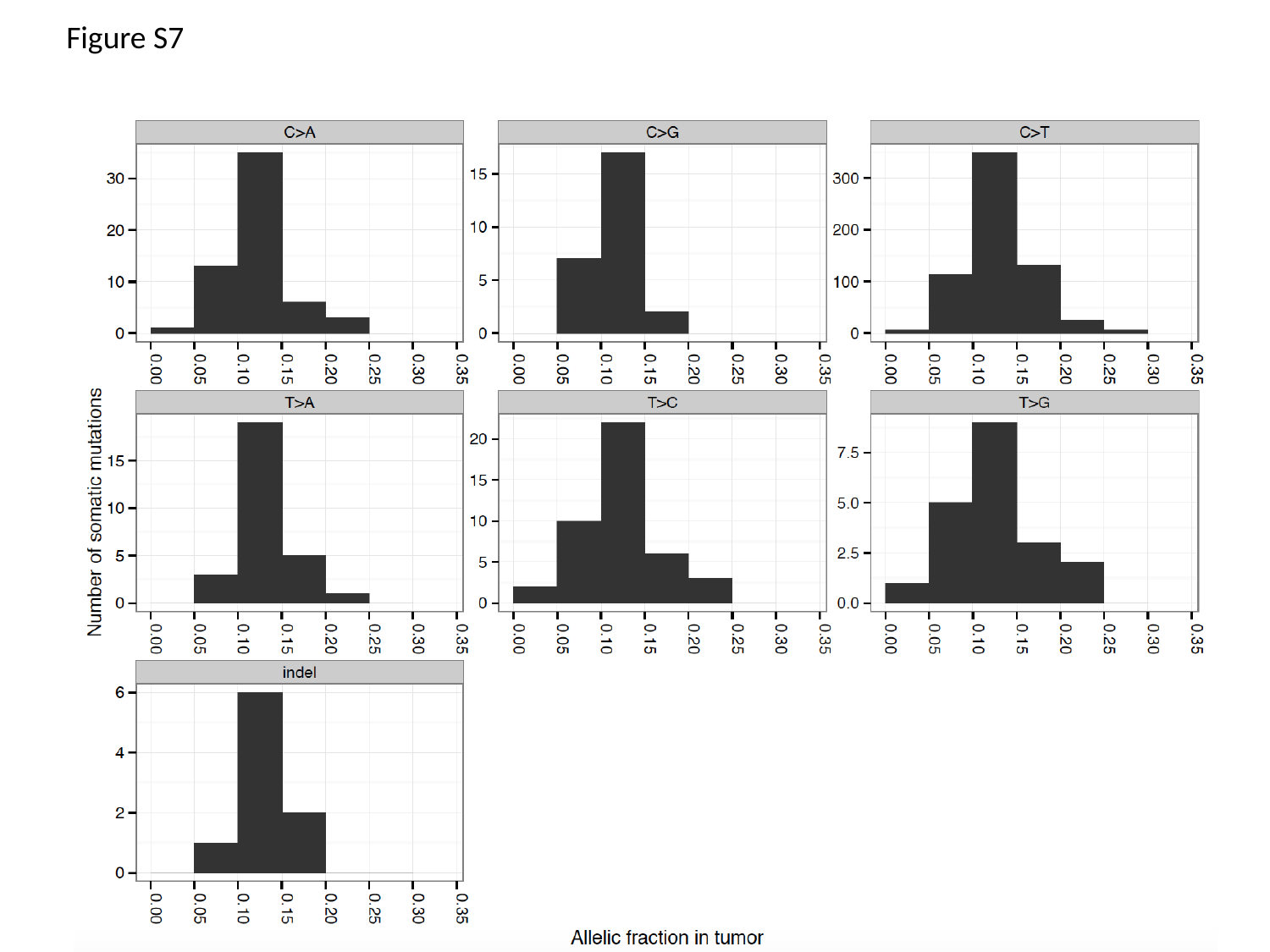

Figure S7

Supplement: Supplementary file 10 — Distribution of somatic mutation allelic fractions in patient P0011, by mutation type. (PPTX 191 kb) [file 13073_2016_313_MOESM10_ESM.pptx]

## Slide 1
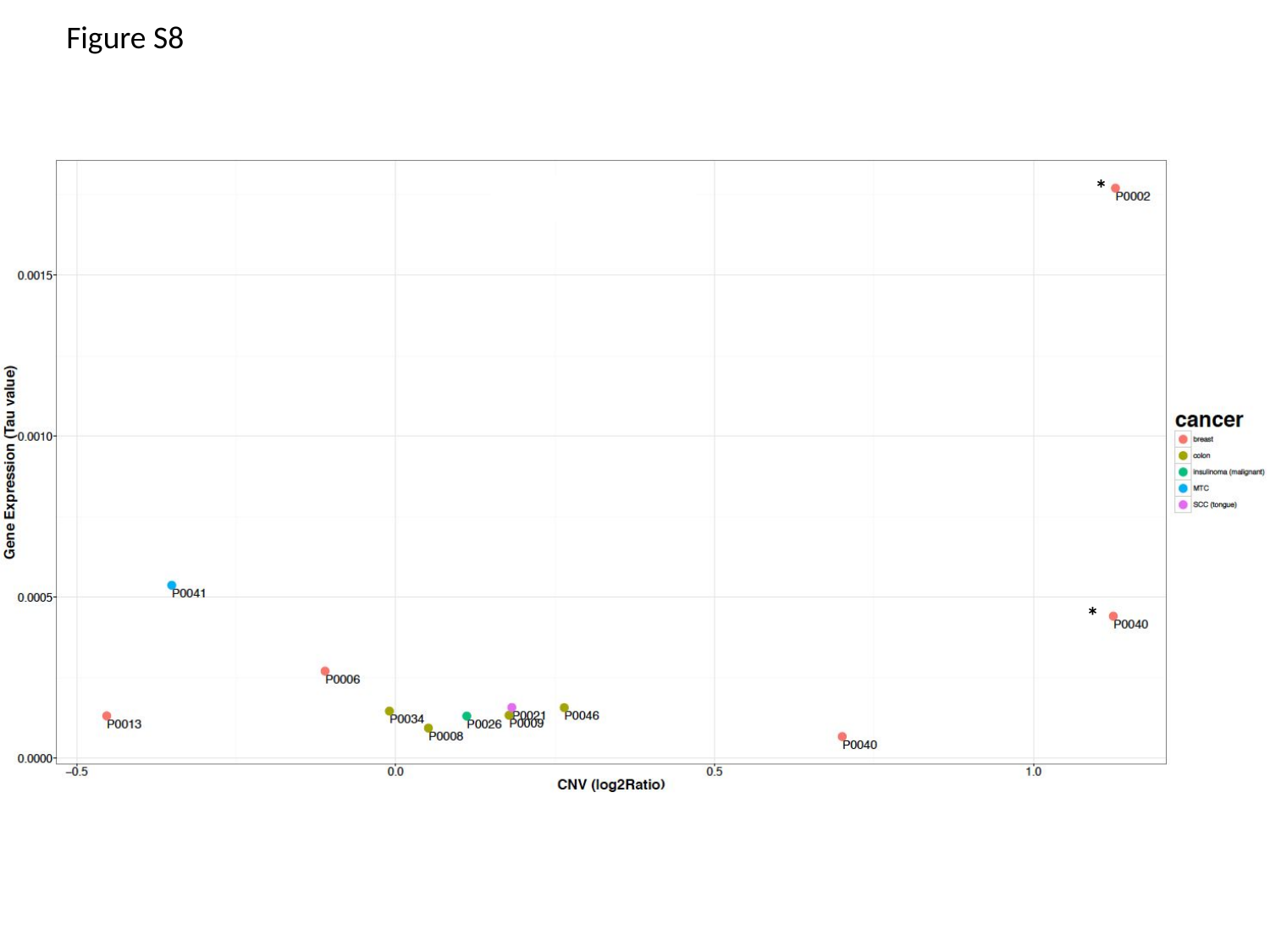

Figure S8
*
CNV (log2 ratio)
*

Supplement: Supplementary file 11 — A scatter plot of CCND1 gene expression versus log2 copy number ratio (tumor/normal). Each dot represents a patient tumor sample. Tumor types are color-coded. The two breast cancer patients where we reported CCND1 amplification are P0002 and P0040. (PPTX 112 kb) [file 13073_2016_313_MOESM11_ESM.pptx]

## Slide 1
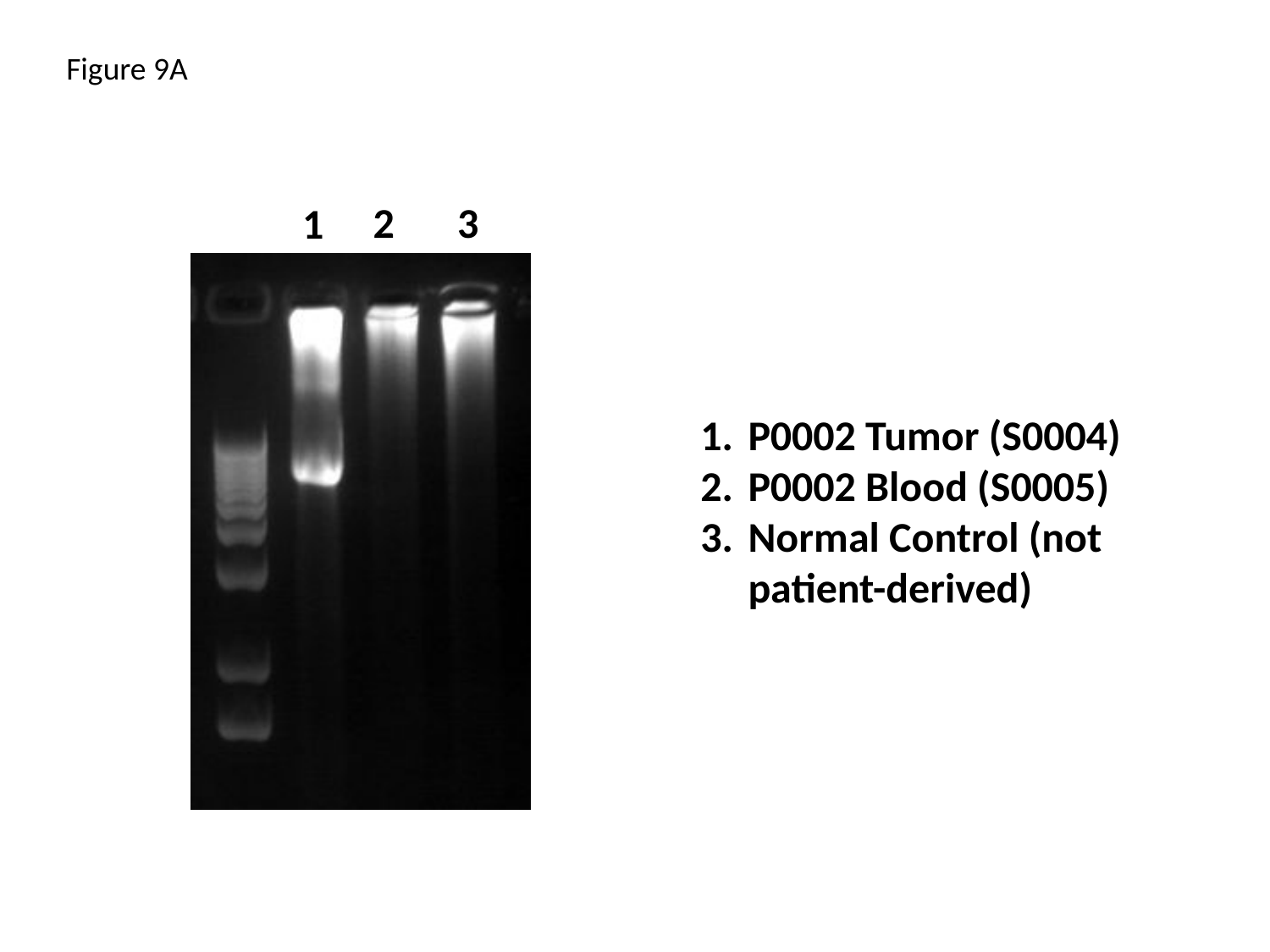

Figure 9A
2
3
1
P0002 Tumor (S0004)
P0002 Blood (S0005)
Normal Control (not patient-derived)

Supplement: Supplementary file 12 — CLPB-NADSYN1 gene fusion in patient P0002. a Long-range PCR confirms CLPB-NADSYN1 gene fusion. b Genomic breakpoint of CLPB-NADSYN1 gene fusion. (ZIP 150 kb) [file 13073_2016_313_MOESM12_ESM.zip › Fig S9A.pptx]
