# Supplementary material for: Development and clinical application of an integrative genomic approach to personalized cancer therapy
Source: Genome Med. 2016 Jun 1;8:62. doi: 10.1186/s13073-016-0313-0 (PMC4888213; doi:10.1186/s13073-016-0313-0)
Supplement: Supplementary file 5 — Example PDF document of genomic findings returned to patients and their treating physicians in this study. Each section of this document was taken from an actual returned document, but to protect patient privacy, different sections were taken from different patient documents and information identifying the patient and specimens was removed. (PDF 367 kb) [file 13073_2016_313_MOESM5_ESM.pdf]

Department of Genetics and Genomic Sciences
Icahn School of Medicine at Mount Sinai
1425 Madison Av, New York, NY 10029
Phone: (212) 241-6149

Personalized Cancer Research Findings

*** Caution – findings are investigational; limited by Federal law to investigational use only ***

PATIENT	SPECIMEN INFORMATION	
**PCT Patient #:**<br>P0000<br>**Age at consent:**<br>43<br>**Sex:**<br>Female	Primary Tumor Site:	colon
	Specimen Site:	1: blood<br>2: lung (left upper lobe)
	Specimen ID:	1: Mt. Sinai BRP ID # 1234G<br>2: Mt. Sinai BRP ID # 5678T
	Specimen Type:	1: blood draw<br>2: FFPE specimen from resection/open biopsy
	Specimen Received:	1: 2014/04/XX<br>2: 2014/05/XX
	Report Date:	
	Extractions:	1: gDNA from Specimen 1, done on 2014/04/XX<br>2: gDNA from Specimen 2, done on 2014/05/XX
	Assays:	1. Illumina HiSeq2500 whole-exome sequencing (WES) on Extractions 1 and 2.<br>2. Ion AmpliSeq Cancer Hotspot Panel v2 (Ion PGM sequencing) on Extractions 1 and 2.<br>3. Infinium Human OmniExpress Exome genotyping microarray on Extractions 1 and 2.

Disclaimer: Findings included in this report are not intended for use in clinical application such as prognosis estimation, risk assessment, or treatment decision-making. Findings are based on research assays and may not be validated by a clinical test. Caution should be used when interpreting the findings, as they are investigational. Some tests, drugs, and biomarkers identified in this report are for research purpose only and may not be approved by the FDA for requisite use or validated for that use. We do not endorse any product, physician, procedure or other information in such content, nor do these findings substitute for professional medical advice, diagnosis or treatment. Do not disregard professional medical advice or treatment because of anything you may read in this document.

Molecular Testing Results

1. Most relevant somatic mutations

Gene	Alteration	Validated?	Previously Observed in CRC	Previously Observed in Other Cancers	Pathway/Function
**Tier 1:** Genes known to be involved in CRC					
APC	p.R232*	-	yes	yes	APC gene participates in several cellular processes such as cell adhesion and migration, signal transduction, microtubule assembly and chromosome segregation. The main tumour suppressing function of APC resides in its capacity to properly regulate intracellular beta-catenin levels. APC is one of the genes with most frequent mutation in CRC. Recent studies have shown that the C-terminus of APC is involved in chromosomal stability at mitosis
	p.T1556fs*3	yes [a]	yes	yes	
ATM	p.C1502*	-	-	-	The protein encoded by this gene belongs to the PI3/PI4-kinase family. This protein is an important cell cycle checkpoint kinase that phosphorylates; thus, it functions as a regulator of a wide variety of downstream proteins, including tumor suppressor proteins p53 and BRCA1, checkpoint kinase CHK2, checkpoint proteins RAD17 and RAD9, and DNA repair protein NBS1. This protein and the closely related kinase ATR are thought to be master controllers of cell cycle checkpoint signaling pathways that are required for cell response to DNA damage and for genome stability. Mutations in this gene are associated with ataxia telangiectasia, an autosomal recessive disorder. Two transcript variants encoding different isoforms have been found for this gene.
BRAF	p.V600E	yes [a]	yes	yes	BRAF, a serine-threonine protein kinases is a central mediator in the MAP kinase signaling cascade and exert its effect predominantly through phosphorylation and activation of MEK. Approximately 8–15% of colorectal cancer (CRC) tumors harbor BRAF mutations
PTEN	splice site acceptor	yes [a]	yes	yes	This gene was identified as a tumor suppressor that is mutated in a large number of cancers at high frequency. The protein encoded this gene is a phosphatidylinositol-3,4,5-trisphosphate 3-phosphatase. It contains a tensin like domain as well as a catalytic domain similar to that of the dual specificity protein tyrosine phosphatases. Unlike most of the protein tyrosine phosphatases, this protein preferentially dephosphorylates phosphoinositide substrates. It negatively regulates intracellular levels of phosphatidylinositol-3,4,5-trisphosphate in cells and functions as a tumor suppressor by negatively regulating AKT/PKB signaling pathway.
SMAD4	p.R361H	yes [a]	yes	yes	This gene encodes a member of the Smad family of signal transduction proteins. Smad proteins are phosphorylated and activated by transmembrane serine-threonine receptor kinases in response to TGF-beta signaling. The product of this gene forms homomeric complexes and heteromeric complexes with other activated Smad proteins, which then accumulate in the nucleus and regulate the transcription of target genes. This protein binds to DNA and recognizes an 8-bp palindromic sequence (GTCTAGAC) called the Smad-binding element (SBE). The Smad proteins are subject to complex regulation by post-translational modifications. Mutations or deletions in this gene have been shown to result in pancreatic cancer, juvenile polyposis syndrome, and hereditary hemorrhagic telangiectasia syndrome.
**Tier 2:** Pan-cancer genes (known to be involved in several cancer types), excluding Tier 1					
None found.					
**Tier 3:** Genes known to be involved in other cancers, excluding Tier 1 and 2					
AFF4	p.L723fs	-	-	-	AFF4 (AF4/FMR2 family, member 4) is a protein-coding gene. Diseases associated with AFF4 include lymphoblastic leukemia. GO annotations related to this gene include sequence-specific DNA binding transcription factor activity.
AR	p.P378L	-	-	-	The androgen receptor gene is more than 90 kb long and codes for a protein that has 3 major functional domains: the N-terminal domain,

Disclaimer: Findings included in this report are not intended for use in clinical application such as prognosis estimation, risk assessment, or treatment decision-making. Findings are based on research assays and may not be validated by a clinical test. Caution should be used when interpreting the findings, as they are investigational. Some tests, drugs, and biomarkers identified in this report are for research purpose only and may not be approved by the FDA for requisite use or validated for that use. We do not endorse any product, physician, procedure or other information in such content, nor do these findings substitute for professional medical advice, diagnosis or treatment. Do not disregard professional medical advice or treatment because of anything you may read in this document.

					DNA-binding domain, and androgen-binding domain. The protein functions as a steroid-hormone activated transcription factor. Upon binding the hormone ligand, the receptor dissociates from accessory proteins, translocates into the nucleus, dimerizes, and then stimulates transcription of androgen responsive genes. This gene contains 2 polymorphic trinucleotide repeat segments that encode polyglutamine and polyglycine tracts in the N-terminal transactivation domain of its protein. Expansion of the polyglutamine tract causes spinal bulbar muscular atrophy (Kennedy disease). Mutations in this gene are also associated with complete androgen insensitivity (CAIS). Two alternatively spliced variants encoding distinct isoforms have been described.
HLF	p.P294H	-	-	-	This gene encodes a member of the proline and acidic-rich (PAR) protein family, a subset of the bZIP transcription factors. The encoded protein forms homodimers or heterodimers with other PAR family members and binds sequence-specific promoter elements to activate transcription. Chromosomal translocations fusing portions of this gene with the E2A gene cause a subset of childhood B-lineage acute lymphoid leukemias. Alternatively spliced transcript variants have been described, but their biological validity has not been determined.
MYCN	p.R398Q	-	-	similar [1]	This gene is a member of the MYC family and encodes a protein with a basic helix-loop-helix (bHLH) domain. This protein is located in the nucleus and must dimerize with another bHLH protein in order to bind DNA. Amplification of this gene is associated with a variety of tumors, most notably neuroblastomas.
MYO18B	p.R2108*	-	-	yes [1]	The protein encoded by this gene may regulate muscle-specific genes when in the nucleus and may influence intracellular trafficking when in the cytoplasm. The encoded protein functions as a homodimer and may interact with F actin. Mutations in this gene are associated with lung cancer.
NOTCH1	p.P1386T	-	-	-	This gene encodes a member of the Notch family. Members of this Type 1 transmembrane protein family share structural characteristics including an extracellular domain consisting of multiple epidermal growth factor-like (EGF) repeats, and an intracellular domain consisting of multiple, different domain types. Notch family members play a role in a variety of developmental processes by controlling cell fate decisions. The Notch signaling network is an evolutionarily conserved intercellular signaling pathway which regulates interactions between physically adjacent cells. In Drosophilia, notch interaction with its cell-bound ligands (delta, serrate) establishes an intercellular signaling pathway that plays a key role in development. Homologues of the notch-ligands have also been identified in human, but precise interactions between these ligands and the human notch homologues remain to be determined. This protein is cleaved in the trans-Golgi network, and presented on the cell surface as a heterodimer. This protein functions as a receptor for membrane bound ligands, and may play multiple roles during development.
NTRK3	p.R316C	-	-	-	This gene encodes a member of the neurotrophic tyrosine receptor kinase (NTRK) family. This kinase is a membrane-bound receptor that, upon neurotrophin binding, phosphorylates itself and members of the MAPK pathway. Signalling through this kinase leads to cell differentiation and may play a role in the development of proprioceptive neurons that sense body position. Mutations in this gene have been associated with medulloblastomas, secretory breast carcinomas and other cancers.
PTPRD	p.D19N	-	-	-	The protein encoded by this gene is a member of the protein tyrosine phosphatase (PTP) family. PTPs are known to be signaling molecules that regulate a variety of cellular processes including cell growth, differentiation, mitotic cycle, and oncogenic transformation. This PTP contains an extracellular region, a single transmembrane segment and two tandem intracytoplasmic catalytic domains, thus represents a receptor-type PTP. The extracellular region of this protein is composed of three Ig-like and eight fibronectin type III-like domains. Studies of the similar genes in chick and fly suggest the role of this PTP is in promoting neurite growth, and regulating neurons axon guidance. Multiple tissue specific alternatively spliced transcript variants of this gene have been reported.

Disclaimer: Findings included in this report are not intended for use in clinical application such as prognosis estimation, risk assessment, or treatment decision-making. Findings are based on research assays and may not be validated by a clinical test. Caution should be used when interpreting the findings, as they are investigational. Some tests, drugs, and biomarkers identified in this report are for research purpose only and may not be approved by the FDA for requisite use or validated for that use. We do not endorse any product, physician, procedure or other information in such content, nor do these findings substitute for professional medical advice, diagnosis or treatment. Do not disregard professional medical advice or treatment because of anything you may read in this document.

VCAN	p.T2689M	-	-	-	VCAN (versican) is a protein-coding gene. Diseases associated with VCAN include wagner syndrome, and vcan-related vitreoretinopathy. GO annotations related to this gene include hyaluronic acid binding and calcium ion binding.
**Tier 4:** Genes not known to be involved in any cancers, but which have somatic mutations that were previously observed					
ACTL7B	p.R295C	-	yes	-	The protein encoded by this gene is a member of a family of actin-related proteins (ARPs) which share significant amino acid sequence identity to conventional actins. Both actins and ARPs have an actin fold, which is an ATP-binding cleft, as a common feature. The ARPs are involved in diverse cellular processes, including vesicular transport, spindle orientation, nuclear migration and chromatin remodeling. This gene (ACTL7B), and related gene, ACTL7A, are intronless, and are located approximately 4 kb apart in a head-to-head orientation within the familial dysautonomia candidate region on 9q31. Based on mutational analysis of the ACTL7B gene in patients with this disorder, it was concluded that it is unlikely to be involved in the pathogenesis of dysautonomia. Unlike ACTL7A, the ACTL7B gene is expressed predominantly in the testis, however, its exact function is not known.
ART5	p.R145*	-	yes [1]	-	The protein encoded by this gene belongs to the ARG-specific ADP-ribosyltransferase family. Proteins in this family regulate the function of target proteins by attaching ADP-ribose to specific amino acid residues in their target proteins. The mouse homolog lacks a glycosylphosphatidylinositol-anchor signal sequence and is predicted to be a secretory enzyme. Transcript variants with different 5' UTRs, but encoding the same protein have been found for this gene.
BICC1	p.G436S	-	-	similar	This gene encodes an RNA-binding protein that is active in regulating gene expression during embryonic development. Mouse studies identified the corresponding protein to be under strict control during cell differentiation and to be a maternally provided gene product.
C10orf71	p.A1357V	-	yes [1]	-	C10orf71 (chromosome 10 open reading frame 71) is a protein-coding gene.
C1QTNF5	p.A195fs*8	-	yes [1]	yes [1]	The CTRP5 protein is a member of the C1q (see MIM 120550)/tumor necrosis factor (MIM 191160) superfamily, which shows diverse functions including cell adhesion and basement membrane components (Shapiro and Scherer, 1998). C1QTNF5 is mutant in late-onset retinal degeneration (LORD; MIM 605670).
CHD4	p.R1260C	-	-	yes	The product of this gene belongs to the SNF2/RAD54 helicase family. It represents the main component of the nucleosome remodeling and deacetylase complex and plays an important role in epigenetic transcriptional repression. Patients with dermatomyositis develop antibodies against this protein.
DMRTA1	p.R344Q	-	yes	-	DMRTA1 (DMRT-like family A1) is a protein-coding gene. GO annotations related to this gene include sequence-specific DNA binding and sequence-specific DNA binding transcription factor activity.
ESX1	p.L348V	-	-	yes [1]	ESX1 (ESX homeobox 1) is a protein-coding gene. Diseases associated with ESX1 include allan-herndon-dudley syndrome, and testicular germ cell tumor. GO annotations related to this gene include sequence-specific DNA binding and sequence-specific DNA binding transcription factor activity.
LRRC7	p.K228M	-	-	yes [1]	LRRC7 (leucine rich repeat containing 7) is a protein-coding gene.
METTL21A	p.R179W	-	-	yes [1]	METTL21A (methyltransferase like 21A) is a protein-coding gene. Diseases associated with METTL21A include hepatocellular carcinoma. GO annotations related to this gene include protein-lysine N-methyltransferase activity.
NRXN1	p.N190T	-	-	yes [1]	Neurexins are a family of proteins that function in the vertebrate nervous system as cell adhesion molecules and receptors. They are encoded by several unlinked genes of which two, NRXN1 and NRXN3, are among the largest known human genes. Three of the genes (NRXN1-3) utilize two alternate promoters and include numerous alternatively spliced exons to generate thousands of distinct mRNA transcripts and protein isoforms. The majority of transcripts are produced from the upstream promoter and encode alpha-neurexin isoforms; a much smaller number of transcripts are produced from the downstream promoter and encode beta-neurexin isoforms. The alpha-neurexins contain epidermal growth factor-like (EGF-like) sequences

Disclaimer: Findings included in this report are not intended for use in clinical application such as prognosis estimation, risk assessment, or treatment decision-making. Findings are based on research assays and may not be validated by a clinical test. Caution should be used when interpreting the findings, as they are investigational. Some tests, drugs, and biomarkers identified in this report are for research purpose only and may not be approved by the FDA for requisite use or validated for that use. We do not endorse any product, physician, procedure or other information in such content, nor do these findings substitute for professional medical advice, diagnosis or treatment. Do not disregard professional medical advice or treatment because of anything you may read in this document.

					and laminin G domains, and have been shown to interact with neurexophilins. The beta-neurexins lack EGF-like sequences and contain fewer laminin G domains than alpha-neurexins.
NUP50	p.T123A	-	-	yes	The nuclear pore complex is a massive structure that extends across the nuclear envelope, forming a gateway that regulates the flow of macromolecules between the nucleus and the cytoplasm. Nucleoporins are the main components of the nuclear pore complex in eukaryotic cells. The protein encoded by this gene is a member of the FG-repeat containing nucleoporins that functions as a soluble cofactor in importin-alpha:beta-mediated nuclear protein import. Pseudogenes of this gene are found on chromosomes 5, 6, and 14. Two transcript variants encoding different isoforms have been found for this gene.
OR1S2	p.T155I	-	-	yes [1]	Olfactory receptors interact with odorant molecules in the nose, to initiate a neuronal response that triggers the perception of a smell. The olfactory receptor proteins are members of a large family of G-protein-coupled receptors (GPCR) arising from single coding-exon genes. Olfactory receptors share a 7-transmembrane domain structure with many neurotransmitter and hormone receptors and are responsible for the recognition and G protein-mediated transduction of odorant signals. The olfactory receptor gene family is the largest in the genome. The nomenclature assigned to the olfactory receptor genes and proteins for this organism is independent of other organisms.
OR2L8	p.G212A	-	-	yes [1]	Olfactory receptors interact with odorant molecules in the nose, to initiate a neuronal response that triggers the perception of a smell. The olfactory receptor proteins are members of a large family of G-protein-coupled receptors (GPCR) arising from single coding-exon genes. Olfactory receptors share a 7-transmembrane domain structure with many neurotransmitter and hormone receptors and are responsible for the recognition and G protein-mediated transduction of odorant signals. The olfactory receptor gene family is the largest in the genome. The nomenclature assigned to the olfactory receptor genes and proteins for this organism is independent of other organisms.
OR2T3	p.Q6K	-	-	similar [1]	Olfactory receptors interact with odorant molecules in the nose, to initiate a neuronal response that triggers the perception of a smell. The olfactory receptor proteins are members of a large family of G-protein-coupled receptors (GPCR) arising from single coding-exon genes. Olfactory receptors share a 7-transmembrane domain structure with many neurotransmitter and hormone receptors and are responsible for the recognition and G protein-mediated transduction of odorant signals. The olfactory receptor gene family is the largest in the genome. The nomenclature assigned to the olfactory receptor genes and proteins for this organism is independent of other organisms.
OR52E8	p.R128H	-	-	similar	Olfactory receptors interact with odorant molecules in the nose, to initiate a neuronal response that triggers the perception of a smell. The olfactory receptor proteins are members of a large family of G-protein-coupled receptors (GPCR) arising from single coding-exon genes. Olfactory receptors share a 7-transmembrane domain structure with many neurotransmitter and hormone receptors and are responsible for the recognition and G protein-mediated transduction of odorant signals. The olfactory receptor gene family is the largest in the genome. The nomenclature assigned to the olfactory receptor genes and proteins for this organism is independent of other organisms.
PDPR	p.T29A	-	-	yes [1]	PDPR (pyruvate dehydrogenase phosphatase regulatory subunit) is a protein-coding gene. GO annotations related to this gene include oxidoreductase activity and aminomethyltransferase activity.
PLIN4	p.V903M	-	yes	yes	Members of the perilipin family, such as PLIN4, coat intracellular lipid storage droplets
PRB2	p.A235P	-	-	yes [1]	PRB2 (proline-rich protein BstNI subfamily 2) is a protein-coding gene.
PRDM9	p.R786S	-	yes	yes	The PR domain is a protein-protein interaction module of about 100 amino acids. PR domain-containing proteins, such as PRDM9, are often involved in transcriptional regulation (Jiang and Huang, 2000).
SLCO6A1	p.A322T	-	yes	-	SLCO6A1 (solute carrier organic anion transporter family, member

Disclaimer: Findings included in this report are not intended for use in clinical application such as prognosis estimation, risk assessment, or treatment decision-making. Findings are based on research assays and may not be validated by a clinical test. Caution should be used when interpreting the findings, as they are investigational. Some tests, drugs, and biomarkers identified in this report are for research purpose only and may not be approved by the FDA for requisite use or validated for that use. We do not endorse any product, physician, procedure or other information in such content, nor do these findings substitute for professional medical advice, diagnosis or treatment. Do not disregard professional medical advice or treatment because of anything you may read in this document.

					6A1) is a protein-coding gene. GO annotations related to this gene include transporter activity.
SLFN11	p.R436W	-	-	yes [1]	Interferon (IFN)-induced antiviral protein which acts as an inhibitor of retrovirus protein synthesis. Specifically abrogates the production of retroviruses such as human immunodeficiency virus 1 (HIV-1) by acting as a specific inhibitor of the synthesis of retroviruses encoded proteins in a codon-usage-dependent manner. Binds to tRNAs and exploits the unique viral codon bias towards A/T nucleotides. The exact inhibition mechanism is unclear: may either sequesters tRNAs, prevents their maturation via post-transcriptional processing or accelerates their deacylation. Does not inhibit reverse transcription, integration or production and nuclear export of viral RNA. May play a role in cell cycle arrest and/or induction of apoptosis in response to exogenously induced DNA damage

Validated:

a = mutation observed in both Illumina WES (Assay 1) and Hotspot (Assay 2)

Previously observed:

yes = exact match

similar = no exact match, but other mutation observed at same position

[1] previously observed variant is of unknown origin (unknown if it is somatic)

2. Somatic mutations in other genes:

ABCC2, ANGEL2, ANKRD36, APOBR, ARHGAP15, ASTN1, ATN1, ATRNL1, AXDND1, BAI1, C17orf51, C4orf50, CADM1, CCDC147, CCDC28A, CCSER1, CD163, CDC27, CIB2, CLCN1, DAB1, DDX46, DMD, DNAH3, DOHH, DYNAP, ELF2, EVI5L, FAM109B, FAT4, FLNB, FMNL3, FOXP2, FRMPD4, GLG1, GNB5, GPATCH4, GREB1, HECW1, HSD17B4, HUWE1, IL20RA, IMPG2, KCNK1, KIF18B, LCP2, MAP7D3, MATN4, MMEL1, MYO5C, N4BP2, NEB, NETO1, NLRP14, OR2D3, OR5H14, OR8H3, OTOGL, PCDH17, PCDH7, PCDHGA6, PGK1, PHKA2, PKHD1L1, POMC, POTEF, PREX2, RAD54L2, RASAL2, RBM46, RGPD8, RSPO3, RYR1, SEMA3D, SEZ6L, SIPA1L2, SLC35E2B, SLC9B1P1, TCEB3C, TOM1, TONSL, TPBG, TRAM1L1, TRPV5, TSGA10, USP49, VN1R4, WDR52, ZFC3H1, ZFP69B, ZNF280A, ZNF518B, ZNF784

Disclaimer: Findings included in this report are not intended for use in clinical application such as prognosis estimation, risk assessment, or treatment decision-making. Findings are based on research assays and may not be validated by a clinical test. Caution should be used when interpreting the findings, as they are investigational. Some tests, drugs, and biomarkers identified in this report are for research purpose only and may not be approved by the FDA for requisite use or validated for that use. We do not endorse any product, physician, procedure or other information in such content, nor do these findings substitute for professional medical advice, diagnosis or treatment. Do not disregard professional medical advice or treatment because of anything you may read in this document.

Analysis Summary : Predictive

Tier 1: FDA Approved Drugs for Colorectal Cancer

Drug	Evidence	Clinical Association			Confidence Level#	Refs	Additional Information
		Potential Increased Benefit	Potential Decreased Benefit	Potential Lack of Benefit			
Cetuximab, Panitumumab	BRAF mutation p.V600E (Somatic WES)			X	Moderate	[1][2][3]	1. Combination therapy with BRAF and EGFR inhibitors may be beneficial [4][5][6]<br>2. No benefit-reducing somatic mutation was detected in KRAS, PI3KCA, NRAS
	PTEN splice site acceptor (Somatic WES) and possible copy loss (CNV Array)		X		Weak	[7]	
	Possible KRAS copy number Gain (CNV WES)		X		Weak	[8][9][10]	
Fluorouracil, Capecitabine	SMAD4 p.R361H and possible copy loss (Somatic WES and CNV Array)		X		Weak	[11][12]	PI3K/Akt/mTOR inhibitors might restore sensitivity
	Evidence suggests possible MSI (Somatic WES and CNV), clinical confirmation strongly recommended		X		Weak	[13]	
Oxaliplatin	XRCC1 rs25487 T/T (Germline WES)		X		Weak	[14][15]	
Bevacizumab	KDR (VEGFR2) rs2305948 C/C (Germline WES)	X			Weak	[16][17]	
	CXCR2 rs2230054 C/C (Germline WES)	X					
Irinotecan	Possible MSI (Somatic WES) but not conclusive, further clinical test recommended	X			Weak	[18]	

Drugs that do not have genetic variants with reported predictive significance detected: Regorafenib, Aflibercept,

Disclaimer: Findings included in this report are not intended for use in clinical application such as prognosis estimation, risk assessment, or treatment decision-making. Findings are based on research assays and may not be validated by a clinical test. Caution should be used when interpreting the findings, as they are investigational. Some tests, drugs, and biomarkers identified in this report are for research purpose only and may not be approved by the FDA for requisite use or validated for that use. We do not endorse any product, physician, procedure or other information in such content, nor do these findings substitute for professional medical advice, diagnosis or treatment. Do not disregard professional medical advice or treatment because of anything you may read in this document.

Toxicity Prediction

Drug	Evidence	Toxicity	Confidence Level#	Additional Information	Refs
Fluorouracil, Capecitabine	DPYD: rs55886062 G/G isoform *13/*13 (Germline WES)	Elevated Toxicity	Moderate	Reduced DPYD activity	[19][20]
Irinotecan	UGT1A1: isoform *28/*28 Negative (Germline WES)	Normal	Weak		[21][22]
	UGT1A7: isoform *3/*3 Negative (Germline WES)				
	UGT1A9: isoform *22/*22 Negative (Germline WES)				
Oxaliplatin	GSTP1: rs1695 A/A and rs1138272 C/C (Germline WES)	Normal	Weak		[23]

Tier 2: Drugs that directly interact with affected gene or pathway and are predicted to have potential benefit, but not FDA-approved for colorectal cancer treatment

Drug Class	Evidence	Confidence Level#	Refs	Additional Information	Drug
BRAF Inhibitors	BRAF mutation p.V600E (Somatic WES)	Weak	[5] [18] [24]	Synergistic effect if combined with PI3K/Akt/mTOR inhibitors or MEK inhibitors due to Ras-MAPK feedback [5][25][26]	Vemurafenib, Dabrafenib
MEK Inhibitors	BRAF mutation p.V600E (Somatic WES)	Weak	[24][25] [26][27] [28]	Synergistic effect if combined with PI3K /Akt/mTOR inhibitors [26][27]	Selumetinib, Trametinib, Cobimetinib
PI3K/Akt/mTOR inhibitors	PTEN splice site acceptor and possible copy number loss (Somatic WES and CNV)	Weak	[29][30]	1. Synergistic effect if combined with BRAF inhibitors or MEK inhibitors due to Ras-MAPK feedback<br>2. Aspirin may block the PI3K pathway and improve OS<br>3. mTOR inhibitors might not be effective if mTOR loss	BYL719 (PI3K)*, Perifosine (Akt)*, MK-2206 (Akt) * , GSK-2141795(Akt)*,BKM120(PI3K)*<br>Everolimus (mTOR), Pimecrolimus (mTOR), Temsirolimus(mTOR), Rapamycin/ Sirolimus (mTOR)
	Possible mTOR loss (CNV array and CNV WES)				

* : **Not FDA Approved**

Confidence Level#: *Definitive*: FDA approved; *Strong*: NCCN guideline, or major prospective clinical trial confirmed; *Moderate*: Many (>=2) studies supported (a least one clinical study); *Weak*: few clinical studies supported, or from *in vitro*/animal studies or conflicting results

Disclaimer: Findings included in this report are not intended for use in clinical application such as prognosis estimation, risk assessment, or treatment decision-making. Findings are based on research assays and may not be validated by a clinical test. Caution should be used when interpreting the findings, as they are investigational. Some tests, drugs, and biomarkers identified in this report are for research purpose only and may not be approved by the FDA for requisite use or validated for that use. We do not endorse any product, physician, procedure or other information in such content, nor do these findings substitute for professional medical advice, diagnosis or treatment. Do not disregard professional medical advice or treatment because of anything you may read in this document.

Analysis Summary: Prognostic

Test	Method	Result	Potential Implication on PFS/OS*			Reference
			Better	Neutral	Worse	
MSI	IHC	Low likelihood of MSI		X		[26] [27]
BRAF V600	WES	Mutation V600E			X	[28] [29]
KRAS G12	WES	WT		X		
PTEN Loss	CNV	Negative		X		
HER2 Amplification	CNV	Negative		X		

***: Prognostic information does not imply type of response to therapy.**

Clinical Trial Connection

Protocol	Phase	Title	Target	Contact
NCT01750918	Phase I/II	BRAF/MEK/EGFR Inhibitor Combination Study in Colorectal Cancer	BRAF, MEK, EGFR	US GSK Clinical Trials Call Center 877-379-3718 GSKClinicalSupportHD@gsk.com
NCT01347866	Phase I	Clinical Study Of PI3K/mTOR Inhibitors In Combination With An Oral MEK Inhibitor Or Irinotecan In Patients With Advanced Cancer	MEK, PI3K/mTOR	Pfizer CT. gov Call Center 1-800-718-1021
NCT02034110	Phase II	Efficacy and Safety of the Combination Therapy of Dabrafenib and Trametinib in Subjects With BRAF V600E- Mutated Rare Cancers	BRAF, MEK	US GSK Clinical Trials Call Center 877-379-3718 GSKClinicalSupportHD@gsk.com
NCT01543698	Phase Ib/II	A Phase Ib/II Study of LGX818 in Combination With MEK162 in Adult Patients With BRAF Dependent Advanced Solid Tumors	BRAF, MEK	Novartis Pharmaceuticals 1-888-669-6682
NCT01351103	Phase I	A Study of Oral LGK974 in Patients With Malignancies Dependent on Wnt Ligands	PORCN (Wnt Signaling pathway)	Novartis Pharmaceuticals 1-888-669-6682

More clinical trial information can be found at http://clinicaltrials.gov

Disclaimer: Findings included in this report are not intended for use in clinical application such as prognosis estimation, risk assessment, or treatment decision-making. Findings are based on research assays and may not be validated by a clinical test. Caution should be used when interpreting the findings, as they are investigational. Some tests, drugs, and biomarkers identified in this report are for research purpose only and may not be approved by the FDA for requisite use or validated for that use. We do not endorse any product, physician, procedure or other information in such content, nor do these findings substitute for professional medical advice, diagnosis or treatment. Do not disregard professional medical advice or treatment because of anything you may read in this document.

Supplementary: Pathway Analysis

See the attached Pathway Analysis Summary

References

1. De Roock, W., et al., (2010) *Effects of KRAS, BRAF, NRAS, and PIK3CA mutations on the efficacy of cetuximab plus chemotherapy in chemotherapy-refractory metastatic colorectal cancer: a retrospective consortium analysis*. Lancet Oncol, 11(8): p. 753-62.
2. Mao, C., et al., (2011) *BRAF V600E mutation and resistance to anti-EGFR monoclonal antibodies in patients with metastatic colorectal cancer: a meta-analysis.* Mol Biol Rep, 38(4): p. 2219-23.
3. Tol, J., et al., (2010) *Markers for EGFR pathway activation as predictor of outcome in metastatic colorectal cancer patients treated with or without cetuximab*. Eur J Cancer, 46(11): p. 1997-2009.
4. Corcoran, R.B., et al., (2012) *EGFR-mediated re-activation of MAPK signaling contributes to insensitivity of BRAF mutant colorectal cancers to RAF inhibition with vemurafenib*. Cancer Discov, 2(3): p. 227-35.
5. Prahallad, A., et al., (2012) *Unresponsiveness of colon cancer to BRAF(V600E) inhibition through feedback activation of EGFR*. Nature, 483(7387): p. 100-3.
6. Yang, H., et al., (2012) *Antitumor activity of BRAF inhibitor vemurafenib in preclinical models of BRAF-mutant colorectal cancer.* Cancer Res, 72(3): p. 779-89.
7. Frattini M, Saletti P, Romagnani E, Martin V, Molinari F, Ghisletta M, Camponovo A, Etienne LL, Cavalli F, Mazzucchelli L. (2007) *PTEN loss of expression predicts cetuximab efficacy in metastatic colorectal cancer patients*. Br J Cancer. 97(8):1139-45. Epub 2007 Oct 16.
8. Mekenkamp LJ, Tol J, Dijkstra JR, de Krijger I, Vink-Börger ME, van Vliet S, Teerenstra S, Kamping E, Verwiel E, Koopman M, Meijer GA, van Krieken JH, Kuiper R, Punt CJ, Nagtegaal ID. (2012) *Beyond KRAS mutation status: influence of KRAS copy number status and microRNAs on clinical outcome to cetuximab in metastatic colorectal cancer patients*. BMC Cancer. 12:292.
9. Valtorta E, Misale S, Sartore-Bianchi A, Nagtegaal ID, Paraf F, Lauricella C, Dimartino V, Hobor S, Jacobs B, Ercolani C, Lamba S, Scala E, Veronese S, Laurent-Puig P, Siena S, Tejpar S, Mottolese M, Punt CJ, Gambacorta M, Bardelli A, Di Nicolantonio F. (2013) *KRAS gene amplification in colorectal cancer and impact on response to EGFR-targeted therapy.* Int J Cancer. 133(5):1259-65.
10. Hamidi H, Lu M, Chau K, Anderson L, Fejzo M, Ginther C, Linnartz R, Zubel A, Slamon DJ, Finn RS. (2014) *KRAS mutational subtype and copy number predict in vitro response of human pancreatic cancer cell lines to MEK inhibition*. Br J Cancer. 111(9):1788-801.
11. Boulay J., Mild G., Lowy A., Reuter J., Lagrange M., Terracciano L., et al. (2002) *SMAD4 is a predictive marker for 5-fluorouracil-based chemotherapy in patients with colorectal cancer.* Br J Cancer 87: 630–634.
12. Zhang B et al (2014) *Loss of Smad4 in colorectal cancer induces resistance to 5-fluorouracil through activating Akt pathway.* Br J Cancer 110(4):946-57
13. Ribic C., Sargent D., Moore M., Thibodeau S., French A., Goldberg R., et al. (2003) *Tumor microsatellite-instability status as a predictor of benefit from fluorouracil-based adjuvant chemotherapy for colon cancer*. N Engl J Med 349: 247-257
14. Lv H, Li Q, Qiu W, Xiang J, Wei H, Liang H, Sui A, Liang J. (2012) *Genetic polymorphism of XRCC1 correlated with response to oxaliplatin-based chemotherapy in advanced colorectal cancer*. Pathol Oncol Res. 18(4):1009-14
15. Kweekel DM, Antonini NF, Nortier JW, Punt CJ, Gelderblom H, Guchelaar HJ. (2009) *Explorative study to identify novel candidate genes related to oxaliplatin efficacy and toxicity using a DNA repair array.* Br J Cancer. 101(2):357-6

Disclaimer: Findings included in this report are not intended for use in clinical application such as prognosis estimation, risk assessment, or treatment decision-making. Findings are based on research assays and may not be validated by a clinical test. Caution should be used when interpreting the findings, as they are investigational. Some tests, drugs, and biomarkers identified in this report are for research purpose only and may not be approved by the FDA for requisite use or validated for that use. We do not endorse any product, physician, procedure or other information in such content, nor do these findings substitute for professional medical advice, diagnosis or treatment. Do not disregard professional medical advice or treatment because of anything you may read in this document.

16. Lambrechts D, Lenz HJ, de Haas S, Carmeliet P, Scherer SJ. (2013) *Markers of response for the antiangiogenic agent bevacizumab.* J Clin Oncol. 20;31(9):1219-30
17. Gerger, A., et al. (2011) *Pharmacogenetic angiogenesis profiling for first-line Bevacizumab plus oxaliplatin-based chemotherapy in patients with metastatic colorectal cancer*. Clin Cancer Res, 17(17),5783-92.
18. Moorcraft SY et al (2013) The role of personalized medicine in metastatic colorectal cancer: an evolving landscape Therap Adv Gastroenterol. 6(5): 381–395.
19. Morel, A., et al. (2006) *Clinical relevance of different dihydropyrimidine dehydrogenase gene single nucleotide polymorphisms on 5-fluorouracil tolerance*. Mol Cancer Ther, 5(11), 2895-904.
20. Kleibl Z, Fidlerova J, Kleiblova P, Kormunda S, Bilek M, Bouskova K, Sevcik J, Novotny J. (2009) *Influence of dihydropyrimidine dehydrogenase gene (DPYD) coding sequence variants on the development of fluoropyrimidine-related toxicity in patients with high-grade toxicity and patients with excellent tolerance of fluoropyrimidine-based chemotherapy*. Neoplasma. 56(4):303-16.
21. Tziotou, M., et al., (2014) *Polymorphisms of uridine glucuronosyltransferase gene and irinotecan toxicity: low dose does not protect from toxicity. Ecancermedicalscience*, 8: p. 428.
22. Carlini, L.E., et al., (2005) *UGT1A7 and UGT1A9 polymorphisms predict response and toxicity in colorectal cancer patients treated with capecitabine/irinotecan*. Clin Cancer Res, 11(3), 1226-36.
23. Kweekel, D.M., et al., (2009) *Explorative study to identify novel candidate genes related to oxaliplatin efficacy and toxicity using a DNA repair array*. British Journal of Cancer, 101(2), 357-362.
24. Chapman, M. S. & Miner, J. N. *Novel mitogen-activated protein kinase kinase inhibitors*. Expert Opin. Investig. Drugs 20, 209–220 (2011)
25. Santarpia, L., S.M. Lippman, and A.K. El-Naggar, (2012) *Targeting the MAPK-RAS-RAF signaling pathway in cancer therapy.* Expert Opin Ther Targets, 16(1): p. 103-19.
26. Hatzivassiliou G *et al.* (2013) *Mechanism of MEK inhibition determines efficacy in mutant KRAS- versus BRAF-driven cancers.* Nature 501(7466):232-6
27. Maira SM *et al* (2012) *Identification and characterization of NVP-BKM120, an orally available pan-class I PI3-kinase inhibitor.* Mol Cancer Ther. 11(2):317-28.
28. Solit DB, et al (2006) BRAF mutation predicts sensitivity to MEK inhibition. Nature. 439(7074):358-62.
29. Mao M., Tian F., Mariadason J., Tsao L., Lemos R., Jr, Dayyani F., et al. (2012) Resistance to BRAF inhibition in BRAF-mutant colon cancer can be overcome with PI3K inhibition or demethylating agents. Clin Cancer Res 19: 657-667.
30. Liao X., Lochhead P., Nishihara R., Morikawa T., Kuchiba A., Yamauchi M., et al. (2012) Aspirin use, tumor PIK3CA mutation, and colorectal-cancer survival. N Engl J Med 367: 1596-1606.
31. Grady W., Carethers J. (2008) *Genomic and epigenetic instability in colorectal cancer pathogenesis.* Gastroenterology 135: 1079-1099
32. Merok MA *et al* (2013) *Microsatellite instability has a positive prognostic impact on stage II colorectal cancer after complete resection: results from a large, consecutive Norwegian series.* Ann Oncol. 24(5):1274-82.
33. Eklöf V *et al* (2013) *The prognostic role of KRAS, BRAF, PIK3CA and PTEN in colorectal cancer.* Br J Cancer. 108(10):2153-63.

Disclaimer: Findings included in this report are not intended for use in clinical application such as prognosis estimation, risk assessment, or treatment decision-making. Findings are based on research assays and may not be validated by a clinical test. Caution should be used when interpreting the findings, as they are investigational. Some tests, drugs, and biomarkers identified in this report are for research purpose only and may not be approved by the FDA for requisite use or validated for that use. We do not endorse any product, physician, procedure or other information in such content, nor do these findings substitute for professional medical advice, diagnosis or treatment. Do not disregard professional medical advice or treatment because of anything you may read in this document.

Signaling Pathway Analysis P0000

The TCGA study, focused on colorectal cancers, has defined 5 significantly altered pathways in colorectal cancer. Described below are alterations found in these and some other critical pathways. A detailed description of how these deregulate growth is also provided.

ERK Pathway
Most colorectal cancers harbor mutations in the ERK pathway. Receptor tyrosine kinase activation drives RAS (KRAS, NRAS, HRAS) to stimulate RAF (BRAF, CRAF, ARAF) (Figure 1). This drives MEK and ERK activity, leading to proliferation and survival of the cell. This patient has a BRAF p.V600E mutation, a likely driver for P0000. Here, the ERK pathway is activated downstream of receptor tyrosine kinase (Figure 1), possibly rendering this patient unresponsive to anti-EGFR antibody therapy, if give alone.

AKT Pathway
PI3K drives signaling through the AKT pathway. The actions of PI3K are reversed through dephosphorylation of phosphatidylinositol lipids at the 3' position. The major phosphatase responsible for this step is PTEN. P0000 harbors a mutation that will allow for the acquisition of an extra splice site. This is predicted to alter the function of PTEN and allow for enhanced signaling through AKT.

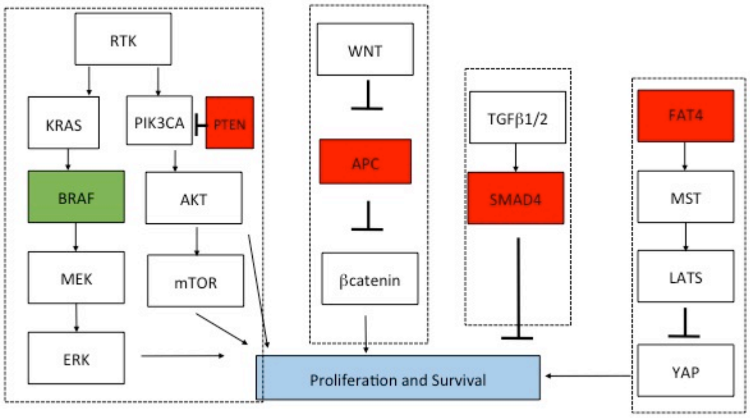

Figure 1. Major signaling pathways influencing colorectal cancer 14058. ***Green*** **indicates an activated oncogene and** ***red*** **indicates inactivation of a tumor suppressor.**

TGF-β Pathway
Activation of the TGF-β receptor, comprised of TGFβR1 and TGFβR2, leads to phosphorylation of SMAD2/3 and association of the active SMAD2/3-SMAD4 complex. This transcription factor drives a growth-suppressive transcriptional program. In this patient, there is a loss of function mutation in SMAD4 (p.R361H). Therefore, TGF-β receptor signaling is unable to stimulate SMAD-dependent growth suppression through this pathway.

Wnt Pathway
According to the TCGA data for colorectal cancer, 70% of these tumors have APC mutations. Two mutations in APC were identified, p.T1556fs*3 and p.R232*. These mutations will generate truncated proteins and will likely lead to stabilization of β-catenin. This would drive cell proliferation.

Hippo Pathway/Planar Cell Polarity Pathway
A mutation was found in the FAT4 gene, which is a known tumor suppressor. FAT4 may be a component of the Hippo signaling pathway, which normally suppresses cell proliferation when the pathway is active. Additionally, FAT4 may interact with components of the Wnt/PCP pathway. Loss of FAT4 activity may lead to increased cell proliferation through loss of Hippo signaling or gain of Wnt/PCP signaling.

Disclaimer: Findings included in this report are not intended for use in clinical application such as prognosis estimation, risk assessment, or treatment decision-making. Findings are based on research assays and may not be validated by a clinical test. Caution should be used when interpreting the findings, as they are investigational. Some tests, drugs, and biomarkers identified in this report are for research purpose only and may not be approved by the FDA for requisite use or validated for that use. We do not endorse any product, physician, procedure or other information in such content, nor do these findings substitute for professional medical advice, diagnosis or treatment. Do not disregard professional medical advice or treatment because of anything you may read in this document.
