# Supplementary material for: Development and clinical application of an integrative genomic approach to personalized cancer therapy
Source: Genome Med. 2016 Jun 1;8:62. doi: 10.1186/s13073-016-0313-0 (PMC4888213; doi:10.1186/s13073-016-0313-0)
Supplement: Supplementary file 12 — CLPB-NADSYN1 gene fusion in patient P0002. a Long-range PCR confirms CLPB-NADSYN1 gene fusion. b Genomic breakpoint of CLPB-NADSYN1 gene fusion. (ZIP 150 kb) [file 13073_2016_313_MOESM12_ESM.zip › Fig S9B.pptx]

## Slide 1
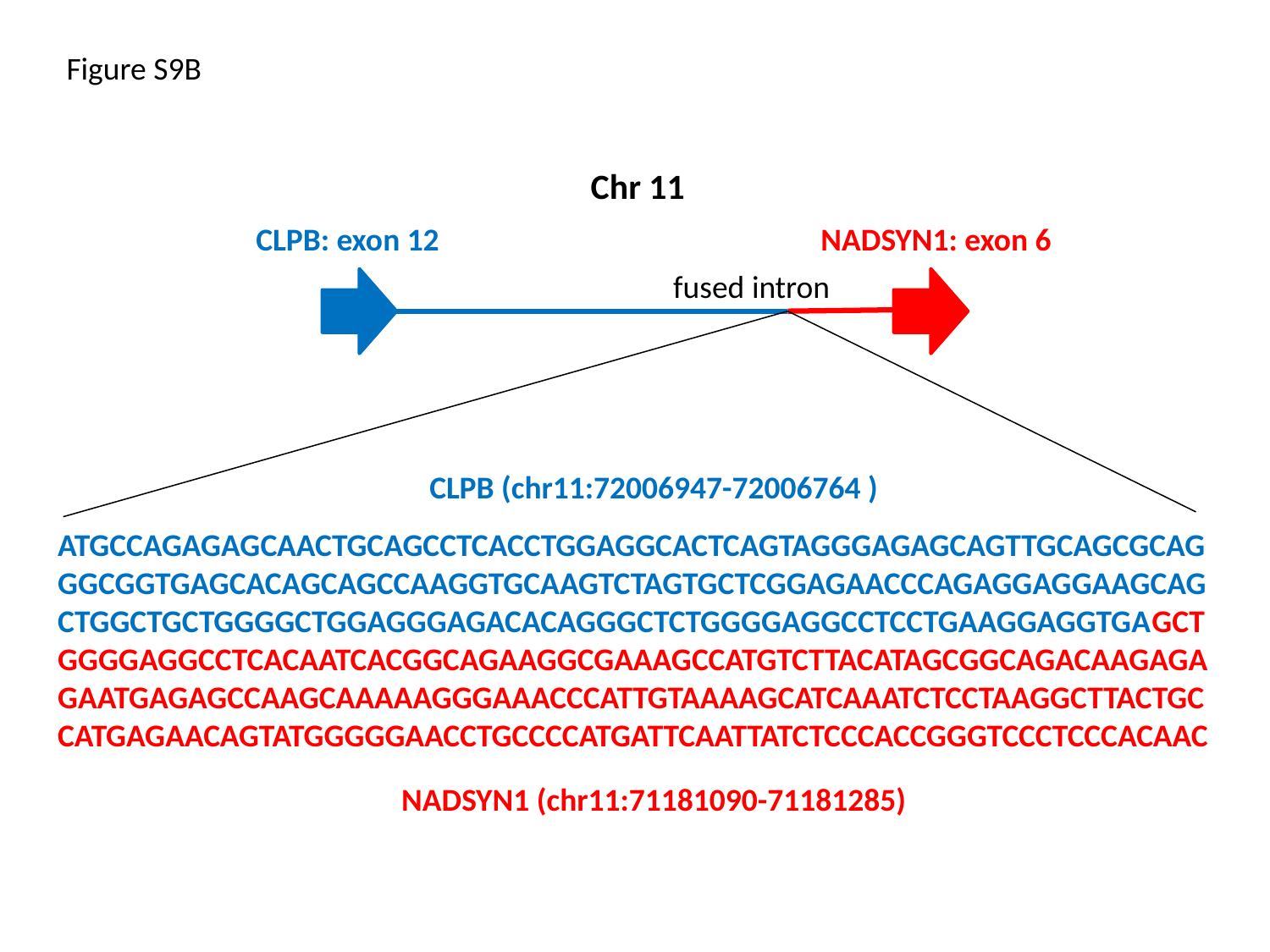

Figure S9B
Chr 11
CLPB: exon 12
NADSYN1: exon 6
fused intron
CLPB (chr11:72006947-72006764 )
ATGCCAGAGAGCAACTGCAGCCTCACCTGGAGGCACTCAGTAGGGAGAGCAGTTGCAGCGCAGGGCGGTGAGCACAGCAGCCAAGGTGCAAGTCTAGTGCTCGGAGAACCCAGAGGAGGAAGCAGCTGGCTGCTGGGGCTGGAGGGAGACACAGGGCTCTGGGGAGGCCTCCTGAAGGAGGTGAGCTGGGGAGGCCTCACAATCACGGCAGAAGGCGAAAGCCATGTCTTACATAGCGGCAGACAAGAGAGAATGAGAGCCAAGCAAAAAGGGAAACCCATTGTAAAAGCATCAAATCTCCTAAGGCTTACTGCCATGAGAACAGTATGGGGGAACCTGCCCCATGATTCAATTATCTCCCACCGGGTCCCTCCCACAAC
NADSYN1 (chr11:71181090-71181285)
