# Supplementary material for: Development and clinical application of an integrative genomic approach to personalized cancer therapy
Source: Genome Med. 2016 Jun 1;8:62. doi: 10.1186/s13073-016-0313-0 (PMC4888213; doi:10.1186/s13073-016-0313-0)
Supplement: Supplementary file 14 — Sanger sequencing validation results of novel somatic EGFR mutation p.D587H (chr7:55233009G>C) in patient P0015. Sanger sequencing was carried out on normal and tumor DNA from this patient using forward and reverse primers (Beckman Coulter Genomics, Danvers, Massachusetts). Traces shown are as displayed by 4Peaks visualization software for Mac OS X (http://nucleobytes.com/4peaks). Two replicate sequencing reactions were carried out for each primer and sample combination, yielding the same result (second replicate not shown). Base numbering shown is relative to priming site. The allelic fraction of p.D587H in tumor was 19.4 % (387/1998 reads have variant) in targeted panel sequencing, explaining the relatively small size of the Sanger peak for the alternate allele. (PPTX 240 kb) [file 13073_2016_313_MOESM14_ESM.pptx]

## Slide 1
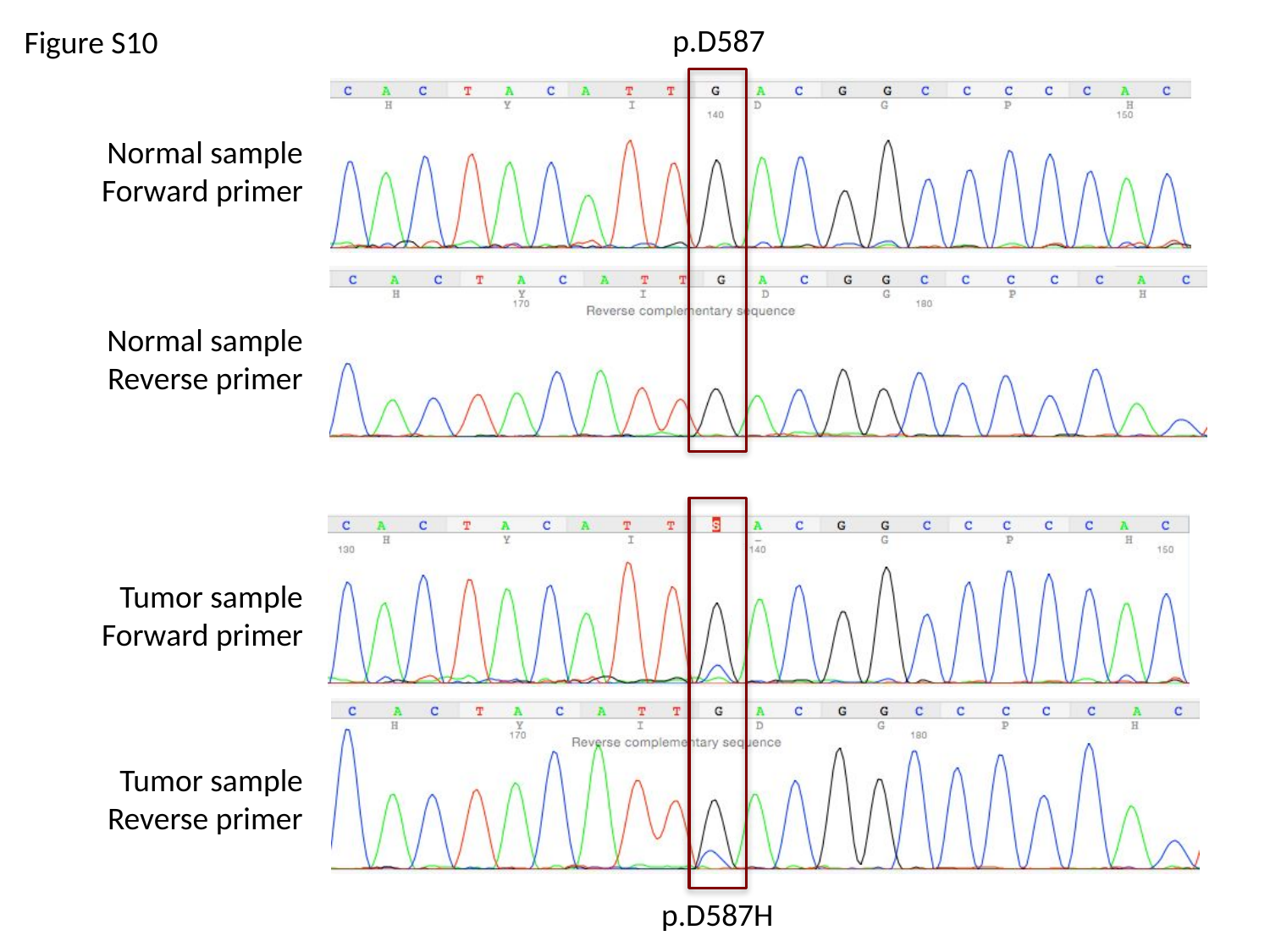

p.D587
Figure S10
Normal sample
Forward primer
Normal sample
Reverse primer
Tumor sample
Forward primer
Tumor sample
Reverse primer
p.D587H
